# Supplementary material for: New 11-Methoxymethylgermacranolides from the Whole Plant of Carpesium divaricatum
Source: Molecules. 2022 Sep 14;27(18):5991. doi: 10.3390/molecules27185991 (PMC9505371; doi:10.3390/molecules27185991)
Supplement: Supplementary file 1 [file molecules-27-05991-s001.zip › molecules-1918445-supplementary.pdf]

Contents of Supplementary Information

	contents
C1	The relevant data of ECD calculations of compounds **5Ja** and **7Ja**
Table S1	Crystal data and structure refinement for **1**.
Table S2	Fractional atomic coordinates ($\times10^4$) and equivalent isotropic displacement parameters ($Å^2\times10^3$) for **1**. U_{eq} is defined as 1/3 of of the trace of the orthogonalised U_{IJ} tensor.
Table S3	Anisotropic displacement parameters ($Å^2\times10^3$) for **1**. The anisotropic displacement factor exponent takes the form: -2π2[h2a*2U11+...+2hka×b×U12]
Table S4	Bond lengths for **1**.
Table S5	Bond angles for **1**.
Table S6	Torsion angles for **1**.
Table S7	Hydrogen atom coordinates ($Å\times10^4$) and isotropic displacement parameters ($Å^2\times10^3$) for **1**.
	Computational details
Table S8	Conformational analysis of **5Ja**
Table S9	Conformational analysis of **7Ja**
**Figure S**1.1	^{1}H NMR spectrum of compound **1** in CD_3OD
**Figure S**1.2	^{13}C NMR spectrum of compound **1** in CD_3OD
**Figure S**1.3	^{1}H-^{1}H COSY spectrum of compound **1** in CD_3OD
**Figure S**1.4	HSQC spectrum of compound **1** in CD_3OD
**Figure S**1.5	HMBC spectrum of compound **1** in CD_3OD
**Figure S**1.6	NOESY spectrum of compound **1** in CD_3OD
**Figure S**1.7	UV spectrum of compound **1**
**Figure S**1.8	IR spectrum of compound **1**
**Figure S**1.9	HRESIMS spectrum of compound **1**
**Figure S**1.10	ECD spectrum of compound **1**
**Figure S**2.1	^{1}H NMR spectrum of compound **2** in CD_3OD
**Figure S**2.2	^{13}C NMR spectrum of compound **2** in CD_3OD
**Figure S**2.3	^{1}H-^{1}H COSY spectrum of compound **2** in CD_3OD
**Figure S**2.4	HSQC spectrum of compound **2** in CD_3OD
**Figure S**2.5	HMBC spectrum of compound **2** in CD_3OD
**Figure S**2.6	NOESY spectrum of compound **2** in CD_3OD
**Figure S**2.7	UV spectrum of compound **2**
**Figure S**2.8	IR spectrum of compound **2**
**Figure S**2.9	HRESIMS spectrum of compound **2**
**Figure S**2.10	ECD spectrum of compound **2**
**Figure S**3.1	^{1}H NMR spectrum of compound **3** in CD_3OD
**Figure S**3.2	^{13}C NMR spectrum of compound **3** in CD_3OD
**Figure S**3.3	^{1}H-^{1}H COSY spectrum of compound **3** in CD_3OD
**Figure S**3.4	HSQC spectrum of compound **3** in CD_3OD
**Figure S**3.5	HMBC spectrum of compound **3** in CD_3OD

**Figure S**3.6	ROESY spectrum of compound **3** in CD_3OD
**Figure S**3.7	UV spectrum of compound **3**
**Figure S**3.8	IR spectrum of compound **3**
**Figure S**3.9	HRESIMS spectrum of compound **3**
**Figure S**3.10	ECD spectrum of compound **3**
**Figure S**4.1	^1H NMR spectrum of compound **4** in CD_3OD
**Figure S**4.2	^{13}C NMR spectrum of compound **4** in CD_3OD
**Figure S**4.3	^1H-^1H COSY spectrum of compound **4** in CD_3OD
**Figure S**4.4	HSQC spectrum of compound **4** in CD_3OD
**Figure S**4.5	HMBC spectrum of compound **4** in CD_3OD
**Figure S**4.6	NOESY spectrum of compound **4** in CD_3OD
**Figure S**4.7	UV spectrum of compound **4**
**Figure S**4.8	IR spectrum of compound **4**
**Figure S**4.9	HRESIMS spectrum of compound **4**
**Figure S**4.10	ECD spectrum of compound **4**
**Figure S**5.1	^1H NMR spectrum of compound **5** in CD_3OD
**Figure S**5.2	^{13}C NMR spectrum of compound **5** in CD_3OD
**Figure S**5.3	^1H-^1H COSY spectrum of compound **5** in CD_3OD
**Figure S**5.4	HSQC spectrum of compound **5** in CD_3OD
**Figure S**5.5	HMBC spectrum of compound **5** in CD_3OD
**Figure S**5.6	NOESY spectrum of compound **5** in CD_3OD
**Figure S**5.7	UV spectrum of compound **5**
**Figure S**5.8	IR spectrum of compound **5**
**Figure S**5.9	HRESIMS spectrum of compound **5**
**Figure S**5.10	ECD spectrum of compound **5**
**Figure S**6.1	^1H NMR spectrum of compound **6** in CD_3OD
**Figure S**6.2	^{13}C NMR spectrum of compound **6** in CD_3OD
**Figure S**6.3	^1H-^1H COSY spectrum of compound **6** in CD_3OD
**Figure S**6.4	HSQC spectrum of compound **6** in CD_3OD
**Figure S**6.5	HMBC spectrum of compound **6** in CD_3OD
**Figure S**6.6	ROESY spectrum of compound **6** in CD_3OD
**Figure S**6.7	UV spectrum of compound **6**
**Figure S**6.8	IR spectrum of compound **6**
**Figure S**6.9	HRESIMS spectrum of compound **6**
**Figure S**6.10	ECD spectrum of compound **6**
**Figure S**7.1	^1H NMR spectrum of compound **7** in CD_3OD
**Figure S**7.2	^{13}C NMR spectrum of compound **7** in CD_3OD
**Figure S**7.3	^1H-^1H COSY spectrum of compound **7** in CD_3OD
**Figure S**7.4	HSQC spectrum of compound **7** in CD_3OD
**Figure S**7.5	HMBC spectrum of compound **7** in CD_3OD
**Figure S**7.6	ROESY spectrum of compound **7** in CD_3OD
**Figure S**7.7	UV spectrum of compound **7**
**Figure S**7.8	IR spectrum of compound **7**

**Figure S**7.9	HRESIMS spectrum of compound **7**
**Figure S**7.10	ECD spectrum of compound **7**
**Figure S**8.1	^{1}H NMR spectrum of compound **8** in CD_3OD
**Figure S**8.2	^{13}C NMR spectrum of compound **8** in CD_3OD
**Figure S**8.3	^{1}H-^{1}H COSY spectrum of compound **8** in CD_3OD
**Figure S**8.4	HSQC spectrum of compound **8** in CD_3OD
**Figure S**8.5	HMBC spectrum of compound **8** in CD_3OD
**Figure S**8.6	NOESY spectrum of compound **8** in CD_3OD
**Figure S**8.7	UV spectrum of compound **8**
**Figure S**8.8	IR spectrum of compound **8**
**Figure S**8.9	HRESIMS spectrum of compound **8**
**Figure S**8.10	ECD spectrum of compound **8**

C1: The relevant data of ECD calculations of compounds **5Ja** and **7Ja**

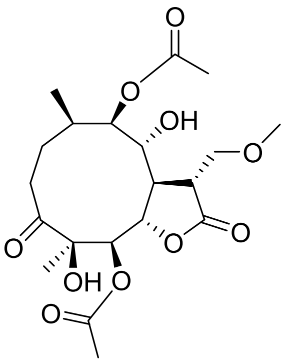

A simplified structure **5Ja**

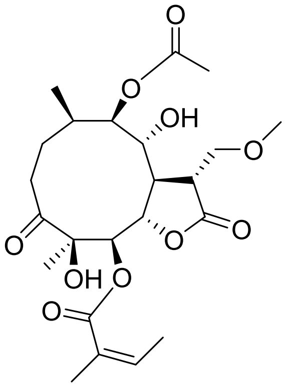

A simplified structure **7Ja**

Table S1. Crystal data and structure refinement for **1**.

Identification code	exp_5475
Empirical formula	$C_{25}H_{38}O_{10}$
Formula weight	498.55
Temperature/K	110.7(2)
Crystal system	monoclinic
Space group	P21
a/Å, b/Å, c/Å	9.0365(3), 11.1281(3), 13.1083(4)
α/°, β/°, γ/°	90.00, 97.237(3), 90.00
Volume/Å^3	1307.66(7)
Z	2
ρ_{calc}/ mg mm^{-3}	1.266
μ/mm^{-1}	0.813
F(000)	536
Crystal size / mm^3	0.25 × 0.22 × 0.13

2Θ range for data collection	10.46 to 142.04°
Index ranges	-7 ≤ h ≤ 11, -13 ≤ k ≤ 13, -15 ≤ l ≤ 15
Reflections collected	8728
Independent reflections	4912[R(int) = 0.0283
Data/restraints/parameters	4912/1/325
Goodness-of-fit on F2	1.035
Final R indexes [I>2σ (I) i.e. F0>4σ (F0)]	R_1 = 0.0327, wR_2 = 0.0818
Final R indexes [all data]	R_1 = 0.0341, wR_2 = 0.0831
Largest diff. peak/hole / e Å^{-3}	0.210/-0.177
Flack Parameters	-0.03(12)
Completeness	0.9988

Table S2. Fractional atomic coordinates (×10^4) and equivalent isotropic displacement parameters (Å^2×10^3) for **1**. U_{eq} is defined as 1/3 of of the trace of the orthogonalised U_{IJ} tensor.

Atom	*x*	*y*	*z*	U(eq)
O7	1357.0(11)	8588.5(11)	1777.4(8)	16.3(2)
O9	328.3(15)	11800.4(13)	-753.5(10)	28.3(3)
O1	-3978.8(12)	9395.3(10)	-183.6(8)	14.8(2)
O8	-1362.7(13)	10552.8(10)	-259.0(8)	18.2(2)
O5	1478.4(11)	8554.3(10)	3817.9(8)	14.7(2)
O4	-3287.9(13)	10795.1(11)	2776.5(9)	21.2(3)
O3	-3026.5(13)	7944.2(9)	1652.1(9)	17.0(2)
O6	3809.4(13)	9307.5(11)	4111.5(10)	21.9(3)
O10	434.8(15)	12776.9(12)	1634.7(12)	32.2(3)
O2	-5288.8(14)	11105.8(11)	-500.2(10)	27.2(3)
C3	-3104.9(16)	9723.7(15)	2692.8(12)	15.7(3)
C16	-4995.3(18)	10097.6(15)	-740.0(13)	17.3(3)
C4	-2456.1(18)	8947.9(15)	3589.6(12)	16.9(3)
C22	4451.5(19)	6884.1(16)	5026.2(13)	20.5(3)
C17	-5722(2)	9410.7(16)	-1669.5(13)	22.4(4)
C1	-3183.5(17)	9870.8(14)	766.1(11)	13.5(3)
C9	-400.4(17)	10242.4(14)	1504.3(12)	13.2(3)
C20	2973.2(17)	8465.1(15)	4061.6(11)	14.5(3)
C10	-1543.0(17)	9777.9(14)	619.1(11)	13.3(3)
C6	-81.9(18)	10337.7(15)	3947.2(12)	18.2(3)
C8	218.0(16)	9208.3(13)	2224.2(12)	13.0(3)
C24	5179(2)	7683.0(18)	5857.6(14)	26.2(4)
C21	3446.0(18)	7184.8(14)	4235.3(12)	16.3(3)
C5	-1332.1(18)	9618.9(16)	4364.4(12)	19.8(3)
C2	-3656.3(17)	9107.6(14)	1656.8(12)	15.4(3)

C11	-65.4(19)	11146.7(15)	-108.4(13)	19.1(3)
C7	916.0(17)	9642.5(14)	3285.0(12)	14.5(3)
C15	893(2)	10919.6(18)	4864.3(14)	27.2(4)
C19	-6595(2)	10238.8(18)	-2456.6(14)	31.5(4)
C18	-6715(3)	8425.9(19)	-1312.3(18)	42.6(6)
C23	2758(2)	6288.0(15)	3449.8(13)	22.0(3)
C13	1549.3(19)	11952.0(16)	1440.3(14)	23.4(4)
C12	778.3(17)	10852.5(15)	931.9(13)	17.3(3)
C25	1026(3)	13747.8(18)	2262.8(16)	34.9(5)
C14	-5359.4(18)	9059.8(18)	1597.8(14)	23.3(4)

Table S3. Anisotropic displacement parameters ($Å^2 \times 10^3$) for **1**. The anisotropic displacement factor exponent takes the form: $-2\pi^2[h^2a^{*2}U_{11}+...+2hka\times b\times U_{12}]$

**Atom**	**U11**	**U22**	**U33**	**U23**	**U13**	**U12**
O7	12.7(5)	16.1(5)	19.7(6)	-0.1(5)	1.0(4)	1.7(4)
O9	25.1(7)	31.8(7)	27.1(7)	15.1(6)	0.5(5)	-4.3(5)
O1	13.1(5)	14.8(5)	14.7(5)	-2.0(4)	-4.9(4)	0.9(4)
O8	18.1(6)	20.8(6)	14.6(5)	5.5(4)	-2.2(4)	-2.3(4)
O5	12.8(5)	14.5(5)	15.7(5)	4.3(4)	-2.9(4)	-0.6(4)
O4	20.6(6)	21.5(6)	21.0(6)	-4.1(5)	0.7(5)	6.1(5)
O3	17.7(5)	11.6(5)	20.2(6)	-0.7(4)	-3.1(4)	-2.4(4)
O6	15.3(6)	16.9(6)	31.6(7)	-0.4(5)	-4.9(5)	-2.6(5)
O10	25.2(7)	18.7(6)	49.4(9)	-6.9(6)	-8.7(6)	-3.7(5)
O2	32.2(7)	19.4(6)	25.3(6)	-3.4(5)	-14.7(5)	9.2(5)
C3	7.4(7)	23.7(8)	16.4(7)	-0.4(6)	3.4(5)	1.6(6)
C16	16.0(8)	16.3(8)	18.6(8)	-0.5(6)	-2.2(6)	1.7(6)
C4	13.8(7)	22.7(8)	14.0(7)	0.0(6)	1.0(6)	0.5(6)
C22	17.7(8)	20.1(8)	23.5(8)	6.0(7)	1.7(6)	4.2(6)
C17	22.3(8)	19.7(8)	22.0(8)	-6.7(7)	-9.9(7)	5.8(7)
C1	12.2(7)	13.2(7)	13.5(7)	-4.1(6)	-4.7(5)	1.4(5)
C9	10.3(7)	13.0(7)	15.1(7)	0.4(6)	-2.9(6)	-0.9(6)
C20	14.3(7)	18.0(8)	10.6(6)	-0.6(6)	-0.9(5)	0.3(6)
C10	13.8(7)	13.7(7)	11.6(7)	2.6(6)	-1.1(6)	-1.0(6)
C6	19.3(8)	17.9(8)	15.6(7)	-3.4(6)	-4.6(6)	2.0(6)
C8	10.2(7)	12.6(7)	15.8(7)	1.2(6)	0.0(6)	0.0(6)
C24	22.0(9)	31.7(10)	22.5(9)	6.5(8)	-6.5(7)	-0.7(7)
C21	15.2(8)	17.0(8)	16.9(7)	1.5(6)	3.1(6)	0.0(6)
C5	19.7(8)	26.9(9)	11.9(7)	-1.4(6)	-1.4(6)	4.2(7)
C2	9.6(7)	17.2(8)	18.8(8)	-0.7(6)	-0.2(6)	0.8(6)
C11	16.6(7)	17.7(7)	22.4(8)	3.9(7)	0.7(6)	0.6(6)

C7	13.7(7)	12.2(7)	16.4(7)	2.9(6)	-3.3(6)	-0.6(6)
C15	28.8(9)	28.1(9)	22.8(9)	-9.5(7)	-4.3(7)	-0.3(8)
C19	37.7(11)	28.4(9)	23.1(9)	-3.4(8)	-16.8(8)	4.8(8)
C18	51.4(13)	25.7(10)	42.2(12)	-0.1(9)	-26.9(10)	-13.2(10)
C23	27.0(9)	15.7(8)	22.6(8)	-1.8(7)	1.0(7)	0.1(7)
C13	16.1(8)	21.2(8)	30.5(9)	7.4(7)	-6.5(7)	-6.0(6)
C12	12.3(7)	16.7(7)	22.0(8)	5.5(6)	-0.8(6)	-0.1(6)
C25	43.3(12)	24(1)	34.1(10)	-2.6(8)	-8.1(9)	-9.7(8)
C14	10.7(8)	35.5(10)	23.4(9)	-0.6(7)	0.3(6)	-2.4(7)

Table S4. Bond lengths for **1**.

Atom	Atom	Length/Å	Atom	Atom	Length/Å
O7	C8	1.4248(18)	C22	C24	1.493(3)
O9	C11	1.203(2)	C22	C21	1.332(2)
O1	C16	1.3482(19)	C17	C19	1.527(2)
O1	C1	1.4563(17)	C17	C18	1.527(3)
O8	C10	1.4637(18)	C1	C10	1.522(2)
O8	C11	1.339(2)	C1	C2	1.547(2)
O5	C20	1.3520(18)	C9	C10	1.542(2)
O5	C7	1.4571(18)	C9	C8	1.547(2)
O4	C3	1.211(2)	C9	C12	1.536(2)
O3	C2	1.4146(19)	C20	C21	1.497(2)
O6	C20	1.201(2)	C6	C5	1.540(2)
O10	C13	1.409(2)	C6	C7	1.537(2)
O10	C25	1.421(2)	C6	C15	1.541(2)
O2	C16	1.204(2)	C8	C7	1.531(2)
C3	C4	1.516(2)	C21	C23	1.511(2)
C3	C2	1.547(2)	C2	C14	1.532(2)
C16	C17	1.516(2)	C11	C12	1.512(2)
C4	C5	1.537(2)	C13	C12	1.520(2)

Table S5. Bond angles for **1**.

Atom	Atom	Atom	Angle/°	Atom	Atom	Atom	Angle/°
C16	O1	C1	118.75(12)	C5	C6	C15	108.32(14)
C11	O8	C10	110.92(12)	C7	C6	C5	116.62(13)
C20	O5	C7	116.88(12)	C7	C6	C15	109.48(14)
C13	O10	C25	112.03(15)	O7	C8	C9	109.48(12)
O4	C3	C4	122.32(15)	O7	C8	C7	106.85(12)

O4	C3	C2	118.92(15)	C7	C8	C9	113.27(12)
C4	C3	C2	118.61(14)	C22	C21	C20	120.74(15)
O1	C16	C17	109.82(13)	C22	C21	C23	123.12(15)
O2	C16	O1	123.82(15)	C20	C21	C23	116.11(14)
O2	C16	C17	126.33(15)	C4	C5	C6	118.11(13)
C3	C4	C5	113.25(14)	O3	C2	C3	108.92(13)
C21	C22	C24	127.79(16)	O3	C2	C1	110.47(12)
C16	C17	C19	111.93(14)	O3	C2	C14	111.73(13)
C16	C17	C18	109.10(15)	C3	C2	C1	109.29(12)
C18	C17	C19	111.52(16)	C14	C2	C3	105.67(13)
O1	C1	C10	104.52(12)	C14	C2	C1	110.62(13)
O1	C1	C2	106.99(12)	O9	C11	O8	121.74(16)
C10	C1	C2	115.10(12)	O9	C11	C12	127.22(16)
C10	C9	C8	111.45(12)	O8	C11	C12	111.03(13)
C12	C9	C10	102.73(12)	O5	C7	C6	109.97(12)
C12	C9	C8	114.03(13)	O5	C7	C8	104.60(12)
O5	C20	C21	111.31(13)	C8	C7	C6	117.90(13)
O6	C20	O5	123.97(15)	O10	C13	C12	107.76(13)
O6	C20	C21	124.70(14)	C11	C12	C9	103.55(13)
O8	C10	C1	105.44(12)	C11	C12	C13	111.86(14)
O8	C10	C9	105.30(12)	C13	C12	C9	116.70(14)
C1	C10	C9	116.90(13)				

Table S6. Torsion angles for **1**.

**A**	**B**	**C**	**D**	**Angle/°**
O7	C8	C7	O5	-56.98(14)
O7	C8	C7	C6	-179.48(13)
O9	C11	C12	C9	166.79(17)
O9	C11	C12	C13	40.3(2)
O1	C16	C17	C19	-166.24(16)
O1	C16	C17	C18	69.84(18)
O1	C1	C10	O8	62.35(14)
O1	C1	C10	C9	178.91(12)
O1	C1	C2	O3	71.59(15)
O1	C1	C2	C3	-168.59(11)
O1	C1	C2	C14	-52.64(16)
O8	C11	C12	C9	-14.50(18)
O8	C11	C12	C13	-141.00(15)

O5	C20	C21	C22	-136.38(15)
O5	C20	C21	C23	45.54(18)
O4	C3	C4	C5	-31.3(2)
O4	C3	C2	O3	166.35(14)
O4	C3	C2	C1	45.58(18)
O4	C3	C2	C14	-73.48(18)
O6	C20	C21	C22	44.9(2)
O6	C20	C21	C23	-133.14(16)
O10	C13	C12	C9	-54.60(18)
O10	C13	C12	C11	64.38(18)
O2	C16	C17	C19	15.7(3)
O2	C16	C17	C18	-108.2(2)
C3	C4	C5	C6	-49.35(19)
C16	O1	C1	C10	-122.68(14)
C16	O1	C1	C2	114.81(14)
C4	C3	C2	O3	-18.02(18)
C4	C3	C2	C1	-138.78(14)
C4	C3	C2	C14	102.15(16)
C1	O1	C16	O2	0.2(2)
C1	O1	C16	C17	-177.94(14)
C9	C8	C7	O5	-177.61(12)
C9	C8	C7	C6	59.89(17)
C20	O5	C7	C6	-117.64(14)
C20	O5	C7	C8	114.84(13)
C10	O8	C11	O9	177.26(16)
C10	O8	C11	C12	-1.53(18)
C10	C1	C2	O3	-44.05(17)
C10	C1	C2	C3	75.77(15)
C10	C1	C2	C14	-168.28(13)
C10	C9	C8	O7	80.58(15)
C10	C9	C8	C7	-160.30(13)
C10	C9	C12	C11	23.22(15)
C10	C9	C12	C13	146.59(13)
C8	C9	C10	O8	-147.11(12)
C8	C9	C10	C1	96.26(16)
C8	C9	C12	C11	143.96(13)
C8	C9	C12	C13	-92.67(16)
C24	C22	C21	C20	3.5(3)
C24	C22	C21	C23	-178.58(17)
C5	C6	C7	O5	-51.08(17)

C5	C6	C7	C8	68.65(18)
C2	C3	C4	C5	153.18(13)
C2	C1	C10	O8	179.39(12)
C2	C1	C10	C9	-64.05(18)
C11	O8	C10	C1	141.18(13)
C11	O8	C10	C9	16.97(16)
C7	O5	C20	O6	19.8(2)
C7	O5	C20	C21	-158.93(12)
C7	C6	C5	C4	-55.82(19)
C15	C6	C5	C4	-179.80(14)
C15	C6	C7	O5	72.30(16)
C15	C6	C7	C8	-167.97(14)
C12	C9	C10	O8	-24.61(15)
C12	C9	C10	C1	-141.24(14)
C12	C9	C8	O7	-35.17(17)
C12	C9	C8	C7	83.96(16)
C25	O10	C13	C12	170.14(15)

Table S7. Hydrogen atom coordinates (Å×10^4) and isotropic displacement parameters (Å^2×10^3) for **1**.

Atom	*x*	*y*	*z*	U(eq)
H7	1006	7981	1486	24
H3	-3575	7507	1268	26
H4A	-1965	8259	3327	20
H4B	-3263	8650	3943	20
H22	4734	6080	5065	25
H17	-4931	9024	-2000	27
H1	-3456	10714	852	16
H9	-874	10845	1902	16
H10	-1305	8946	456	16
H6	-557	10990	3523	22
H8	-592	8646	2309	16
H24A	5086	7333	6516	39
H24B	4704	8456	5810	39
H24C	6216	7774	5782	39
H5A	-866	9034	4852	24
H5B	-1888	10170	4745	24
H7A	1772	10152	3190	17
H15A	1641	11415	4615	41
H15B	1368	10302	5301	41
H15C	280	11404	5248	41

H19A	-7397	10608	-2156	47
H19B	-6994	9779	-3048	47
H19C	-5943	10850	-2661	47
H18A	-6125	7901	-842	64
H18B	-7156	7973	-1896	64
H18C	-7488	8784	-974	64
H23A	2967	6523	2778	33
H23B	1698	6265	3461	33
H23C	3171	5506	3611	33
H13A	2146	11727	2079	28
H13B	2201	12310	991	28
H12	1545	10256	832	21
H25A	1811	14128	1952	52
H25B	1415	13450	2930	52
H25C	250	14321	2330	52
H14A	-5762	8621	996	35
H14B	-5752	9862	1562	35
H14C	-5631	8666	2199	35

Computational details

In general, conformational analyses were carried out via random searching in the Sybyl-X 2.0 using the MMFF94S force field with an energy cutoff of 2.5 kcal/mol.[1] Subsequently, the conformerswere re-optimized using DFT at the b3lyp/6-31+g(d) level in gasphase by the GAUSSIAN 09 program.[2] The energies, oscillator strengths, and rotational strengths (velocity) of the first 100 electronic excitations were calculated using the TDDFT methodology at the b3lyp/6-311++g (d,p) level in vacuum. The ECD spectra were simulated by the overlapping Gaussian function (half the bandwidth at 1/e peak height, $\sigma = 0.3$ for 1 and hjb-24, and 0.35 for hjb-10).[3]To get the final spectra, the simulated spectra ofthe conformers were average according to the Boltzmann distribution theory and their relative Gibbs free energy (ΔG).Theoretical ECD spectrum of the corresponding enantiomerwere obtained by directly inverse of the ECD spectrum of the above-mentioned compounds,

respectively. By comparing the experiment spectrum with the calculated ECD spectra, the eight chiral centers of **5J** and **7J** were determined to be 4*R*, 5*R*, 6*S*, 7*R*, 8*R*, 9*R*, 10*R*, and 11*R*.

Reference:

(1) Sybyl Software, version X 2.0; Tripos Associates Inc.: St. Louis, MO, 2013.

(2) Frisch, M. J.; Trucks, G. W.; Schlegel, H. B.; Scuseria, G. E.; Robb, M. A.; Cheeseman, J. R.; Scalmani, G.; Barone, V.; Mennucci, B.; Petersson, G. A.; Nakatsuji, H.; Caricato, M.; Li, X.; Hratchian, H. P.; Izmaylov, A. F.; Bloino, J.; Zheng, G.; Sonnenberg, J. L.; Hada, M.; Ehara, M.; Toyota, K.; Fukuda, R.; Hasegawa, J.; Ishida, M.; Nakajima, T.; Honda, Y.; Kitao, O.; Nakai, H.; Vreven, T.; Montgomery, Jr., J. A.; Peralta, J. E.; Ogliaro, F.; Bearpark, M.; Heyd, J. J.; Brothers, E.; Kudin, K. N.; Staroverov, V. N.; Kobayashi, R.; Normand, J.; Raghavachari, K.; Rendell, A.; Burant, J. C.; Iyengar, S. S.; Tomasi, J.; Cossi, M.; Rega, N.; Millam, J. M.; Klene, M.; Knox, J. E.; Cross, J. B.; Bakken, V.; Adamo, C.; Jaramillo, J.; Gomperts, R.; Stratmann, R. E.; Yazyev, O.; Austin, A. J.; Cammi, R.; Pomelli, C.; Ochterski, J. W.; Martin, R. L.; Morokuma, K.; Zakrzewski, V. G.; Voth, G. A.; Salvador, P.; Dannenberg, J. J.; Dapprich, S.; Daniels, A. D.; Farkas, Ö.; Foresman, J. B.; Ortiz, J. V.; Cioslowski, J.; Fox, D. J.Gaussian 09, Rev. C 01; Gaussian, Inc., Wallingford CT, 2009.

(3) Stephens, P. J.; Harada, N. ECD cotton effect approximated by the Gaussian curve and other methods. Chirality. 2010, 22, 229–233

Table S8. Conformational analysis of **5Ja**.

No	conformer	population
5Ja-C1	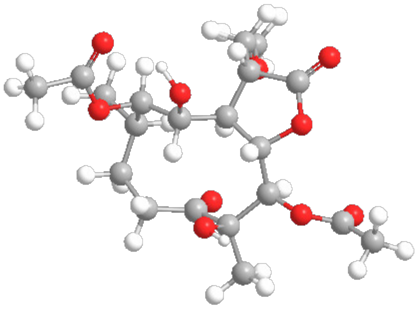	85.29%
5Ja-C2	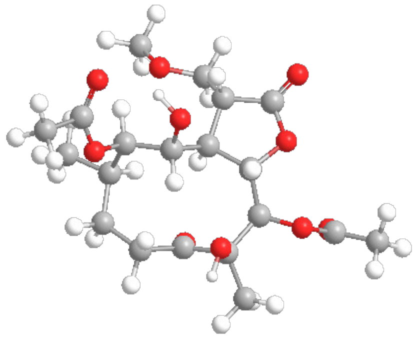	3.92%
5Ja-C3	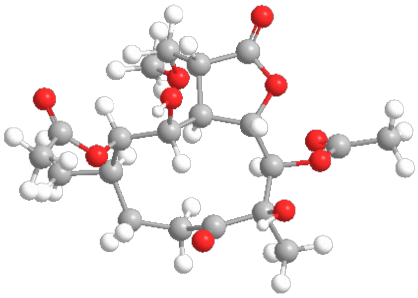	3.11%
5Ja-C4	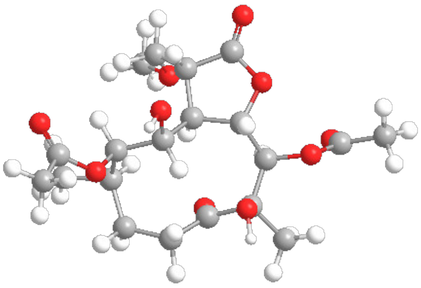	2.67%
5Ja-C5	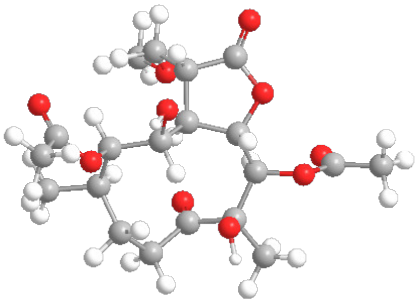	2.54%

Table S9. Conformational Analysis of **7Ja**.

No	conformer	population
7Ja-C1	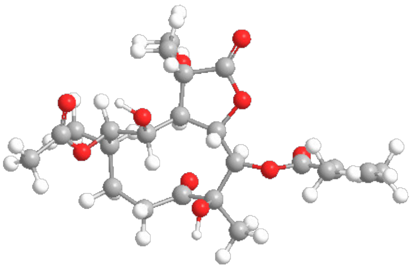	24.48%
7Ja-C2	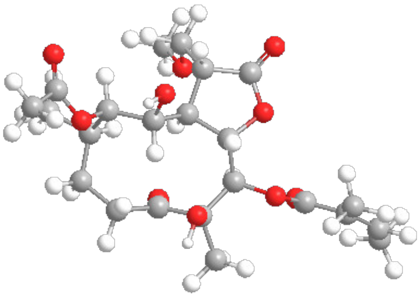	21.67%
7Ja-C3	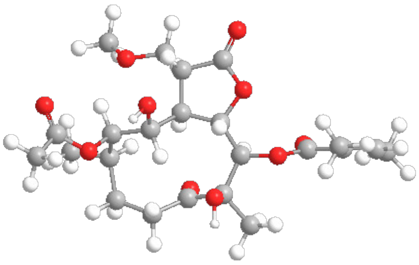	21.47%
7Ja-C4	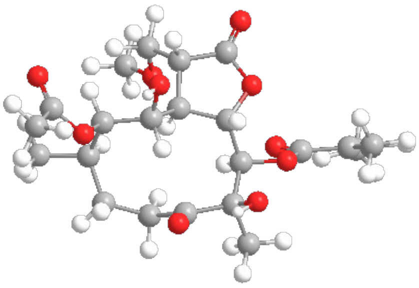	8.63%
7Ja-C5	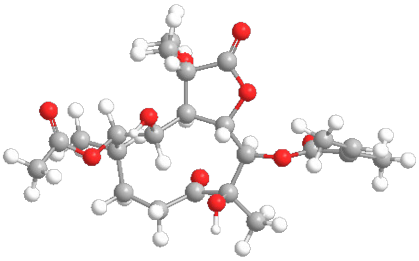	6.95%
7Ja-C6	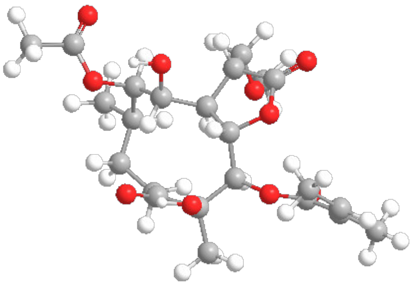	5.88%

7Ja-C7	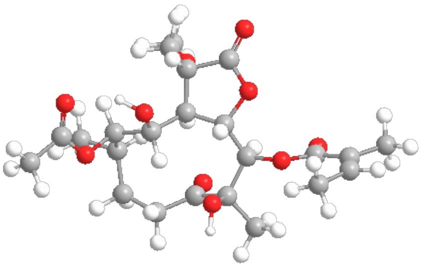	5.62%
7Ja-C8	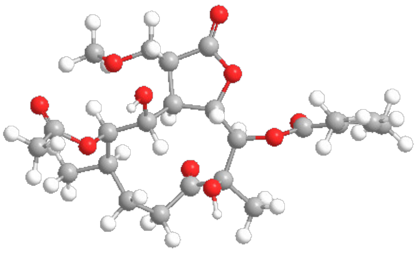	2.78%
7Ja-C9	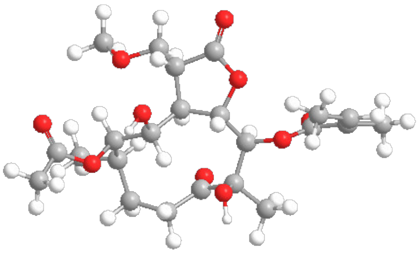	1.24%

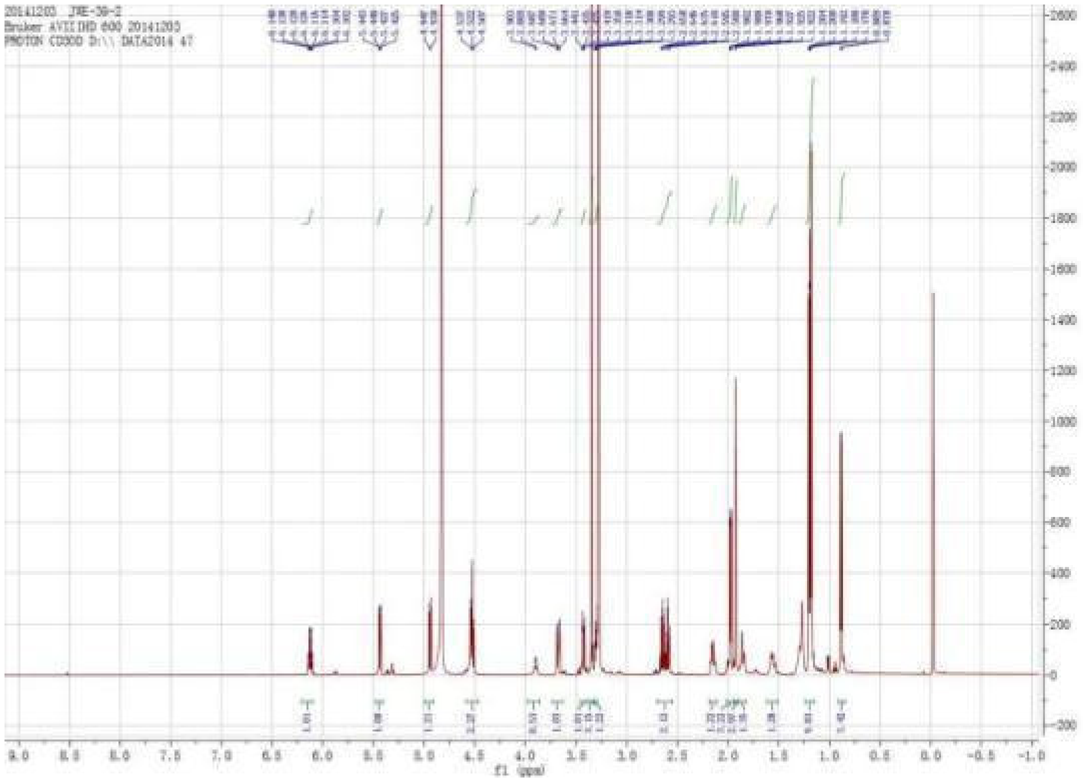

Figure S1.1 ^{1}H NMR spectrum of compound **1** in CD_3OD.

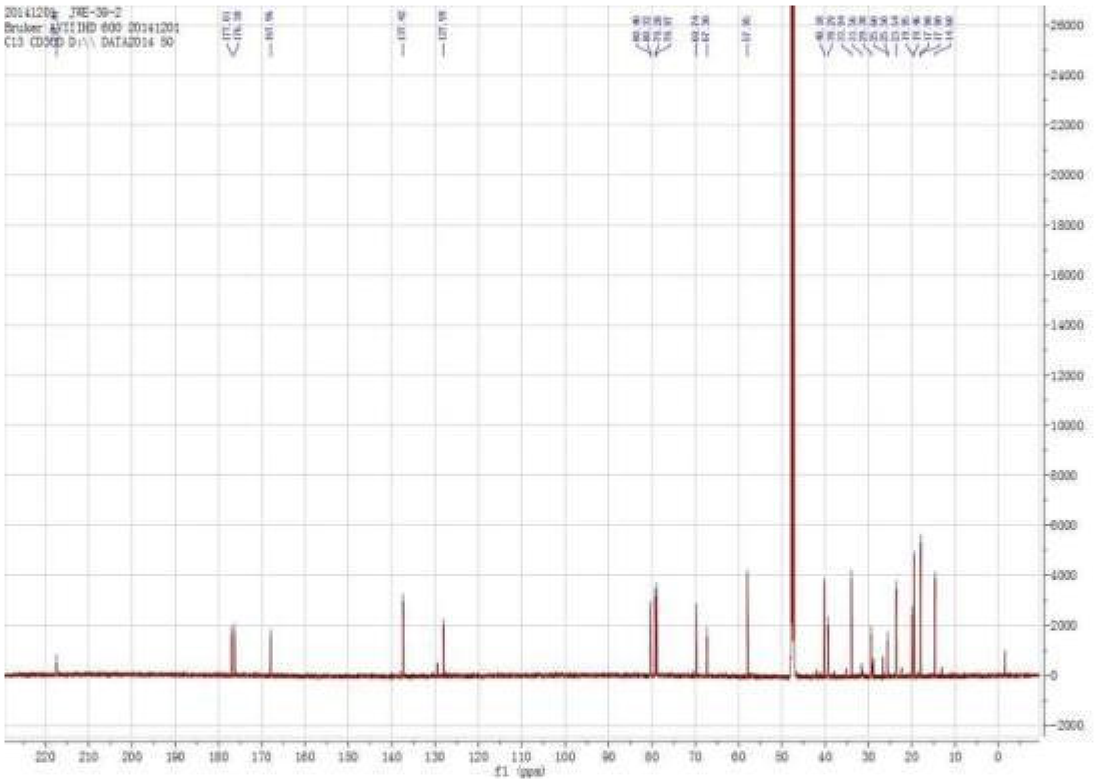

Figure S1.2 ^{13}C NMR spectrum of compound **1** in CD_3OD.

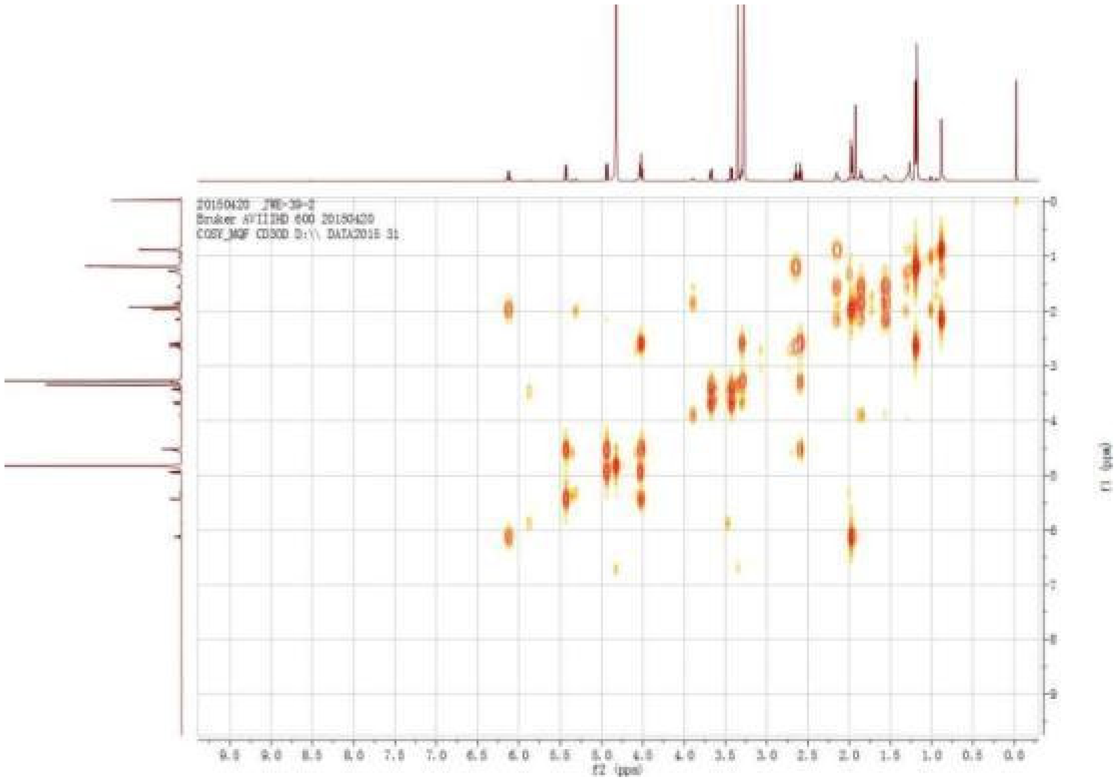

Figure S1.3 ^{1}H-^{1}H COSY spectrum of compound **1** in CD_3OD.

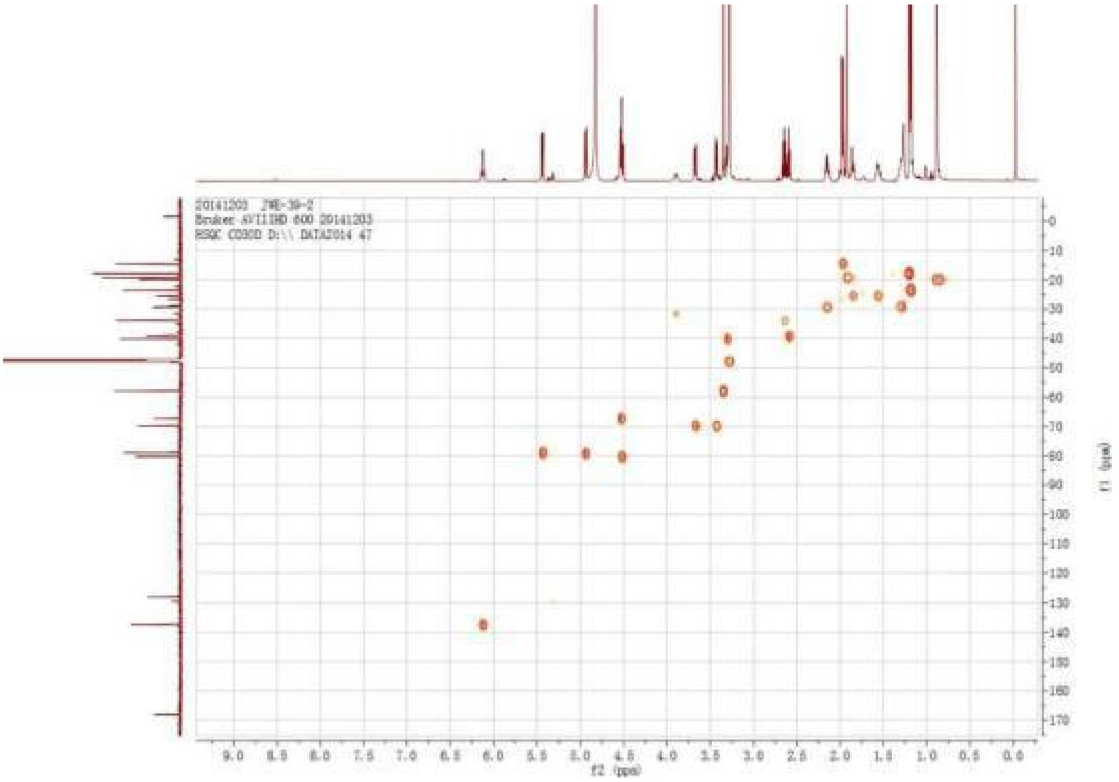

Figure S1.4 HSQC spectrum of compound **1** in CD_3OD.

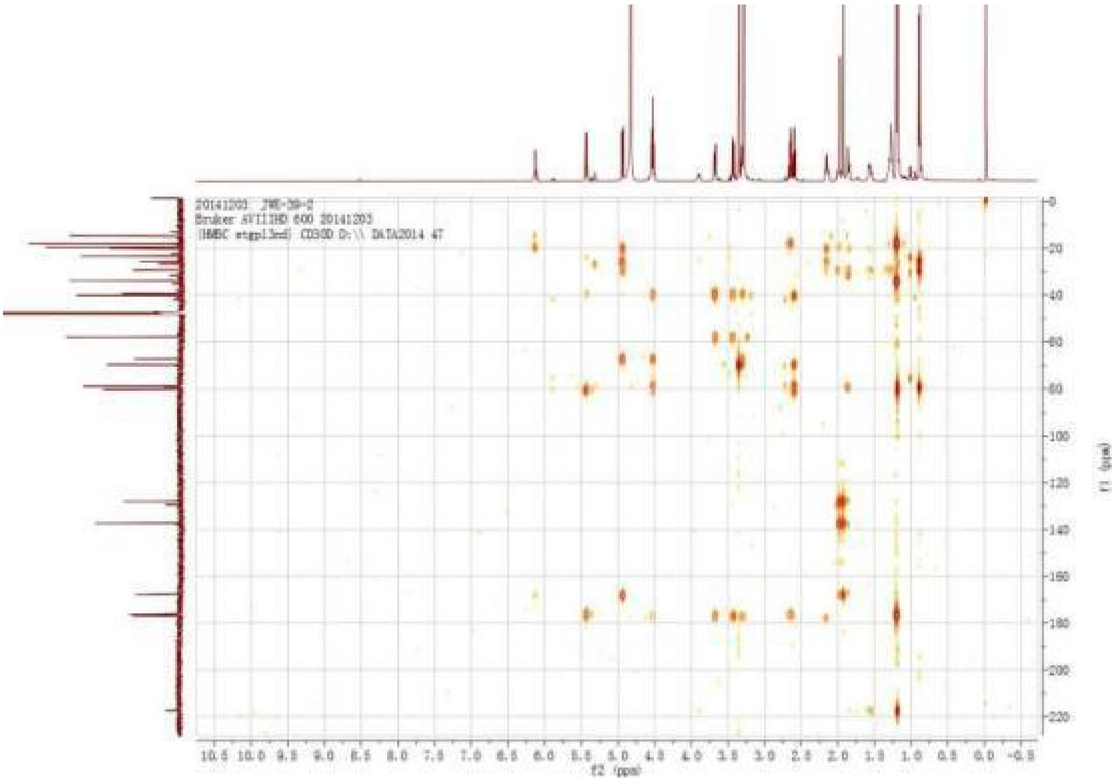

Figure S1.5 HMBC spectrum of compound **1** in CD_3OD.

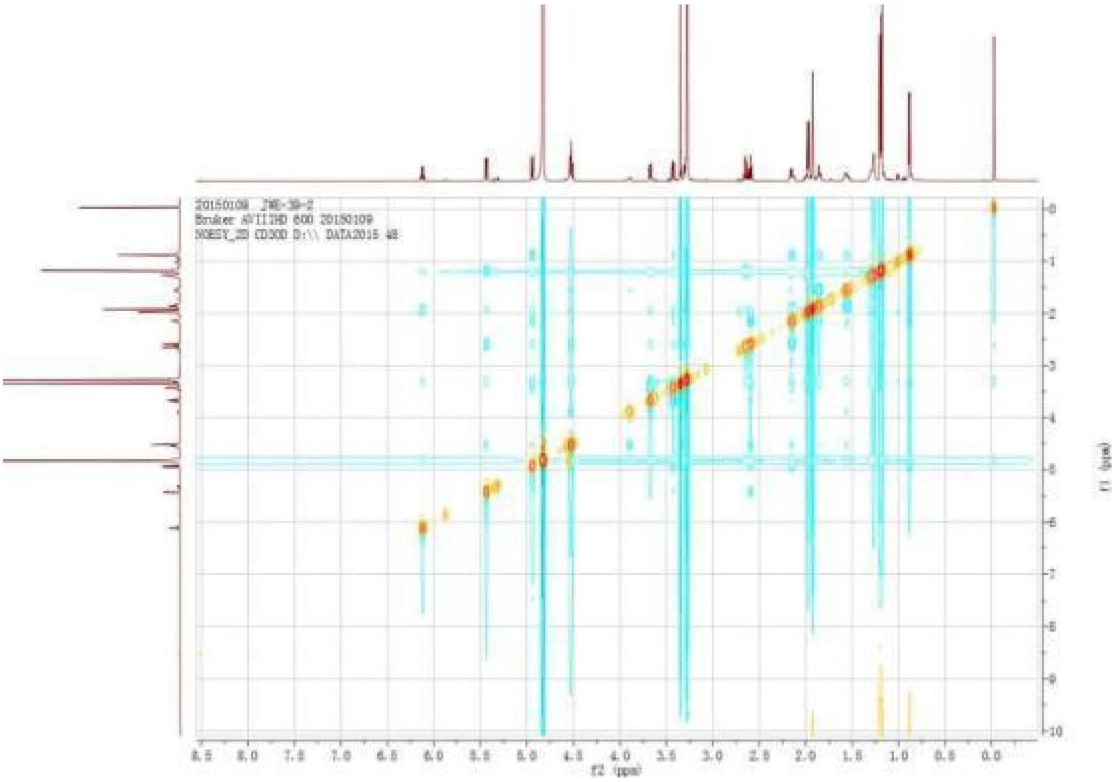

Figure S1.6 NOESY spectrum of compound **1** in CD_3OD.

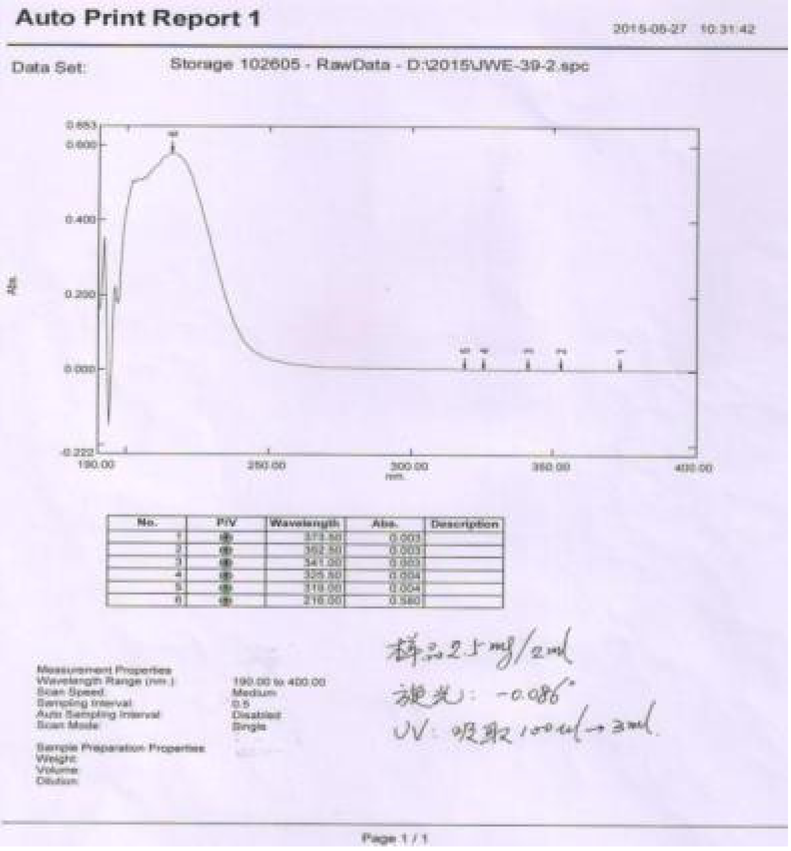

Figure S1.7 UV spectrum of compound **1**.

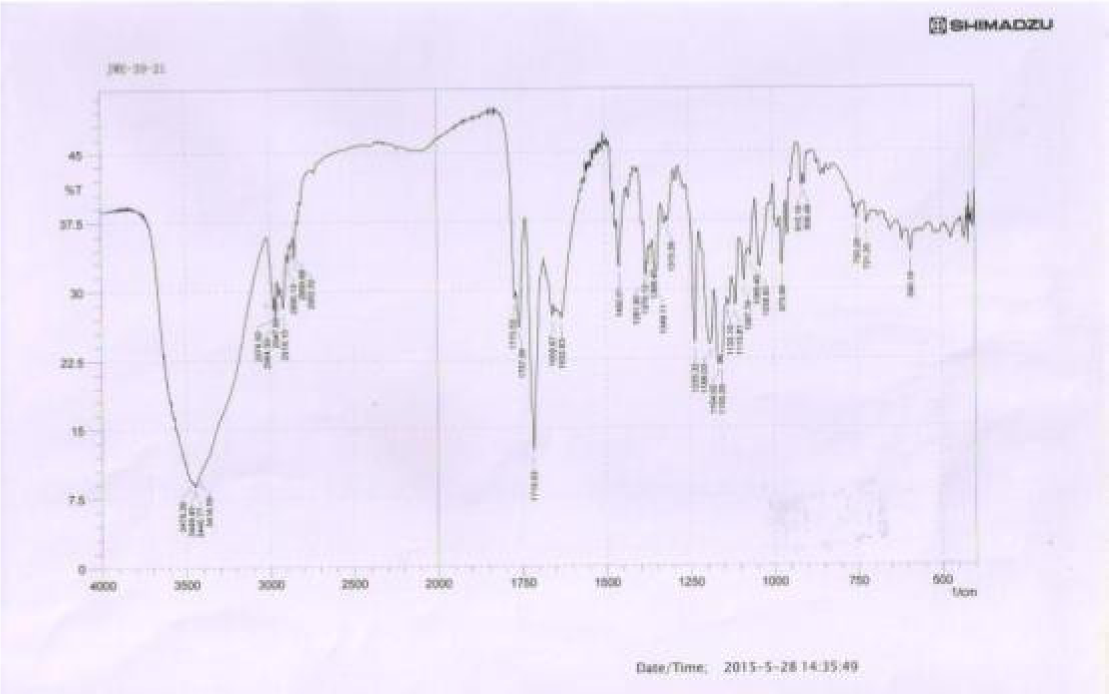

Figure S1.8 IR spectrum of compound **1**.

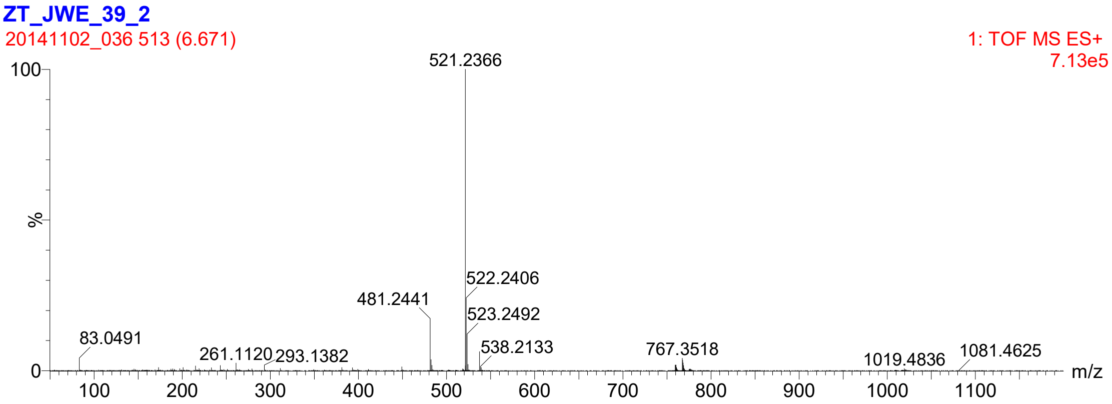

Figure S1.9 HRESIMS spectrum of compound **1**.

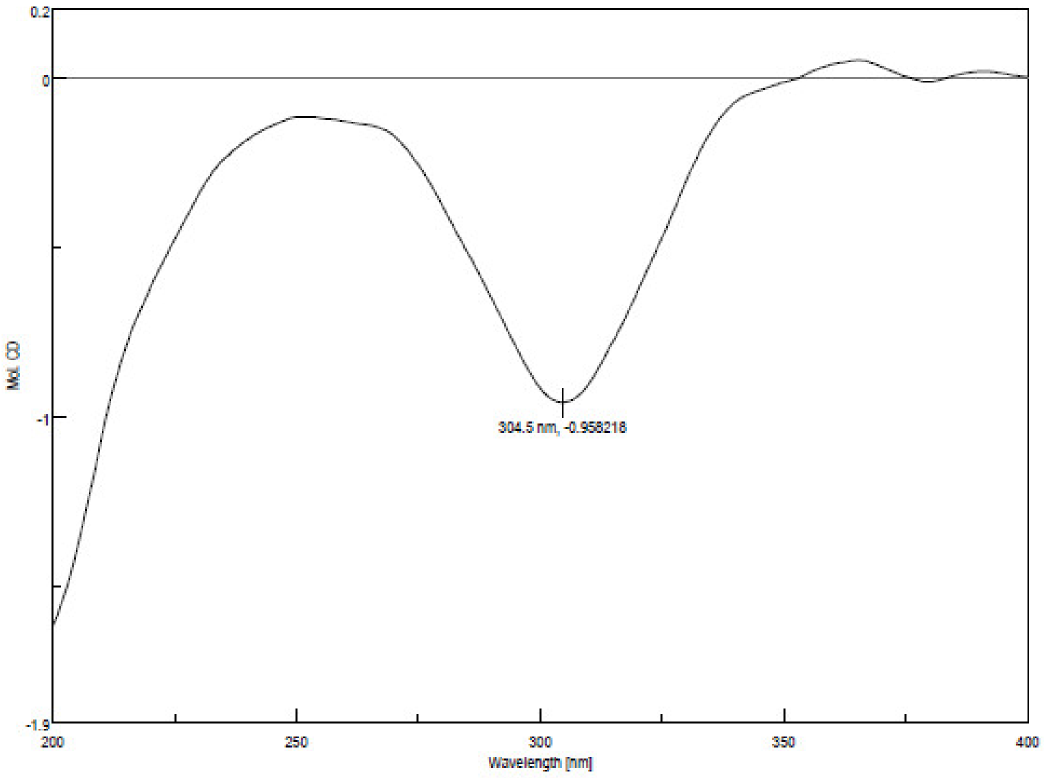

Figure S1.10 ECD spectrum of compound **1**.

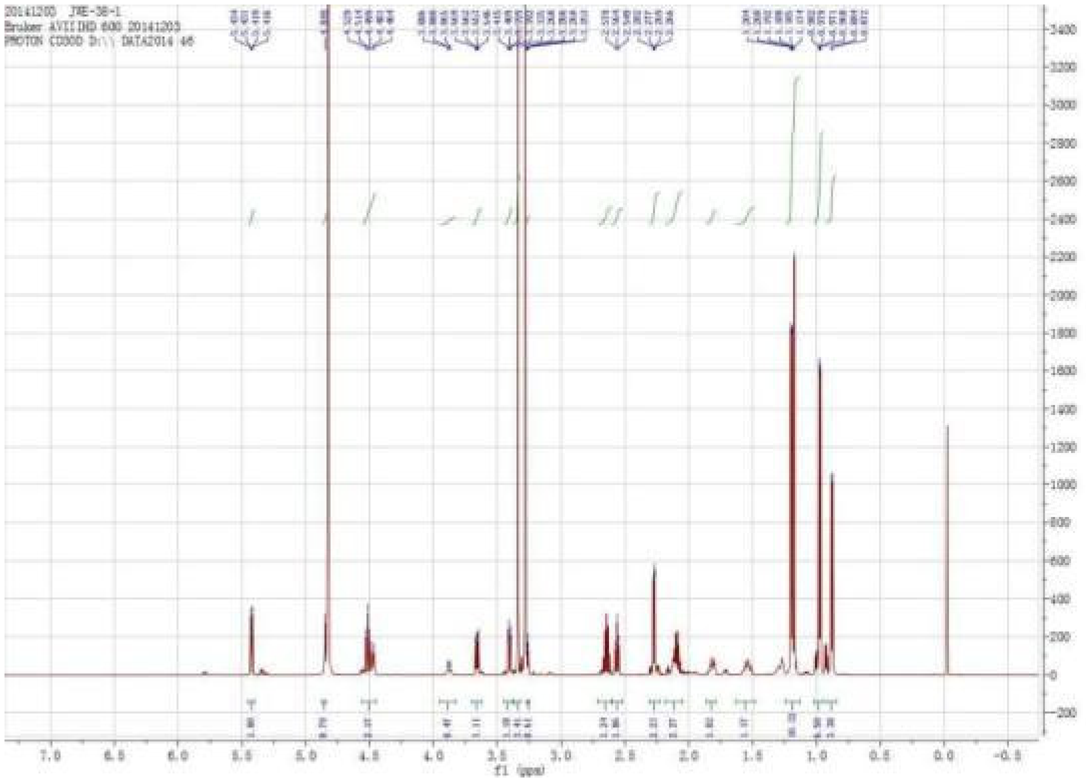

Figure S2.1 ^{1}H NMR spectrum of compound **2** in CD_3OD.

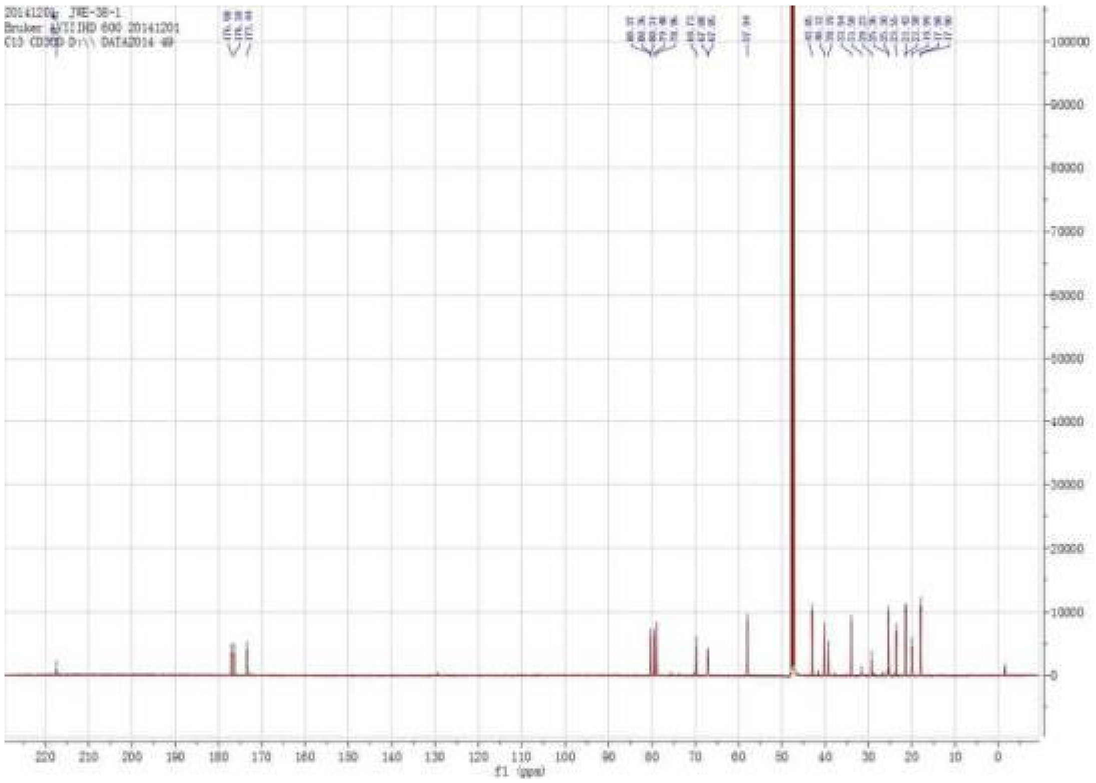

Figure S2.2 ^{13}C NMR spectrum of compound **2** in CD_3OD.

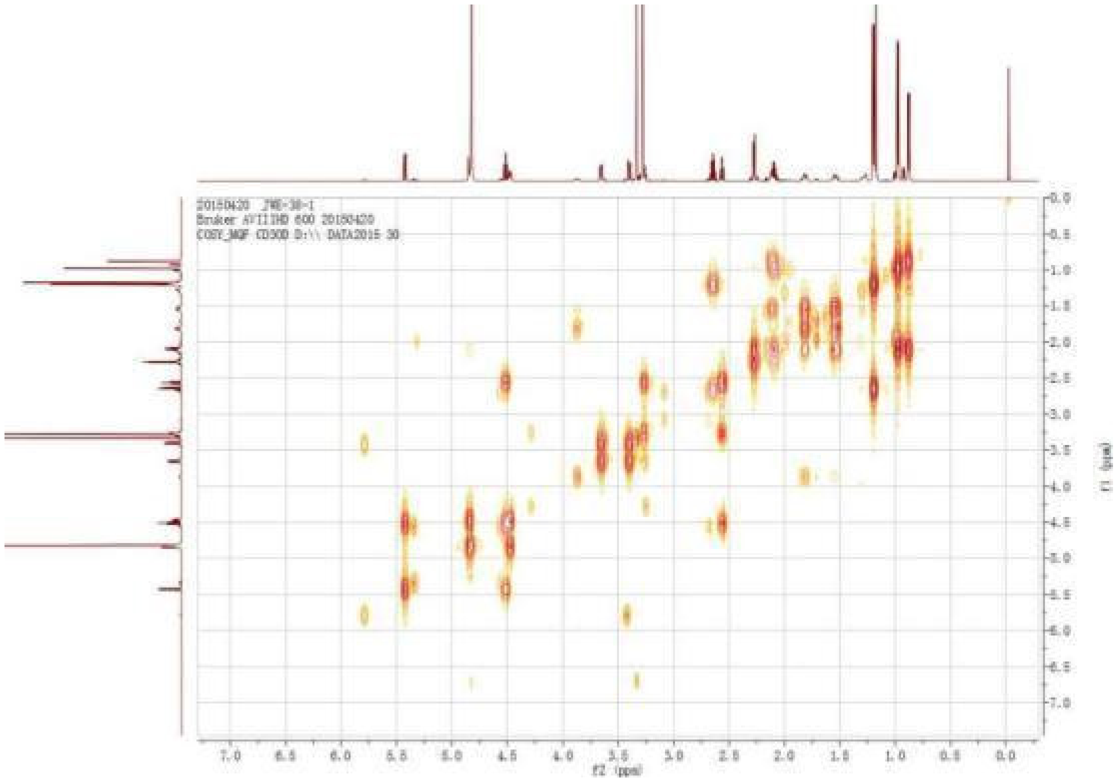

Figure S2.3 ^{1}H-^{1}H COSY spectrum of compound **2** in CD_3OD.

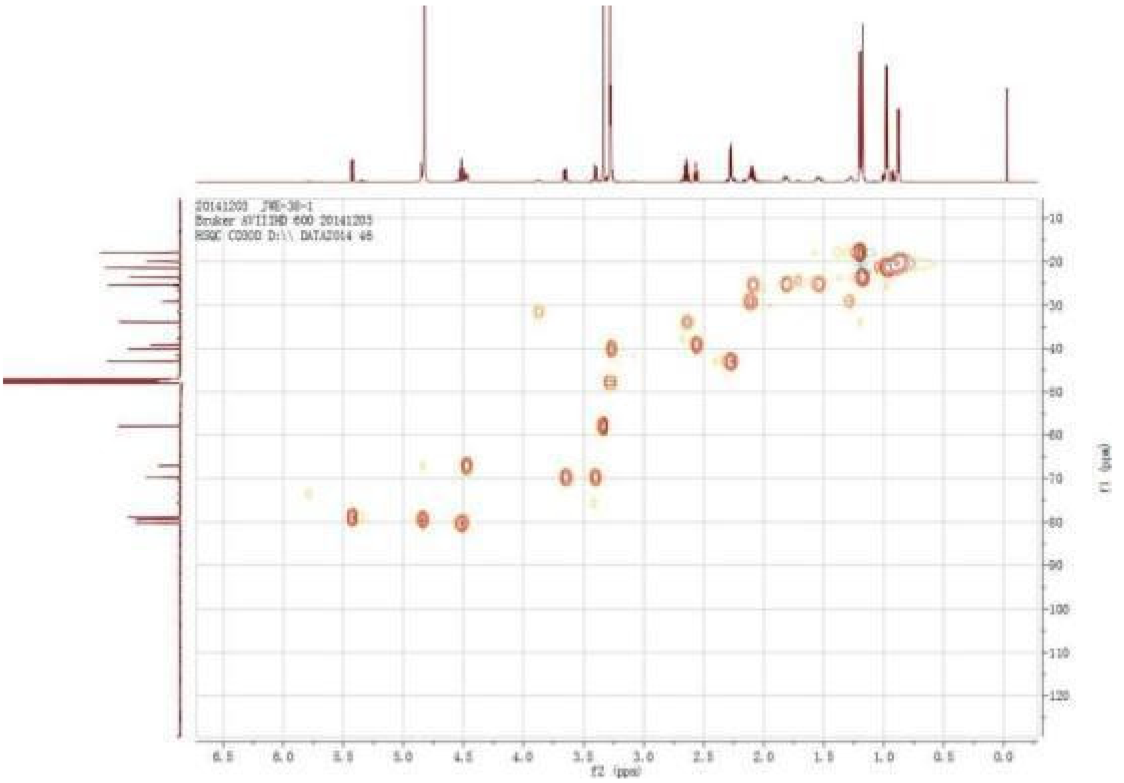

Figure S2.4 HSQC spectrum of compound **2** in CD_3OD.

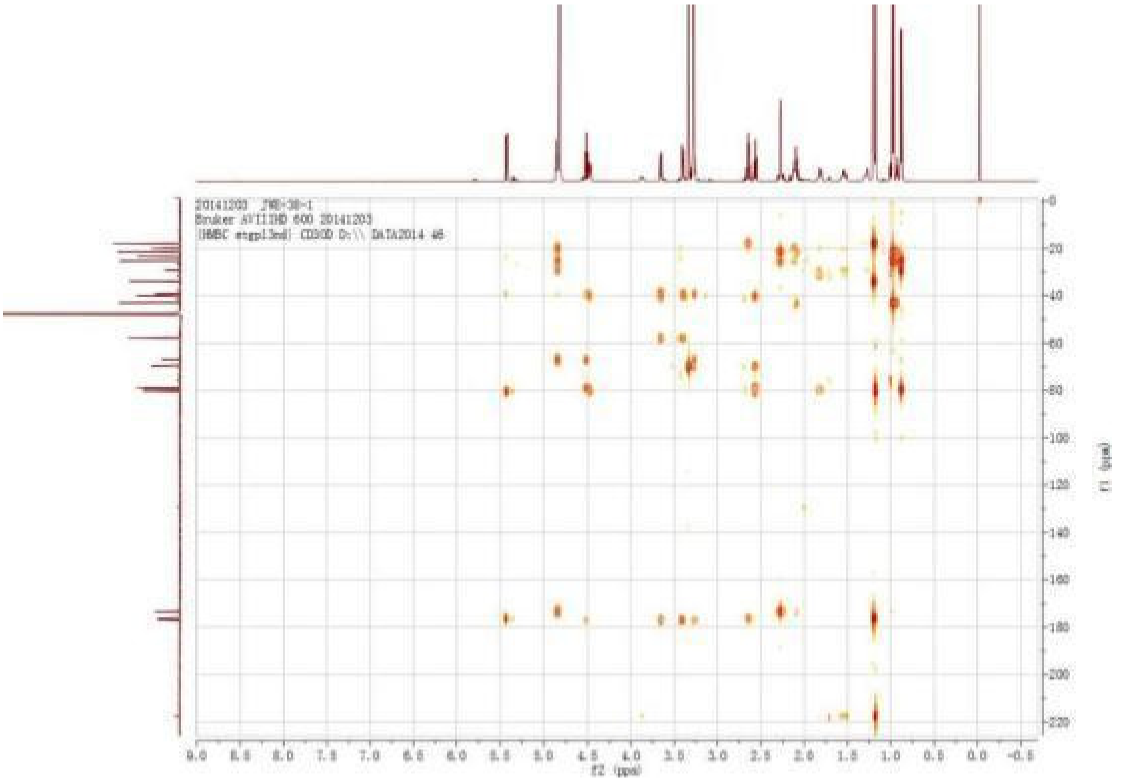

Figure S2.5 HMBC spectrum of compound **2** in CD_3OD.

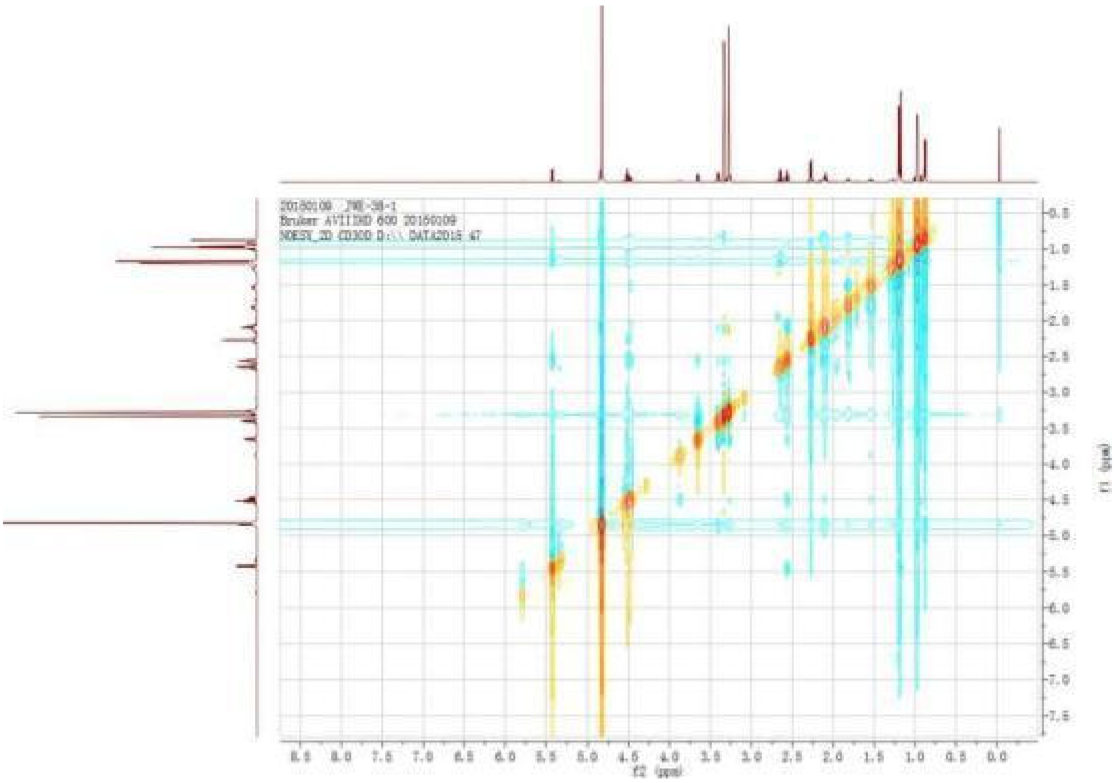

Figure S2.6 NOESY spectrum of compound **2** in CD_3OD.

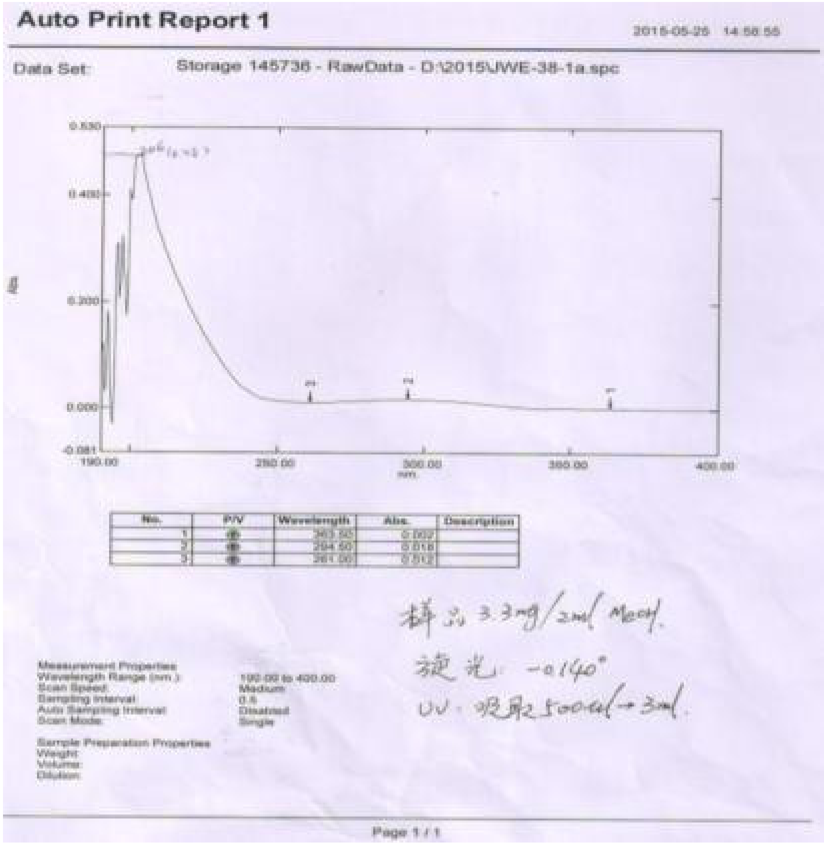

Figure S2.7 UV spectrum of compound **2**.

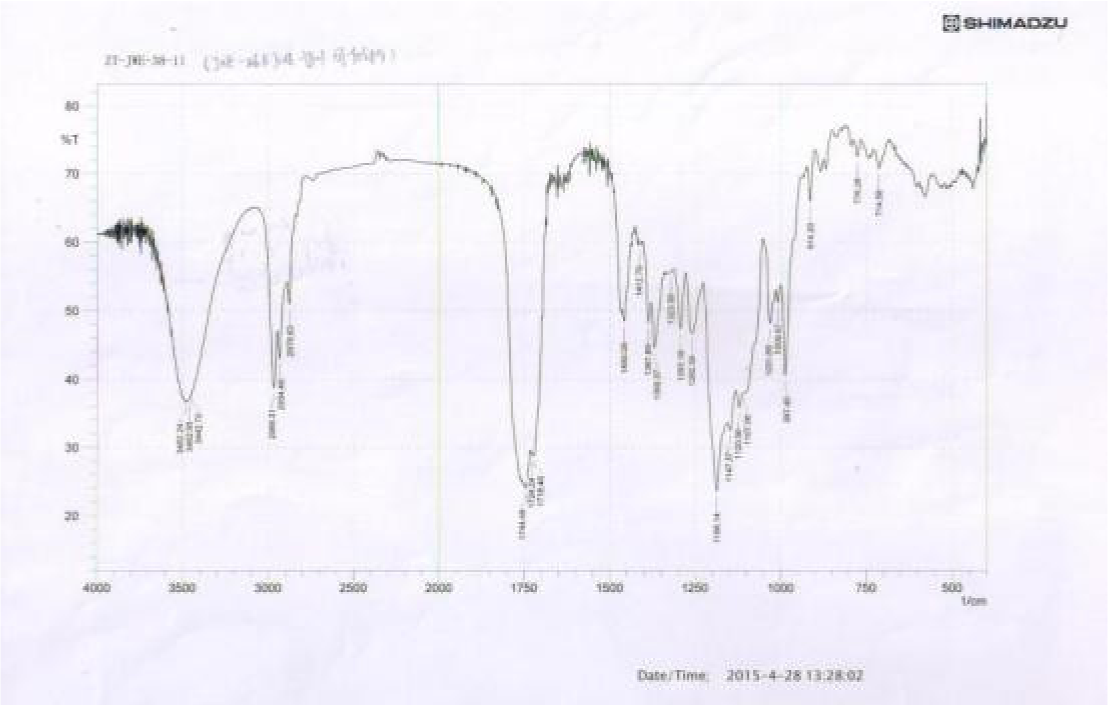

Figure S2.8 IR spectrum of compound **2**.

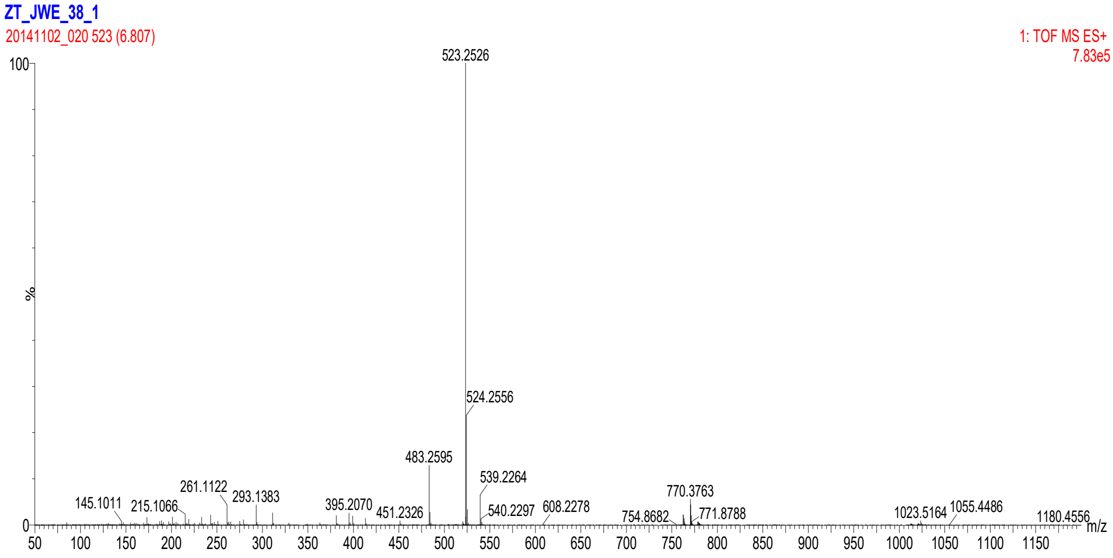

Figure S2.9 HRESIMS spectrum of compound **2**.

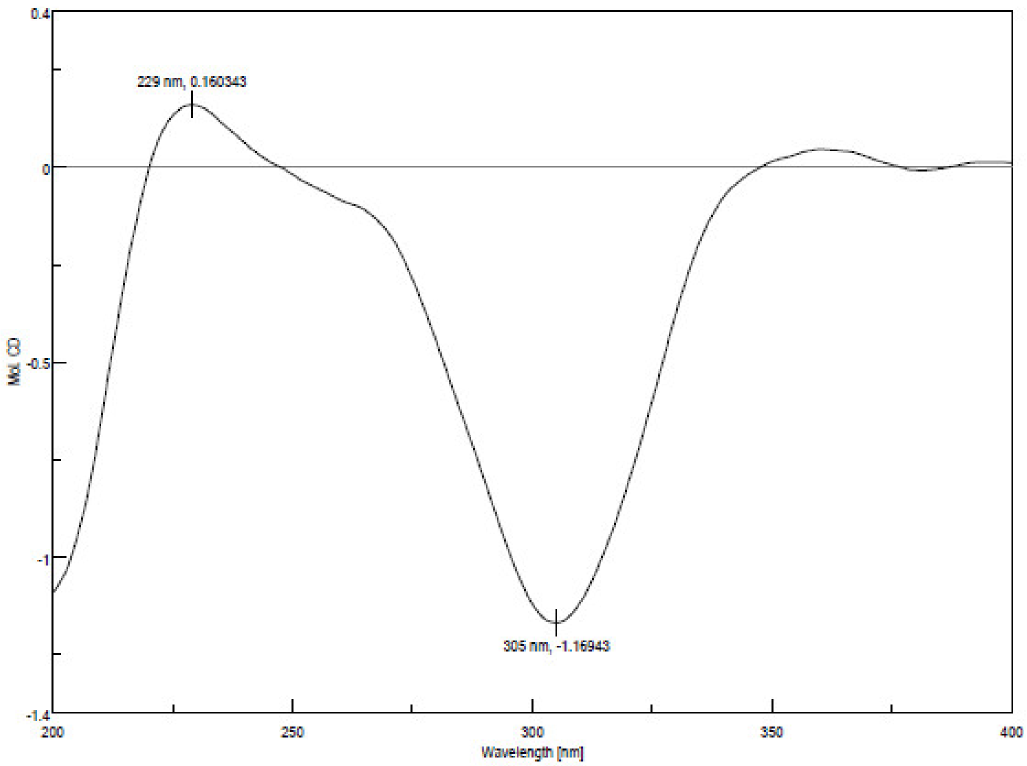

Figure S2.10 ECD spectrum of compound **2**.

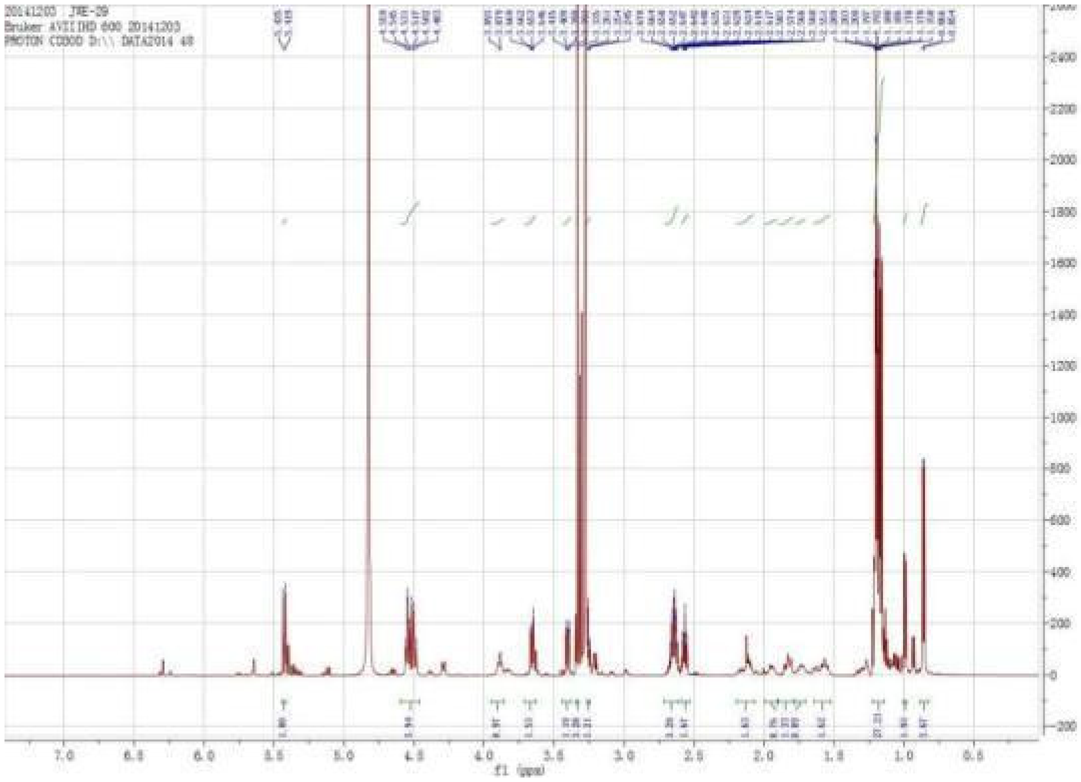

Figure S3.1 ^{1}H NMR spectrum of compound **3** in CD_3OD.

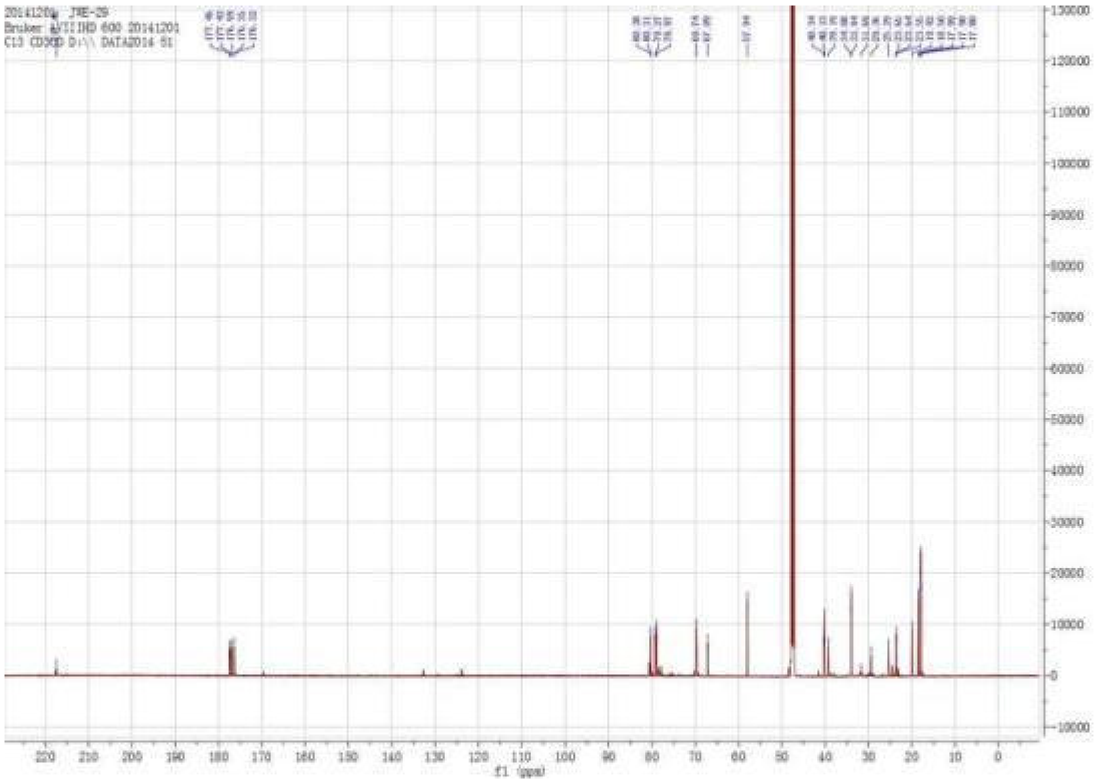

Figure S3.2 ^{13}C NMR spectrum of compound **3** in CD_3OD.

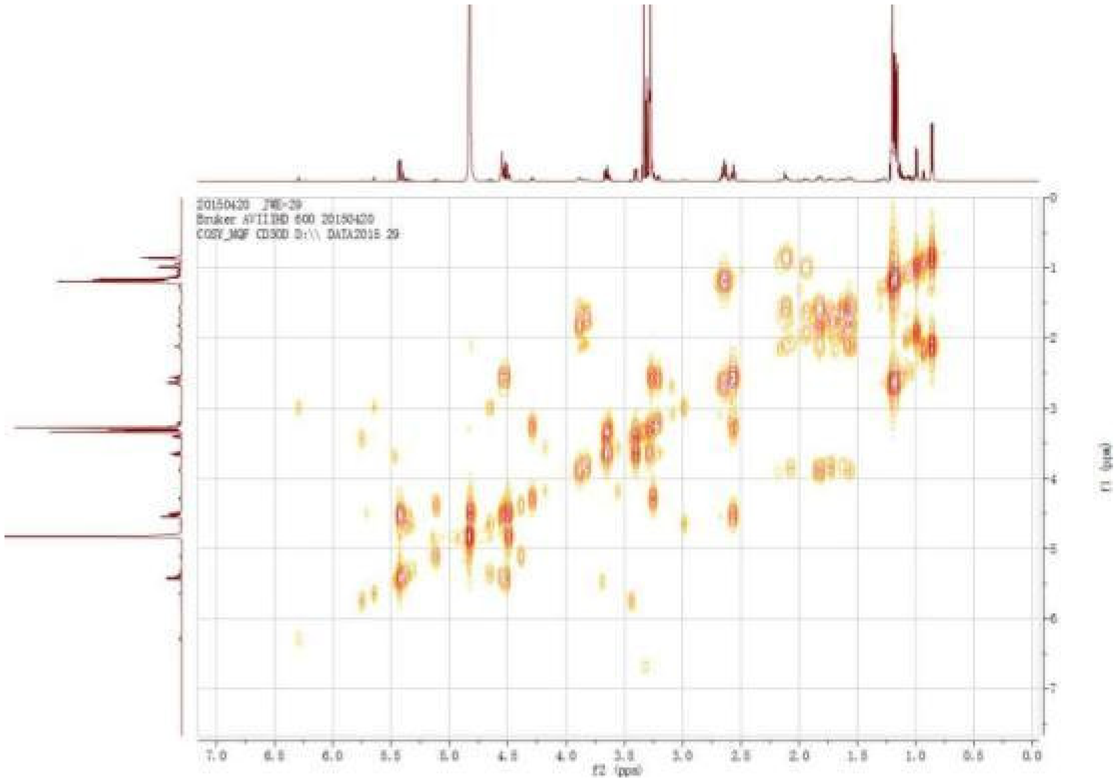

Figure S3.3 ^{1}H-^{1}H COSY spectrum of compound **3** in CD_3OD.

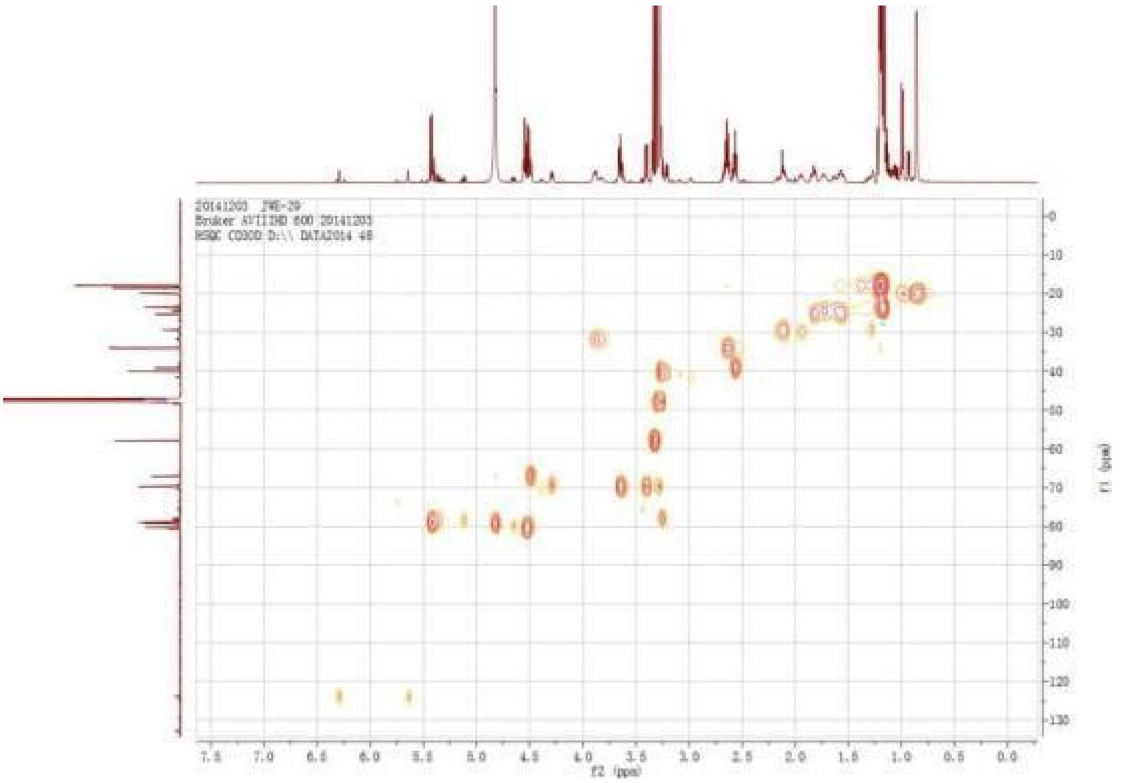

Figure S3.4 HSQC spectrum of compound **3** in CD_3OD.

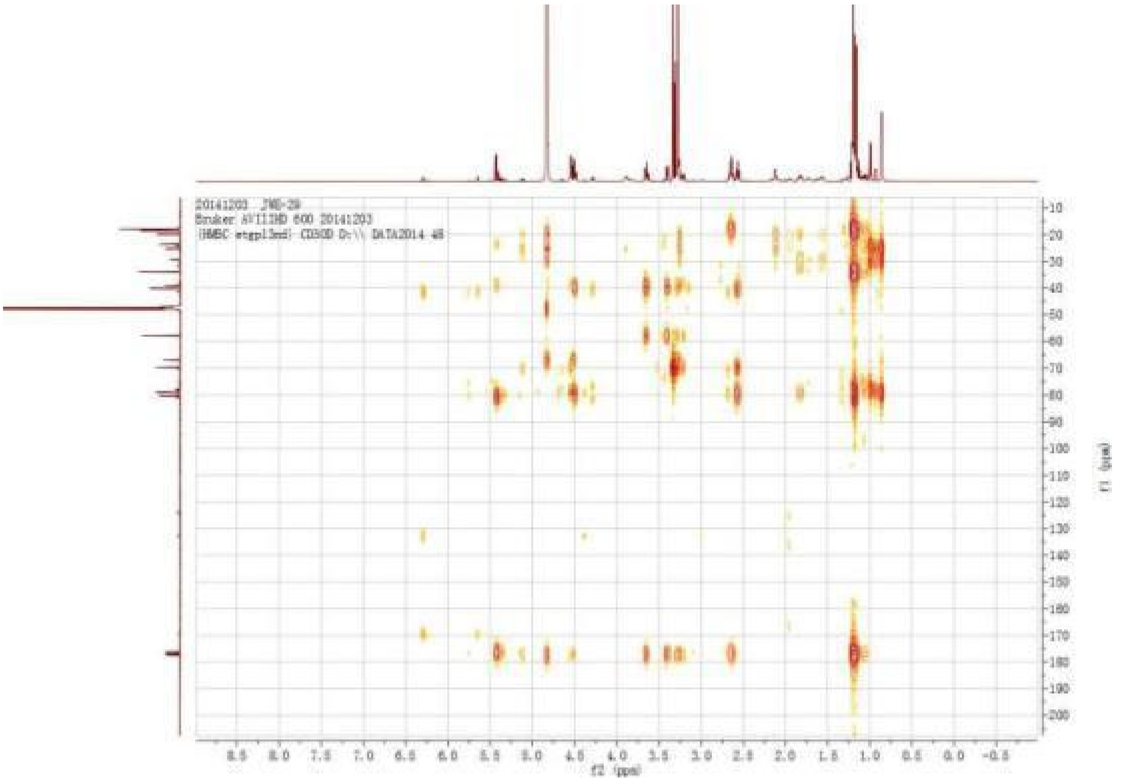

Figure S3.5 HMBC spectrum of compound **3** in CD_3OD.

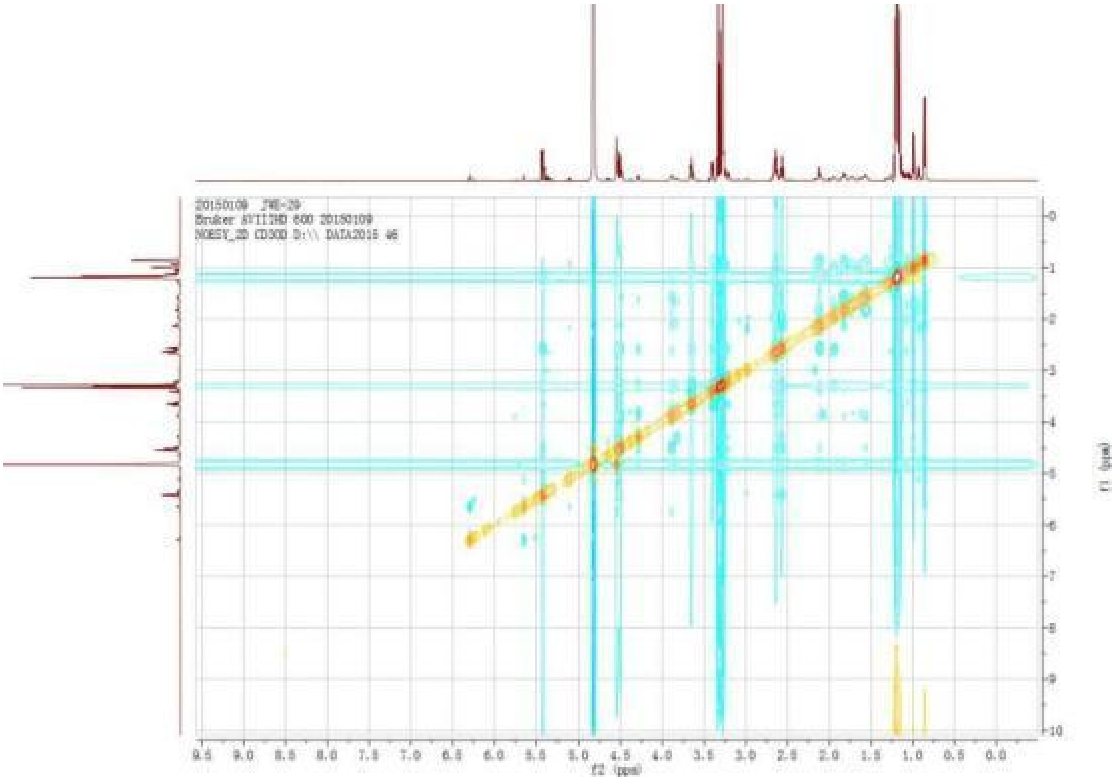

Figure S3.6 NOESY spectrum of compound **3** in CD_3OD.

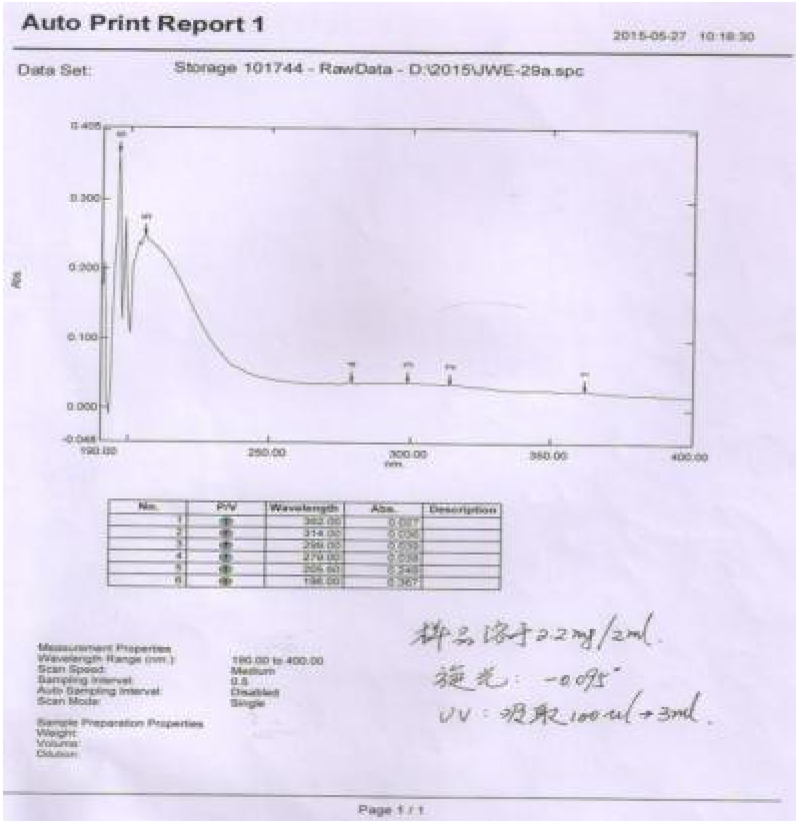

Figure S3.7 UV spectrum of compound **3**.

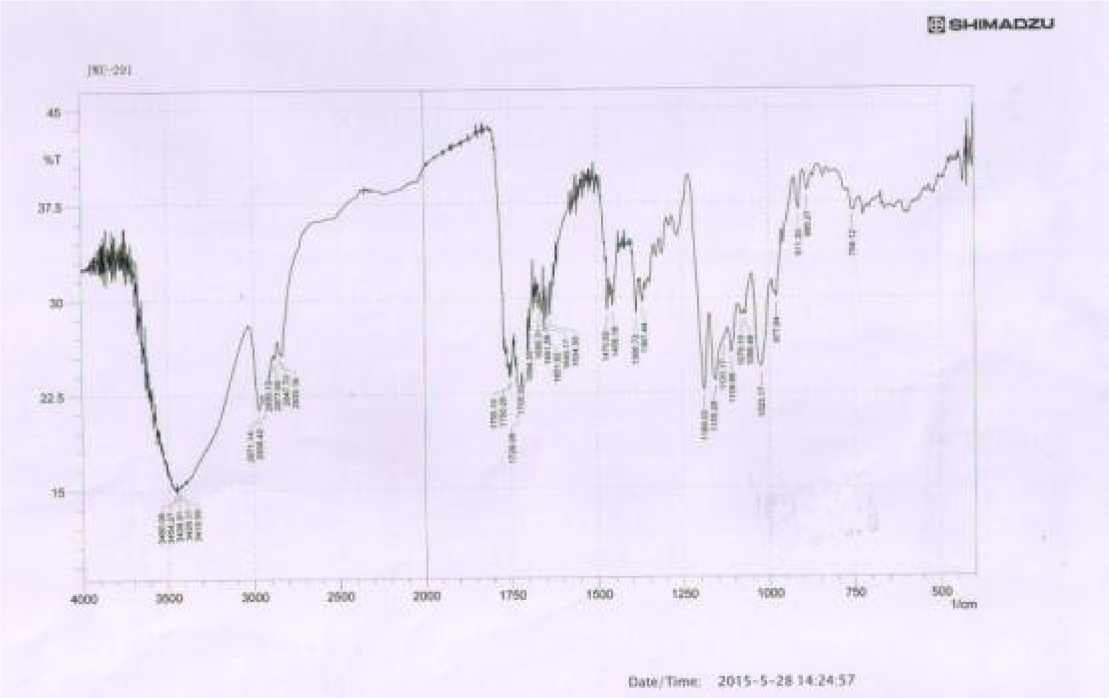

Figure S3.8 IR spectrum of compound **3**.

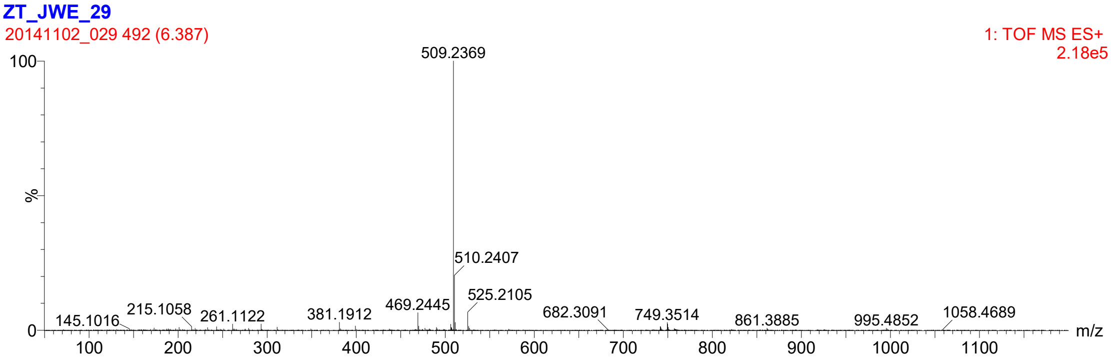

Figure S3.9 HRESIMS spectrum of compound **3**.

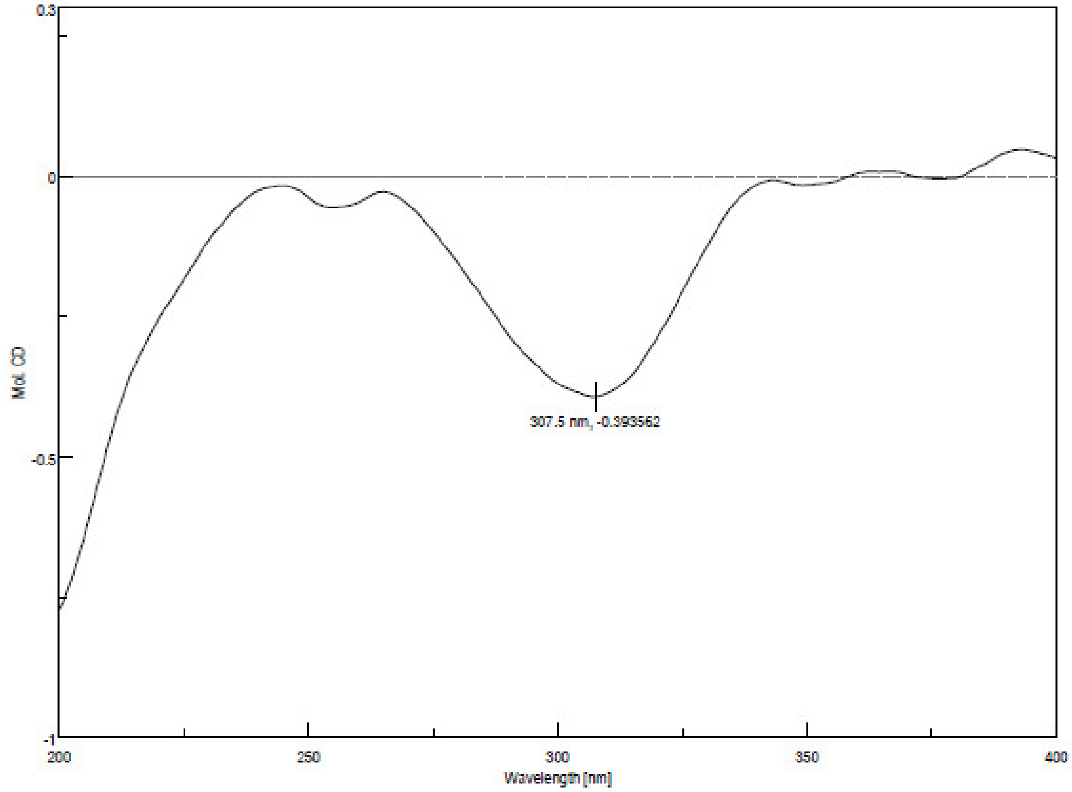

Figure S3.10 ECD spectrum of compound **3**.

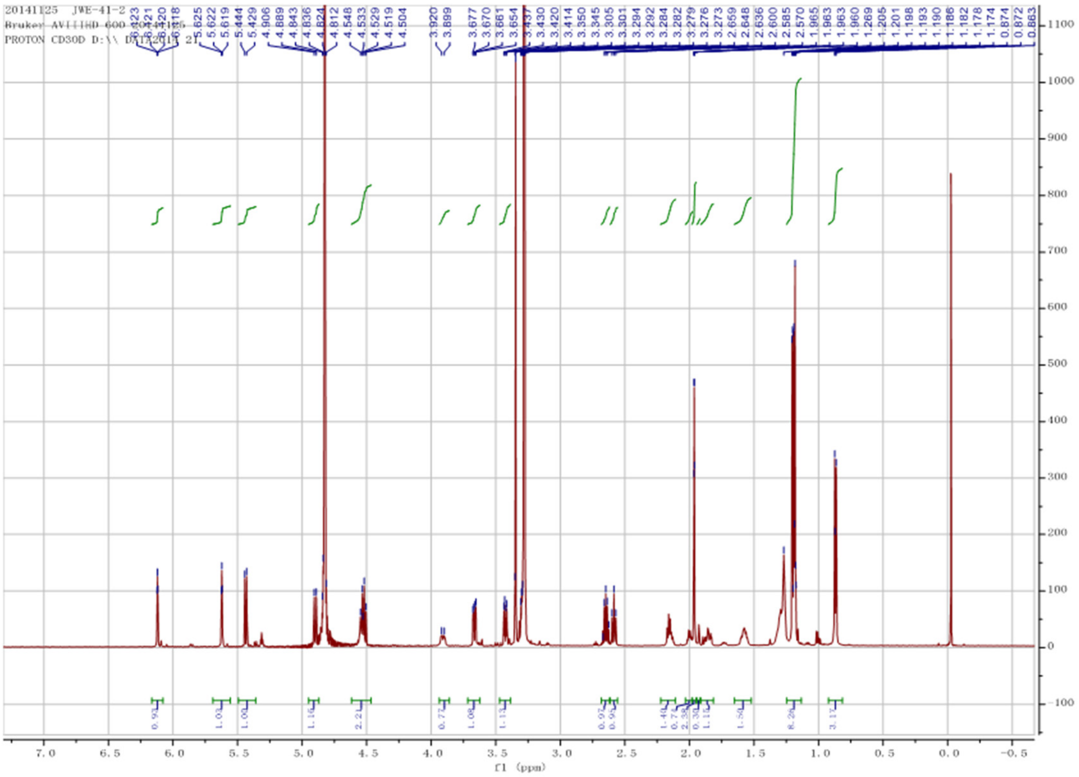

Figure S4.1 ^{1}H NMR spectrum of compound **4** in CD_3OD.

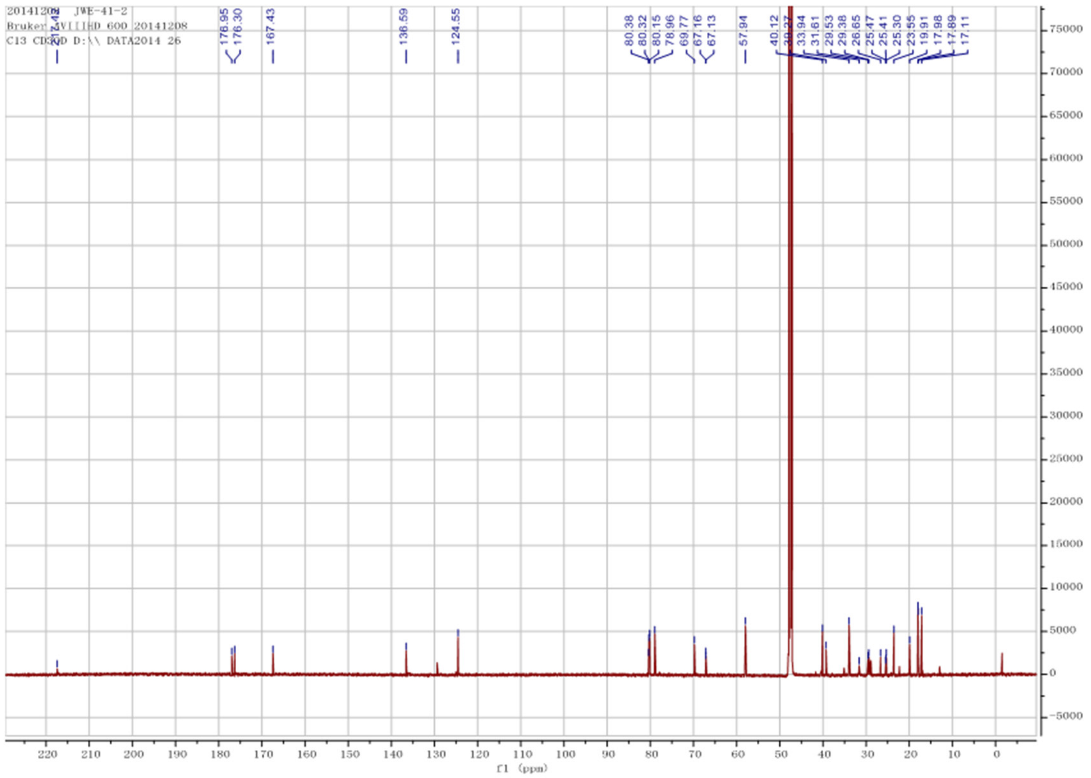

Figure S4.2 ^{13}C NMR spectrum of compound **4** in CD_3OD.

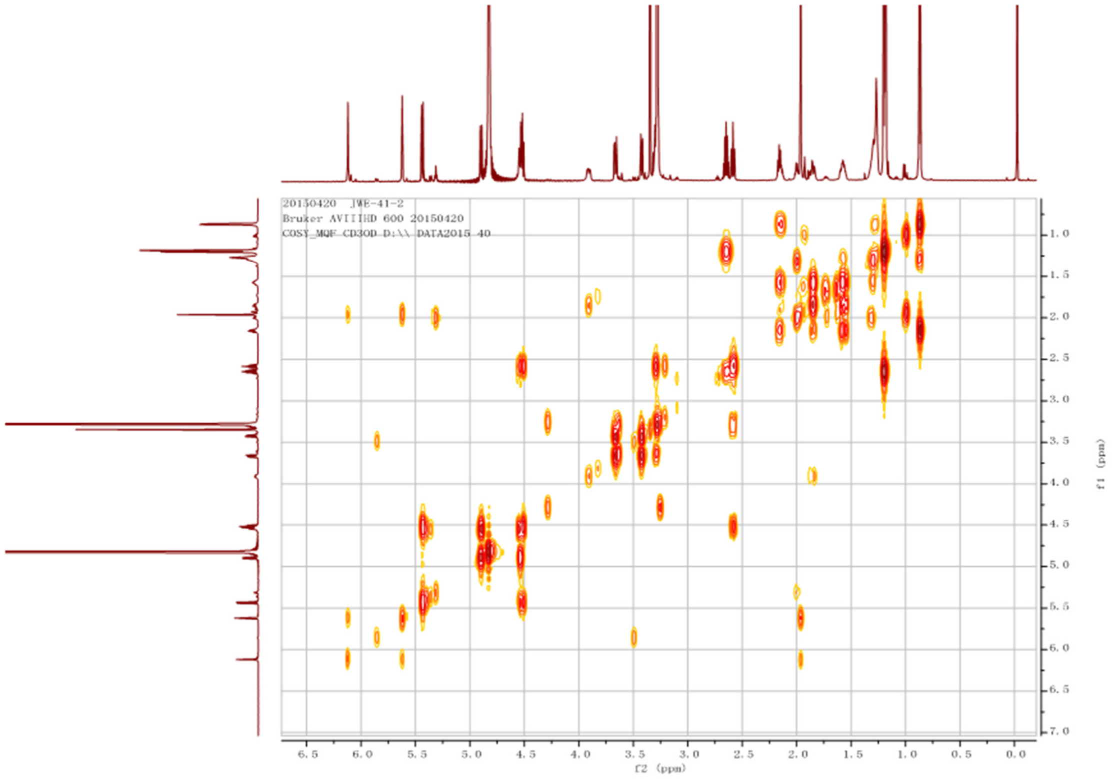

Figure S4.3 ^1H-^1H COSY spectrum of compound **4** in CD_3OD.

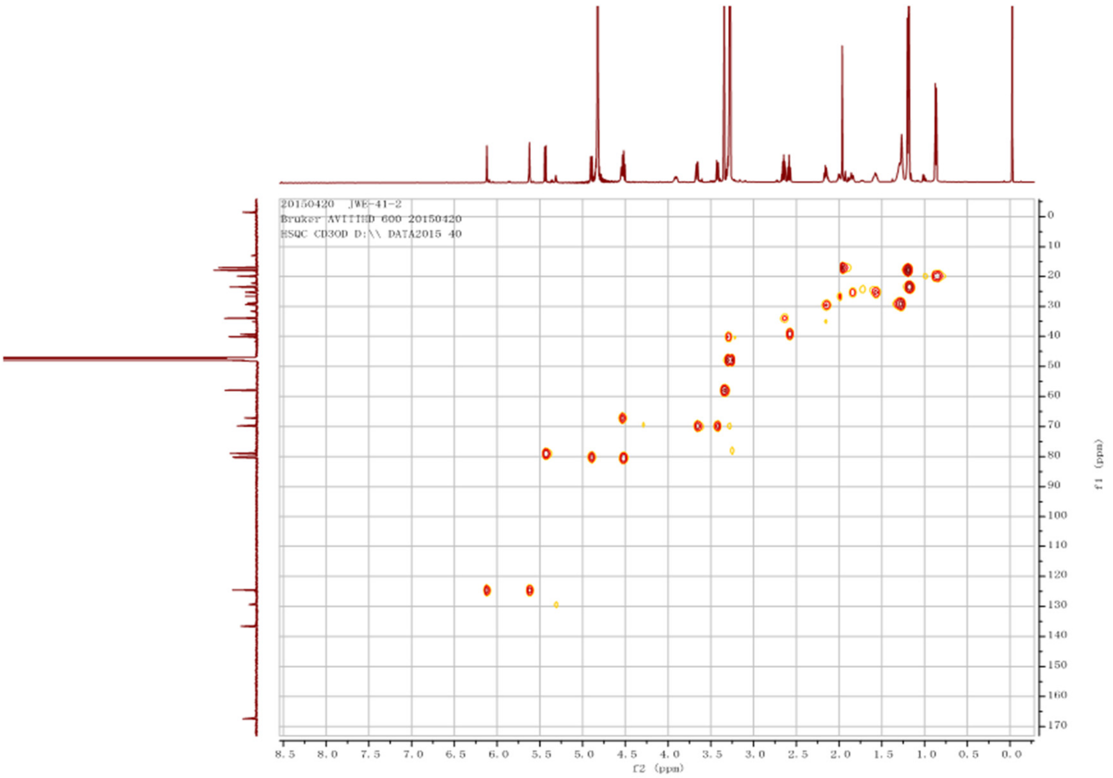

Figure S4.4 HSQC spectrum of compound **4** in CD_3OD.

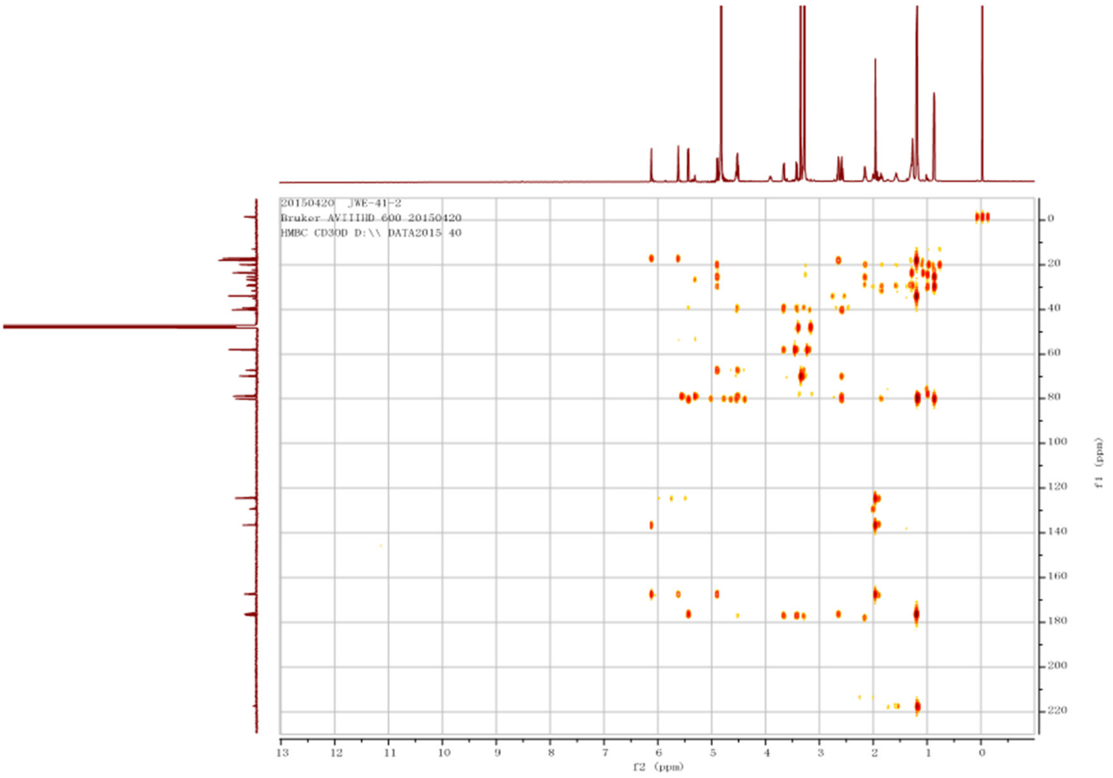

Figure S4.5 HMBC spectrum of compound **4** in CD_3OD.

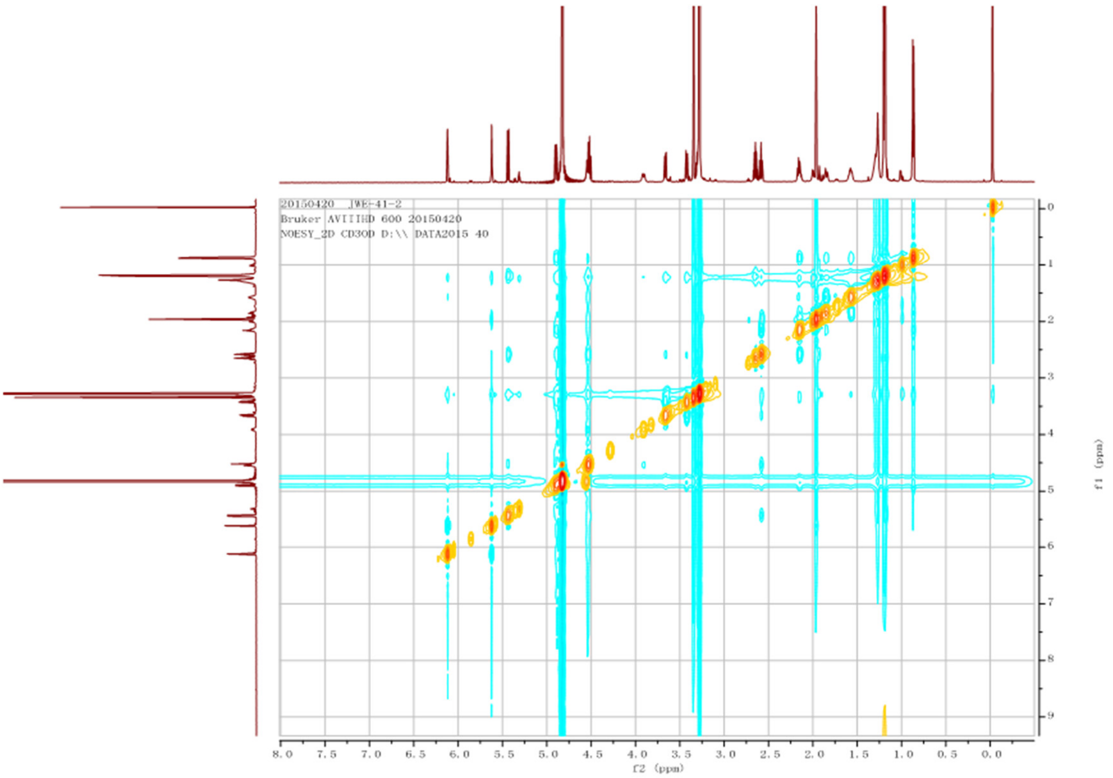

Figure S4.6 NOESY spectrum of compound **4** in CD_3OD.

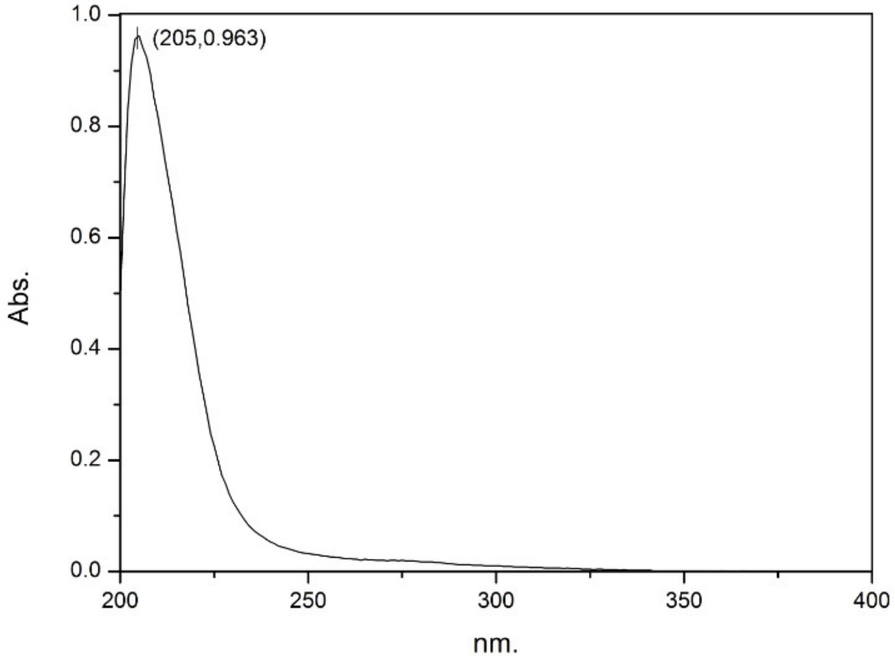

Figure S4.7 UV spectrum of compound **4**.

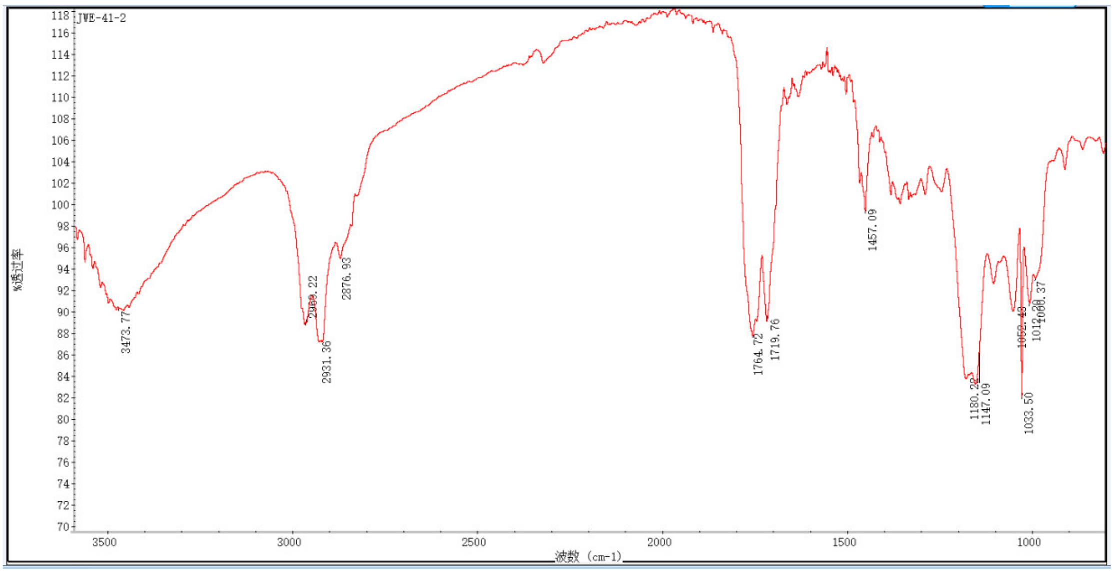

Figure S4.8 IR spectrum of compound **4**.

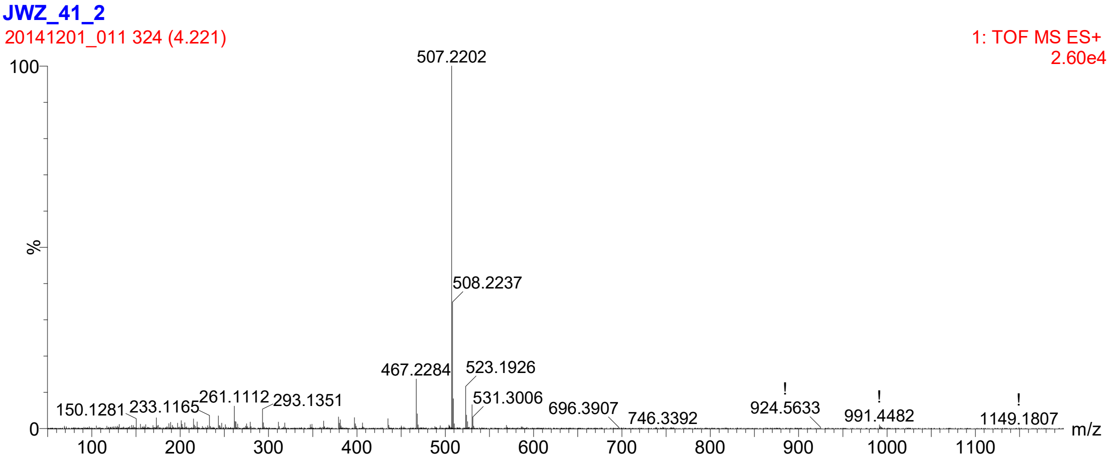

Figure S4.9 HRESIMS spectrum of compound **4**.

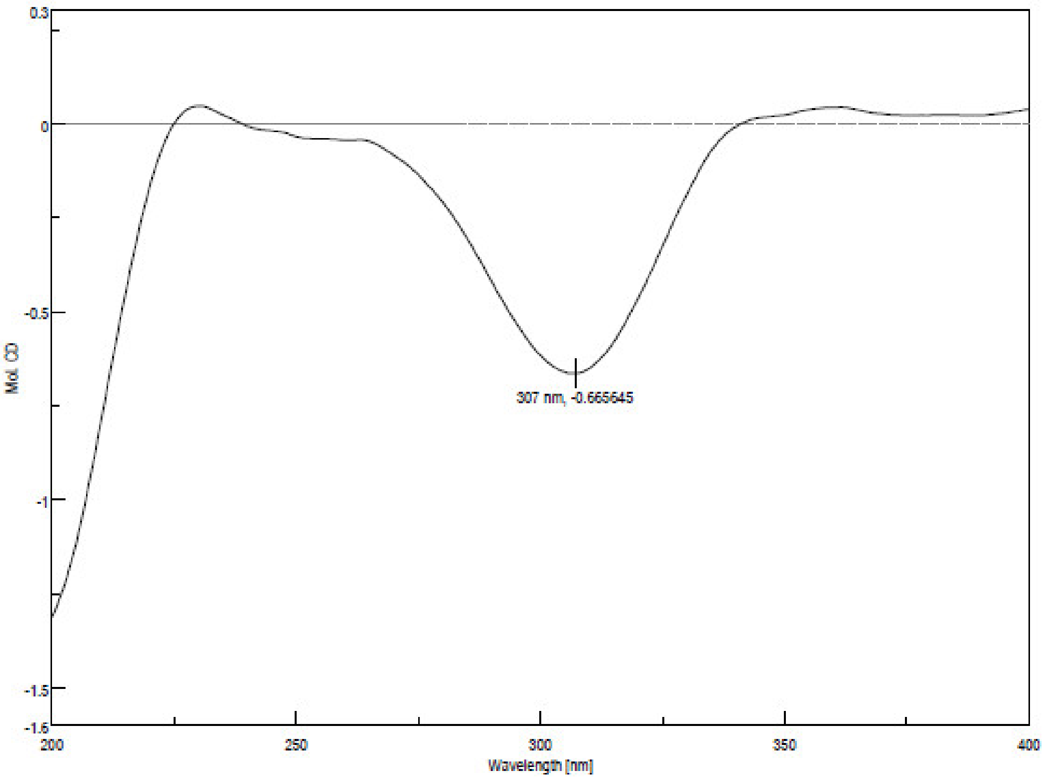

Figure S4.10 ECD spectrum of compound **4**.

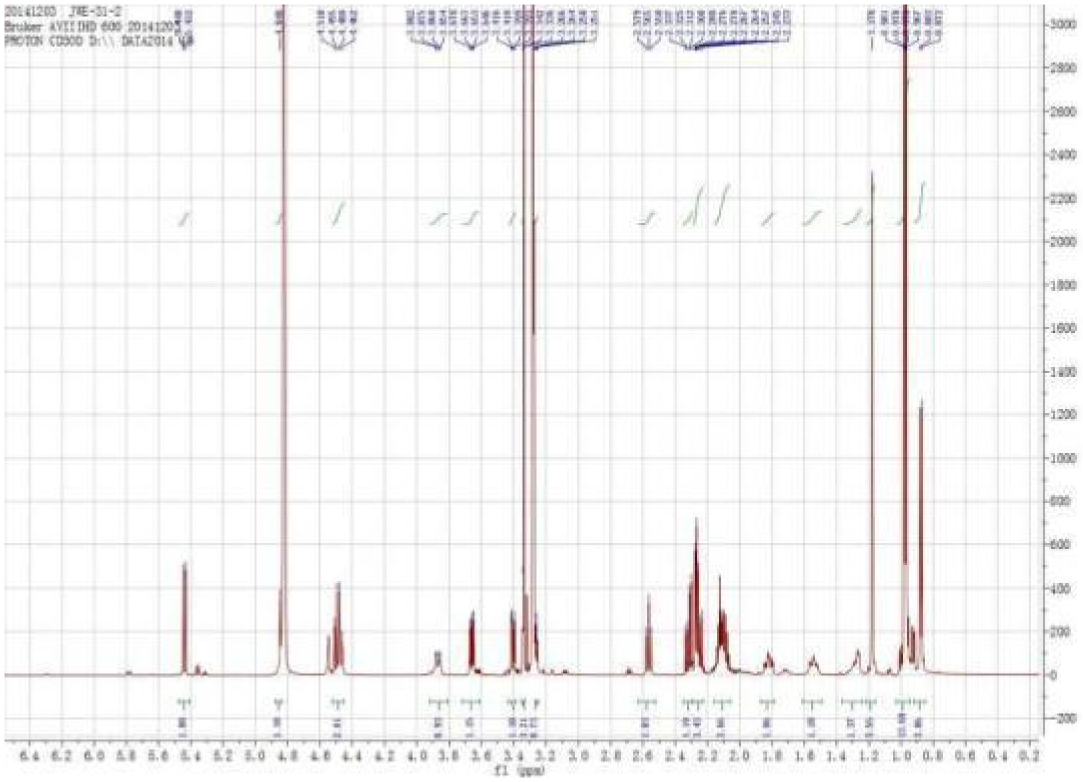

Figure S5.1 ^{1}H NMR spectrum of compound **5** in CD_3OD.

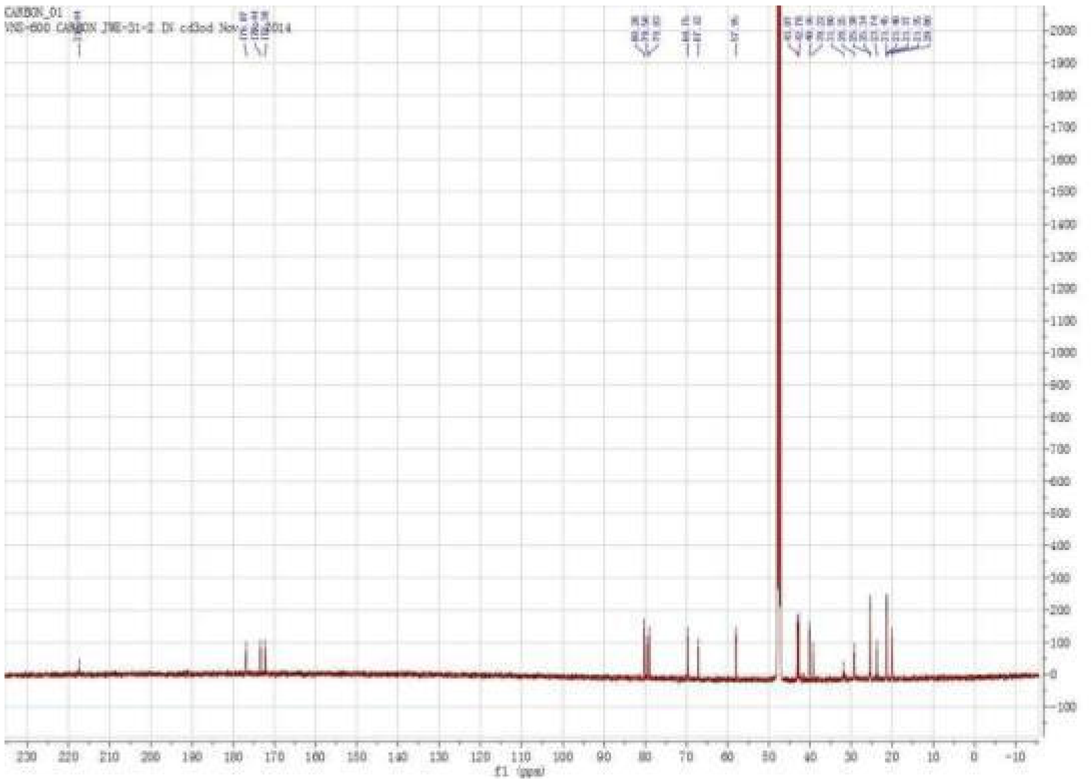

Figure S5.2 ^{13}C NMR spectrum of compound **5** in CD_3OD.

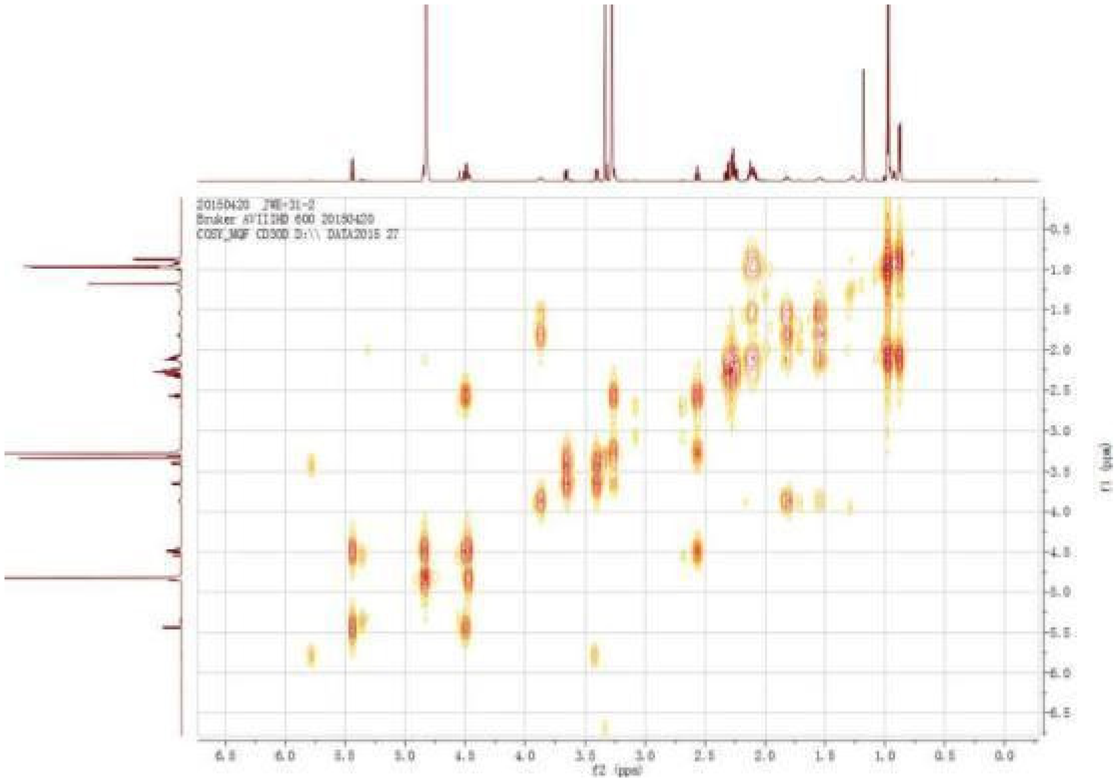

Figure S5.3 ^{1}H-^{1}H COSY spectrum of compound **5** in CD_3OD.

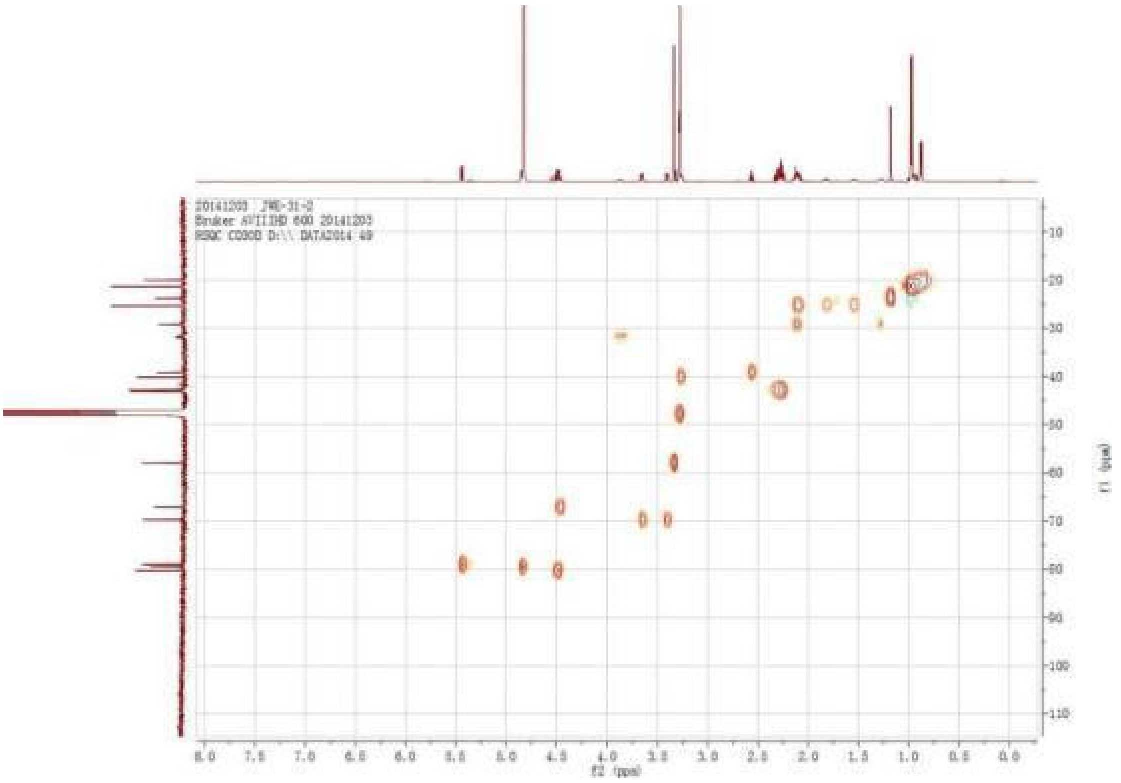

Figure S5.4 HSQC spectrum of compound **5** in CD_3OD.

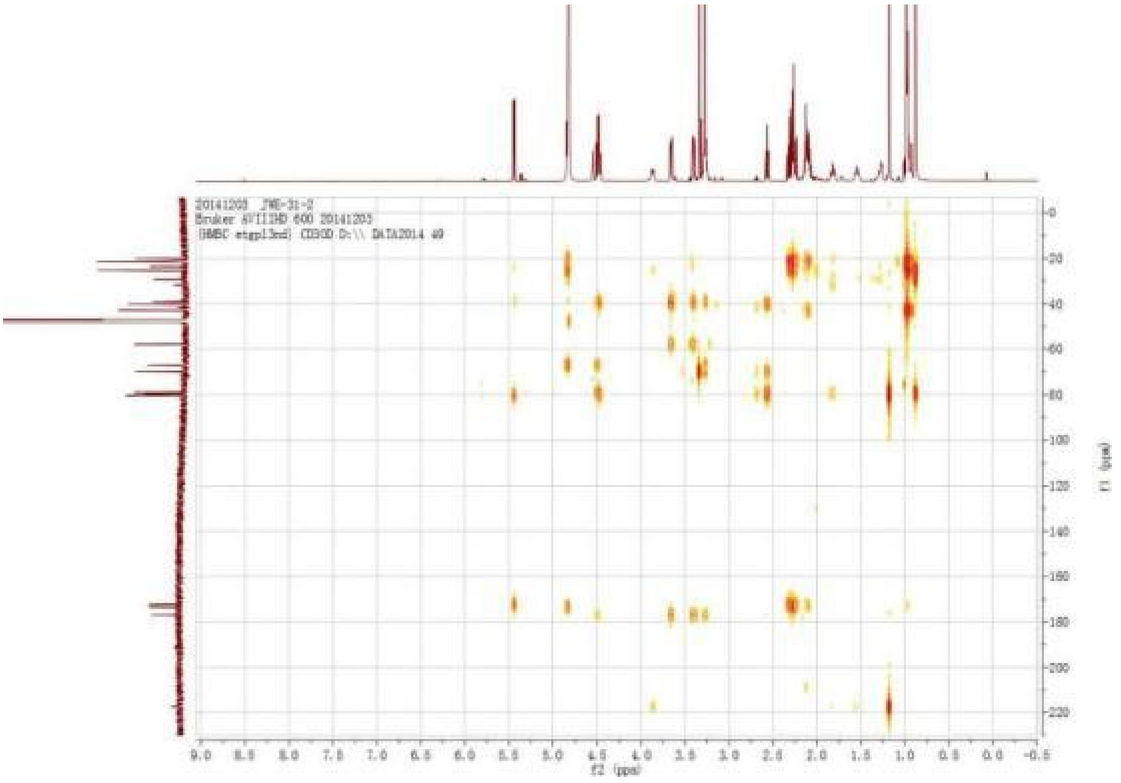

Figure S5.5 HMBC spectrum of compound **5** in CD_3OD.

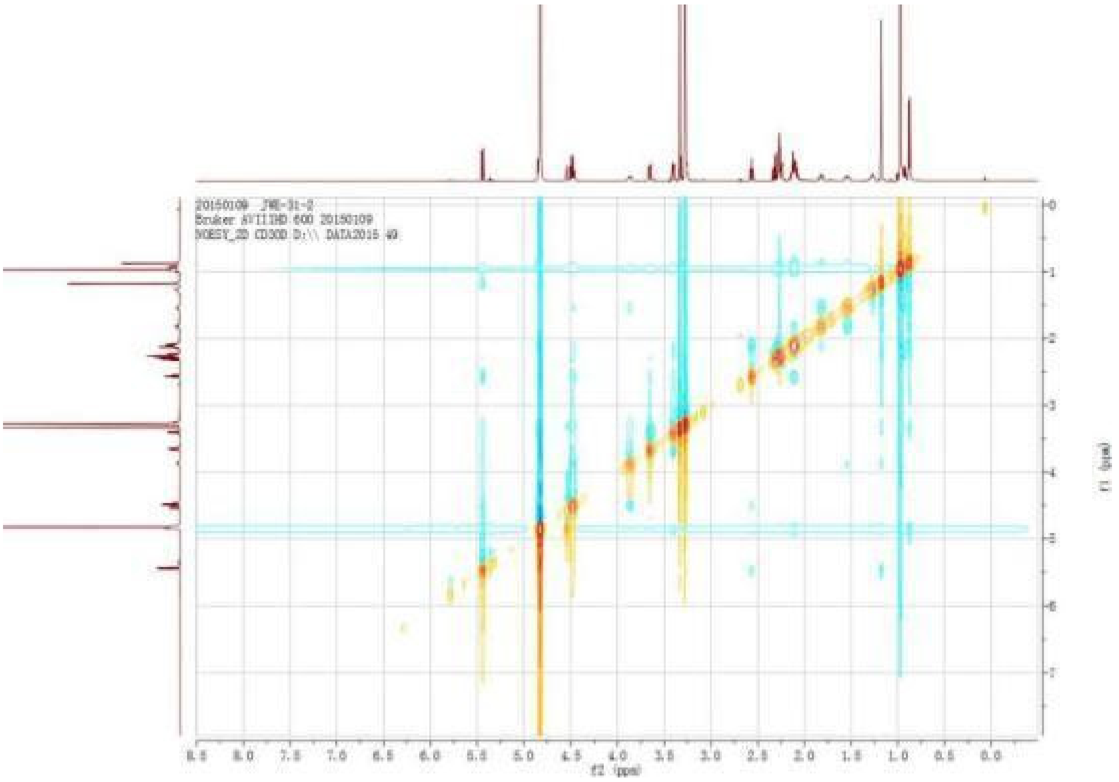

Figure S5.6 NOESY spectrum of compound **5** in CD_3OD.

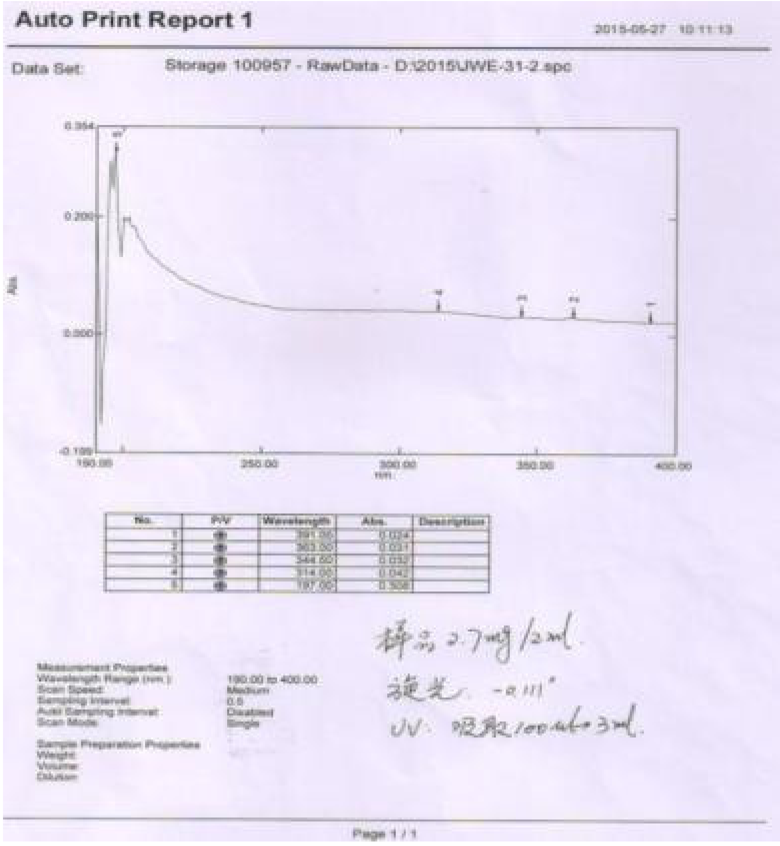

Figure S5.7 UV spectrum of compound **5**.

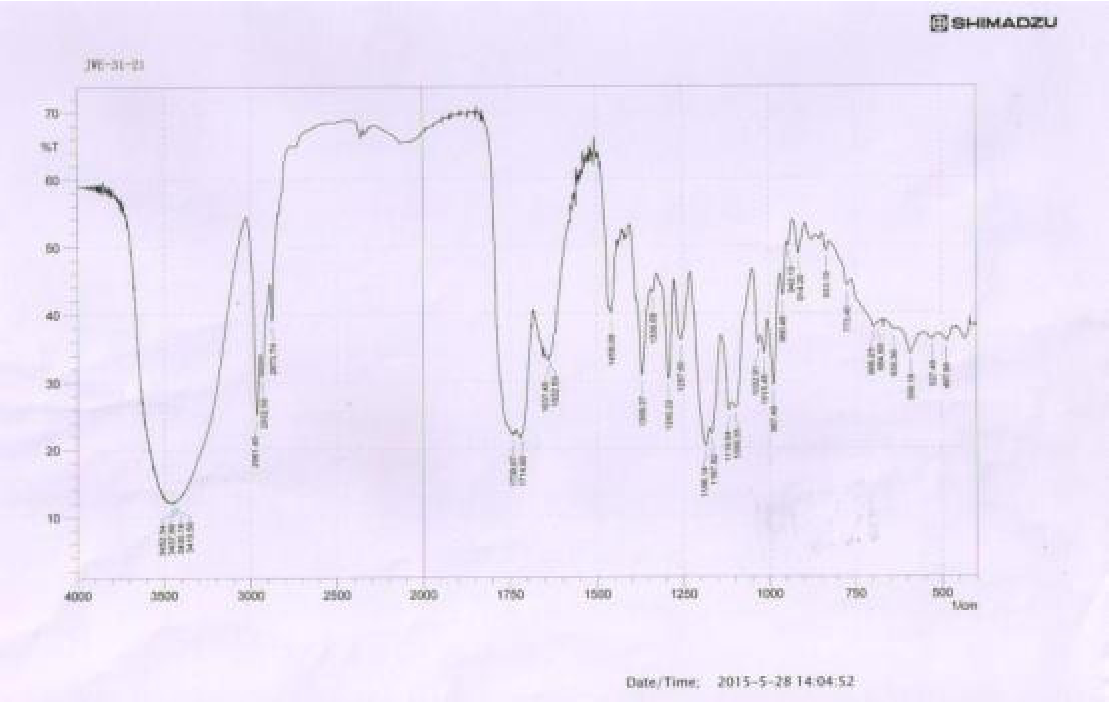

Figure S5.8 IR spectrum of compound **5**.

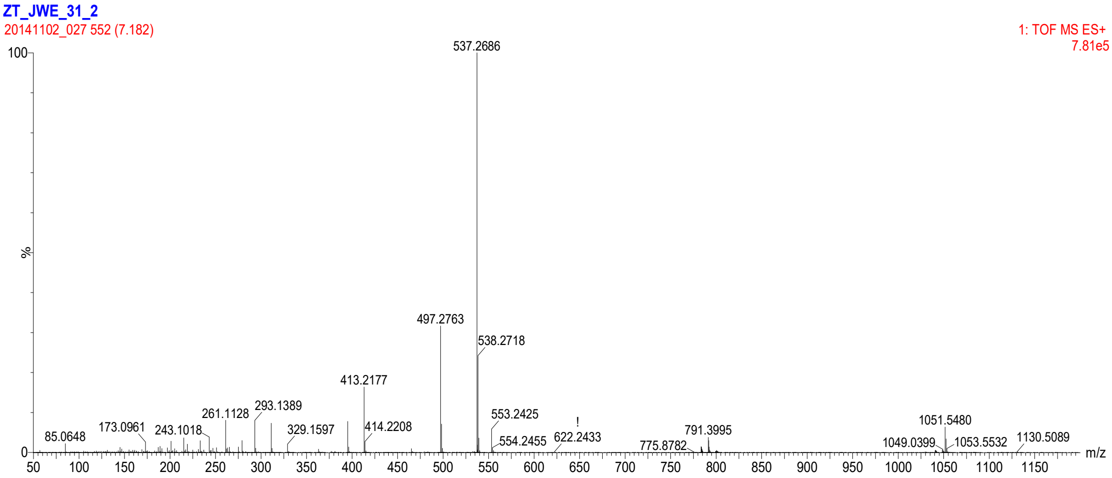

Figure S5.9 HRESIMS spectrum of compound **5**.

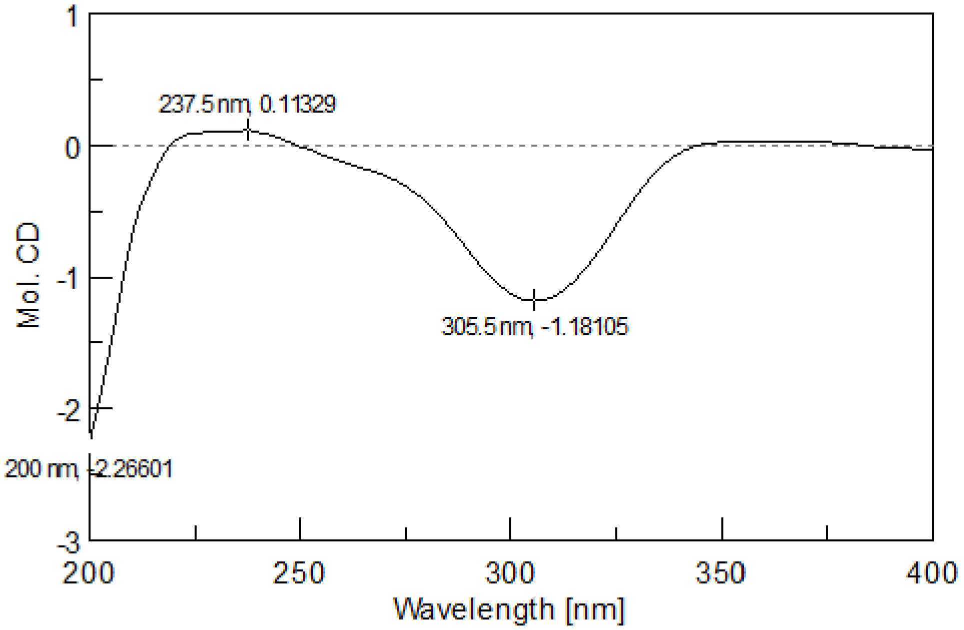

Figure S5.10 ECD spectrum of compound **5**.

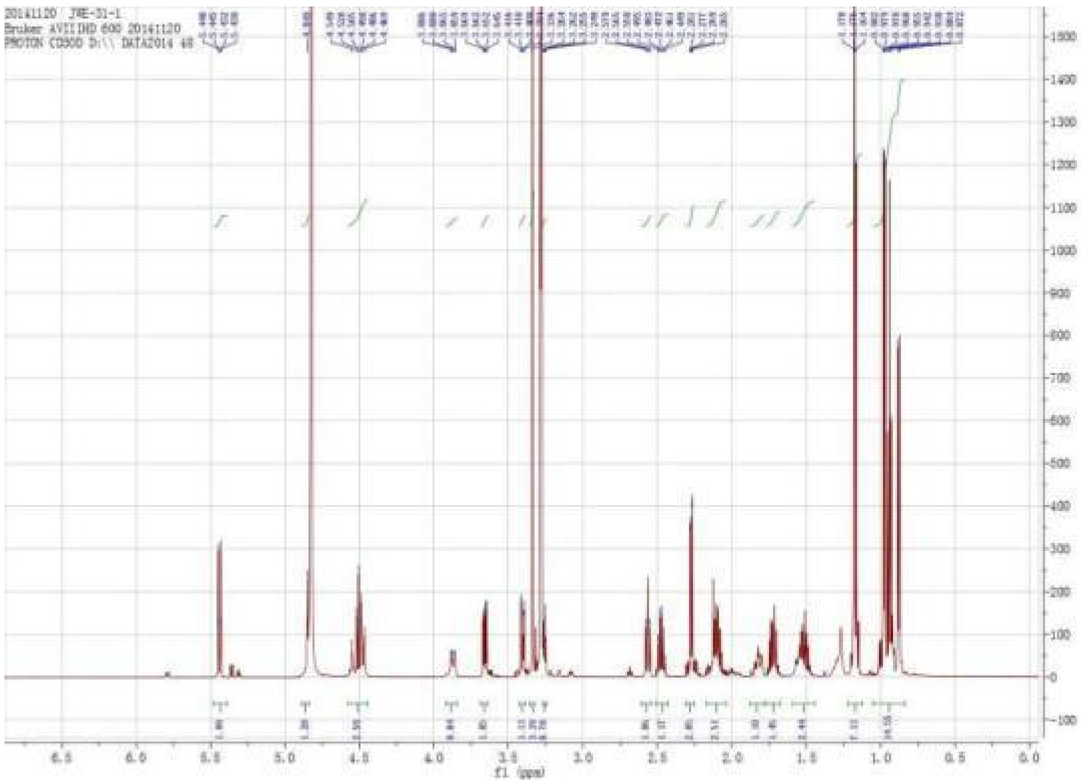

Figure S6.1 ^{1}H NMR spectrum of compound **6** in CD_3OD.

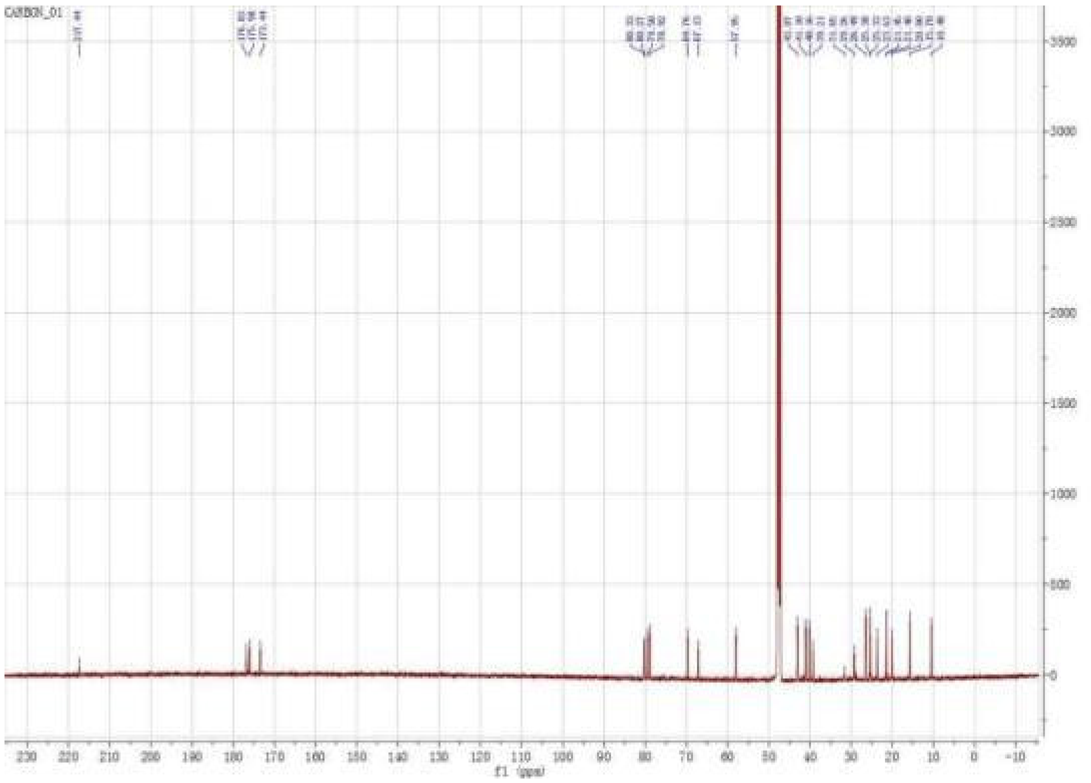

Figure S6.2 ^{13}C NMR spectrum of compound **6** in CD_3OD.

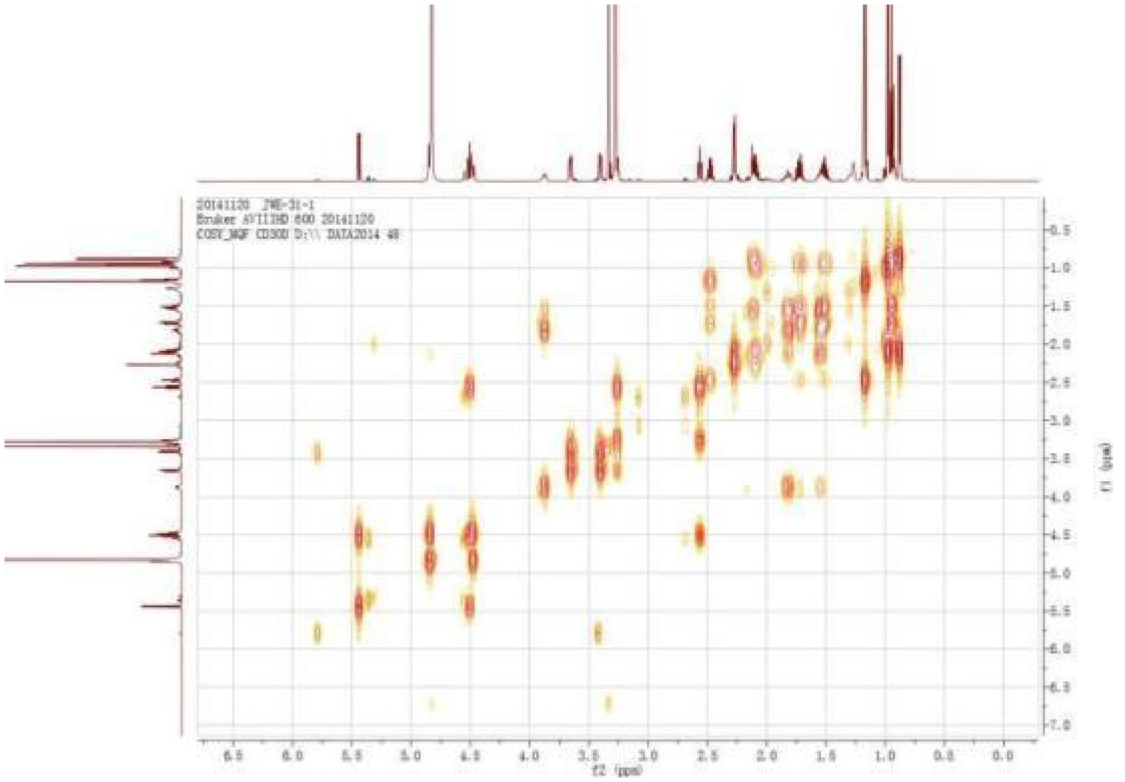

Figure S6.3 ^{1}H-^{1}H COSY spectrum of compound **6** in CD_3OD.

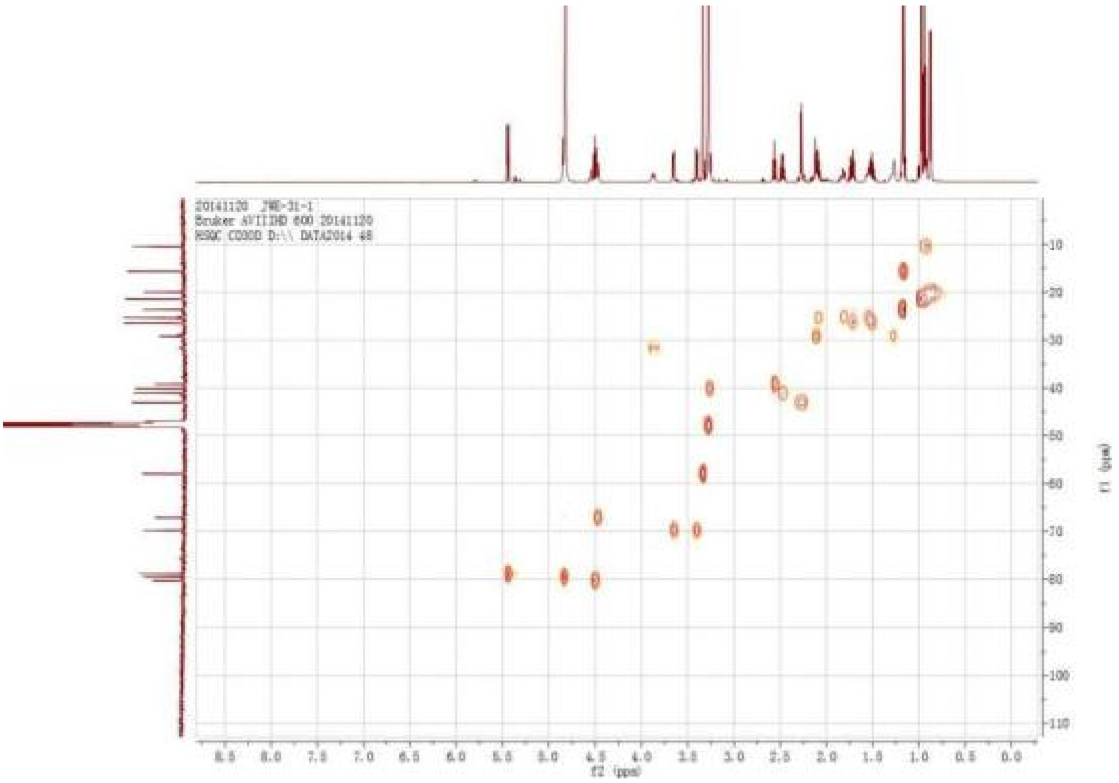

Figure S6.4 HSQC spectrum of compound **6** in CD_3OD.

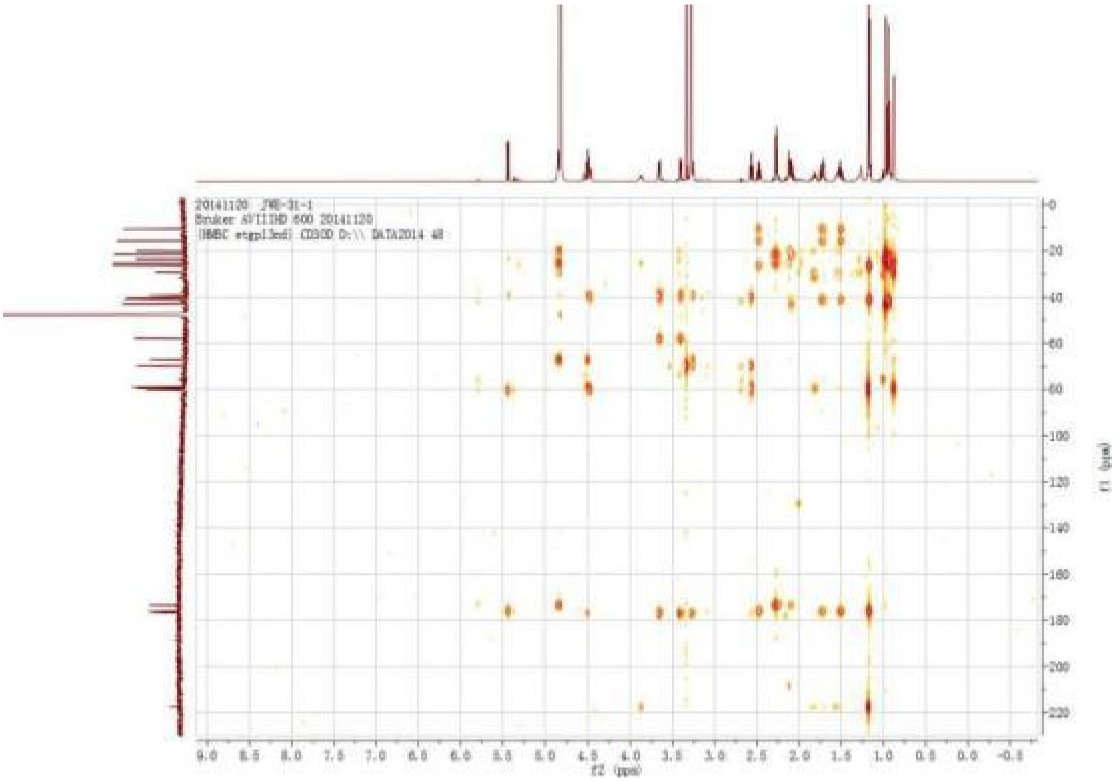

Figure S6.5 HMBC spectrum of compound **6** in CD_3OD.

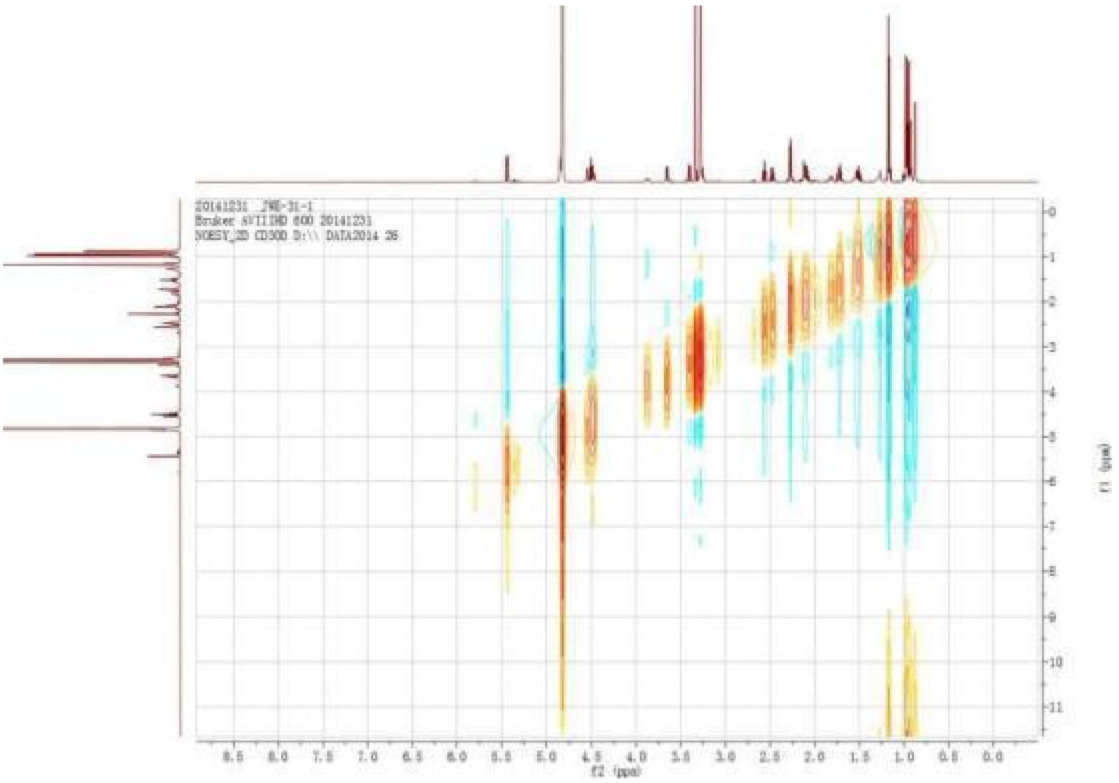

Figure S6.6 NOESY spectrum of compound **6** in CD_3OD.

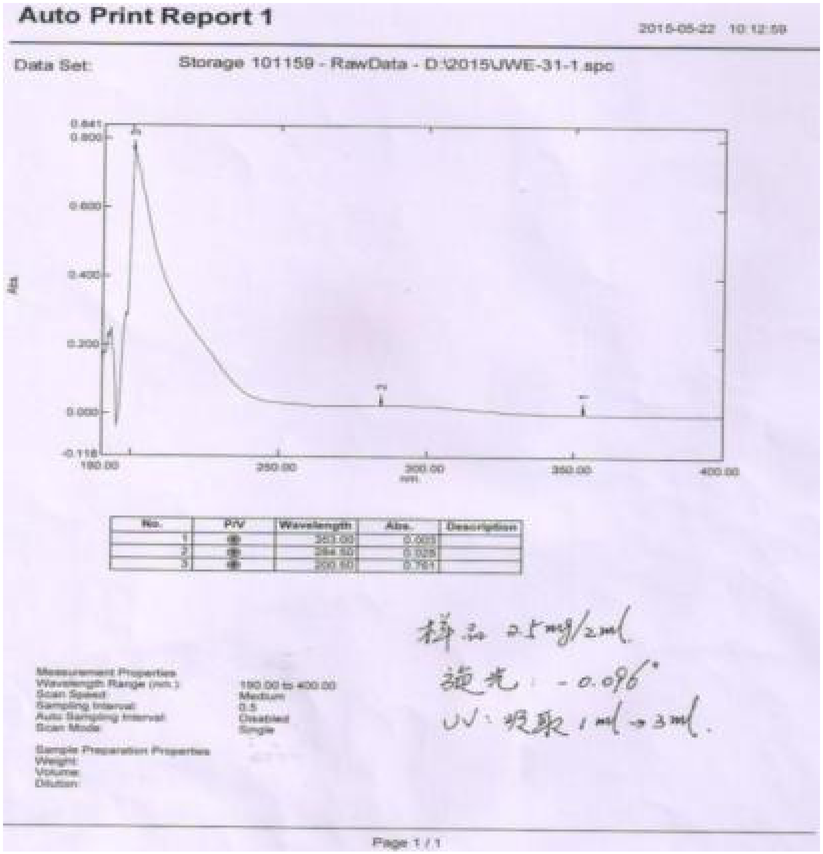

Figure S6.7 UV spectrum of compound **6**.

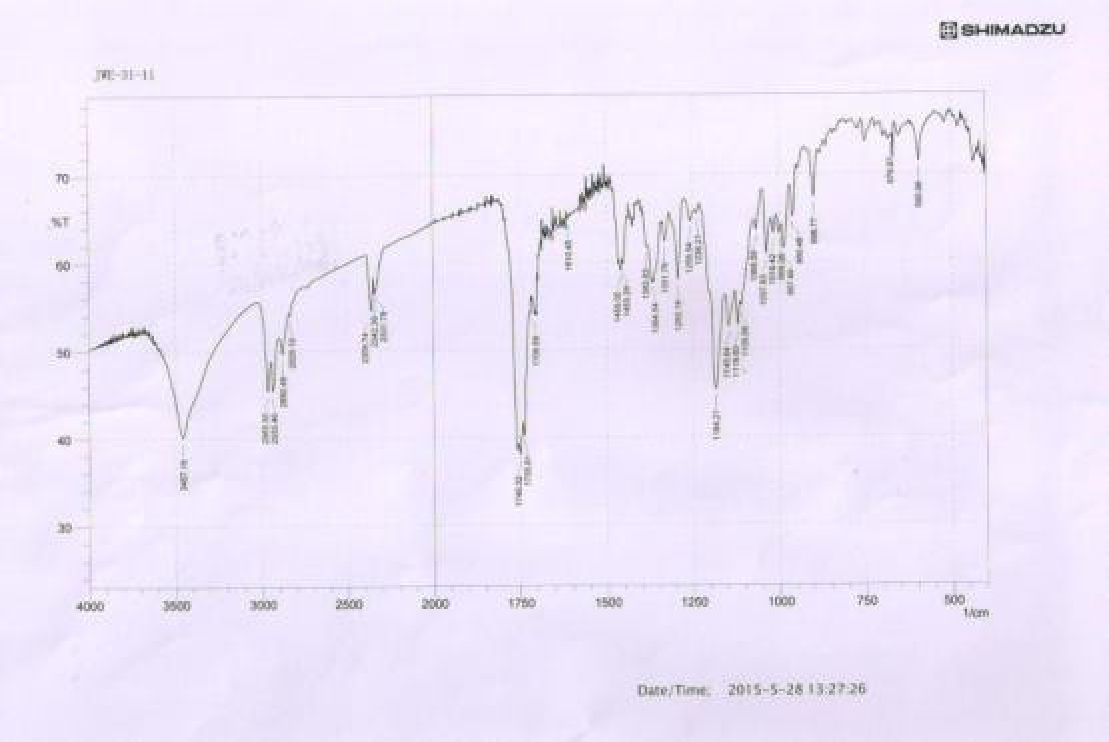

Figure S6.8 IR spectrum of compound **6**.

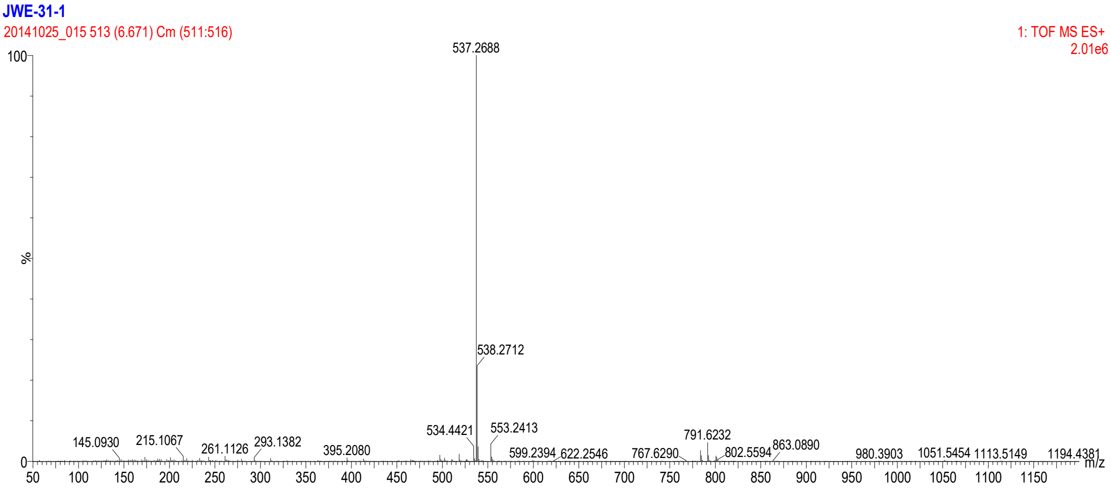

Figure S6.9 HRESIMS spectrum of compound **6**.

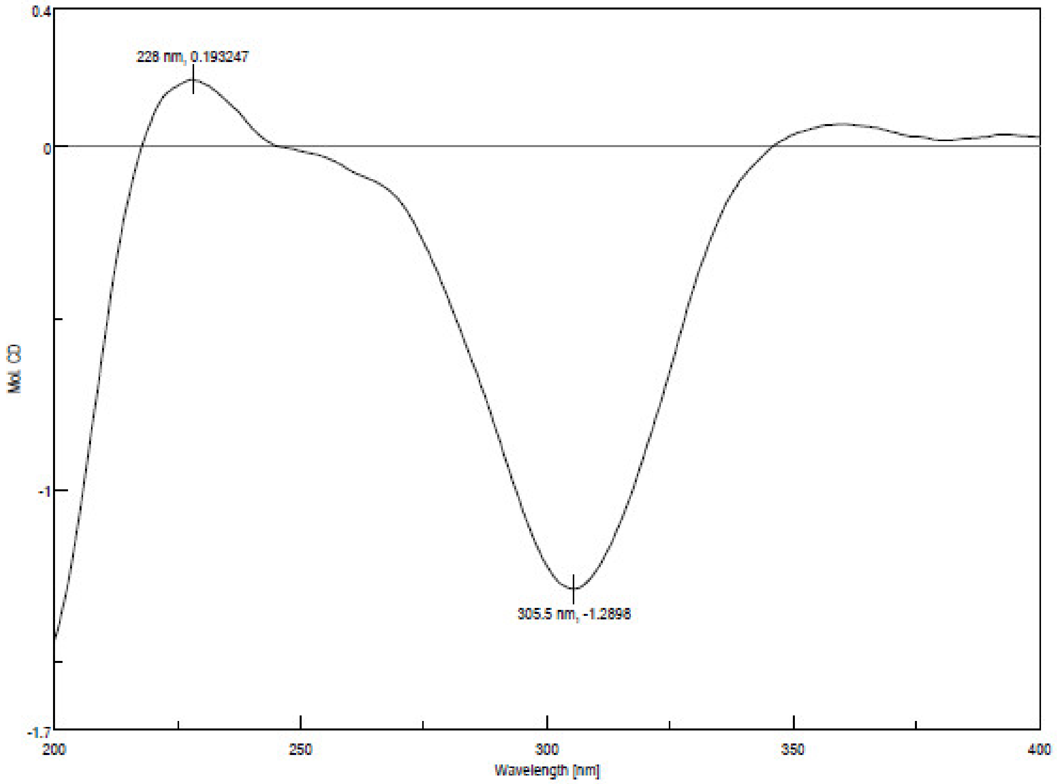

Figure S6.10 ECD spectrum of compound **6**.

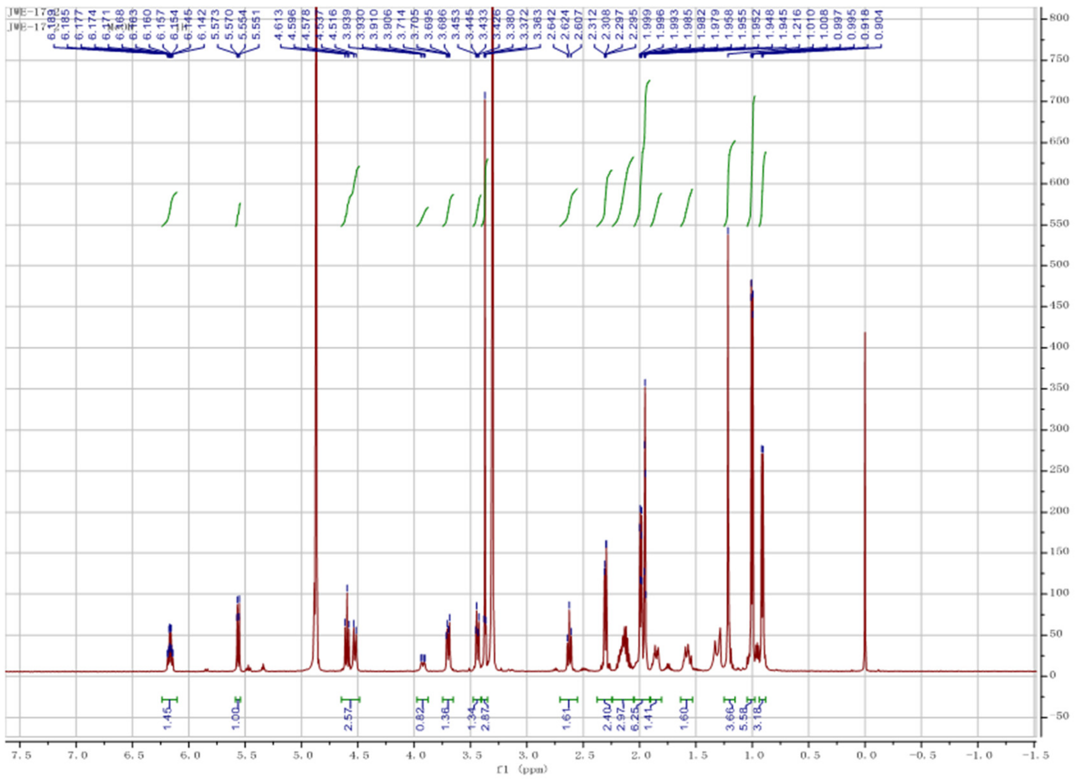

Figure S7.1 ^{1}H NMR spectrum of compound **7** in CD_3OD.

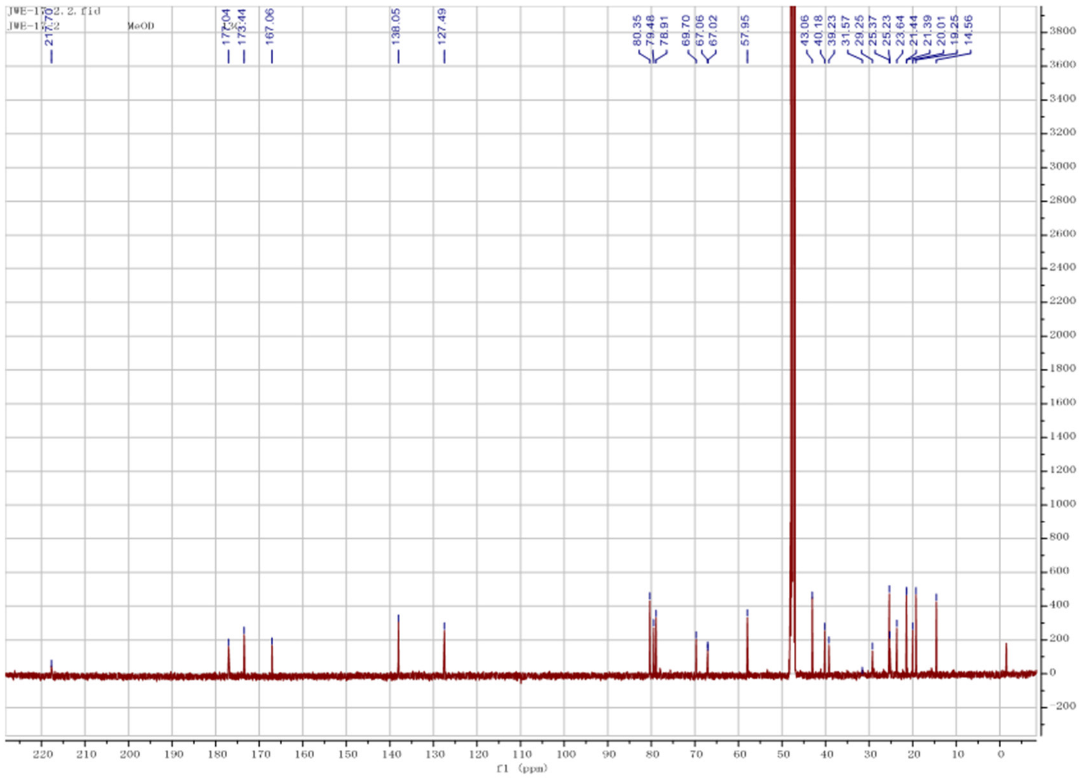

Figure S7.2 ^{13}C NMR spectrum of compound **7** in CD_3OD.

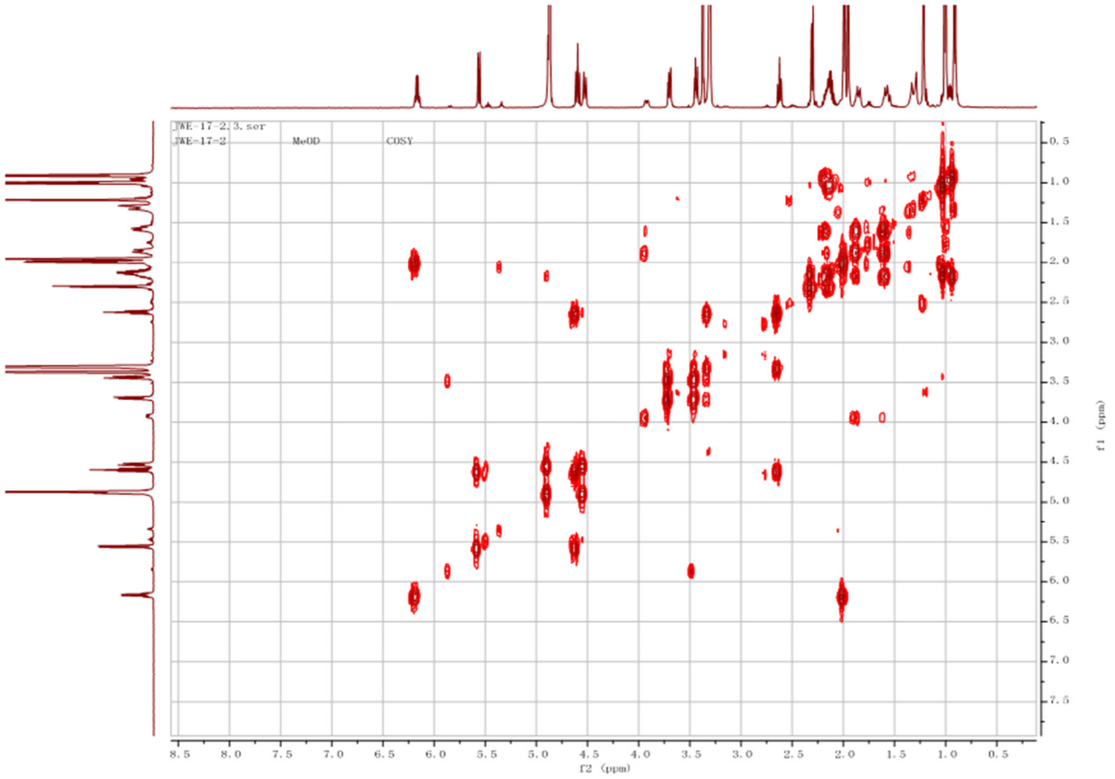

Figure S7.3 ^{1}H-^{1}H COSY spectrum (500 MHz) of compound **7** in CD_3OD.

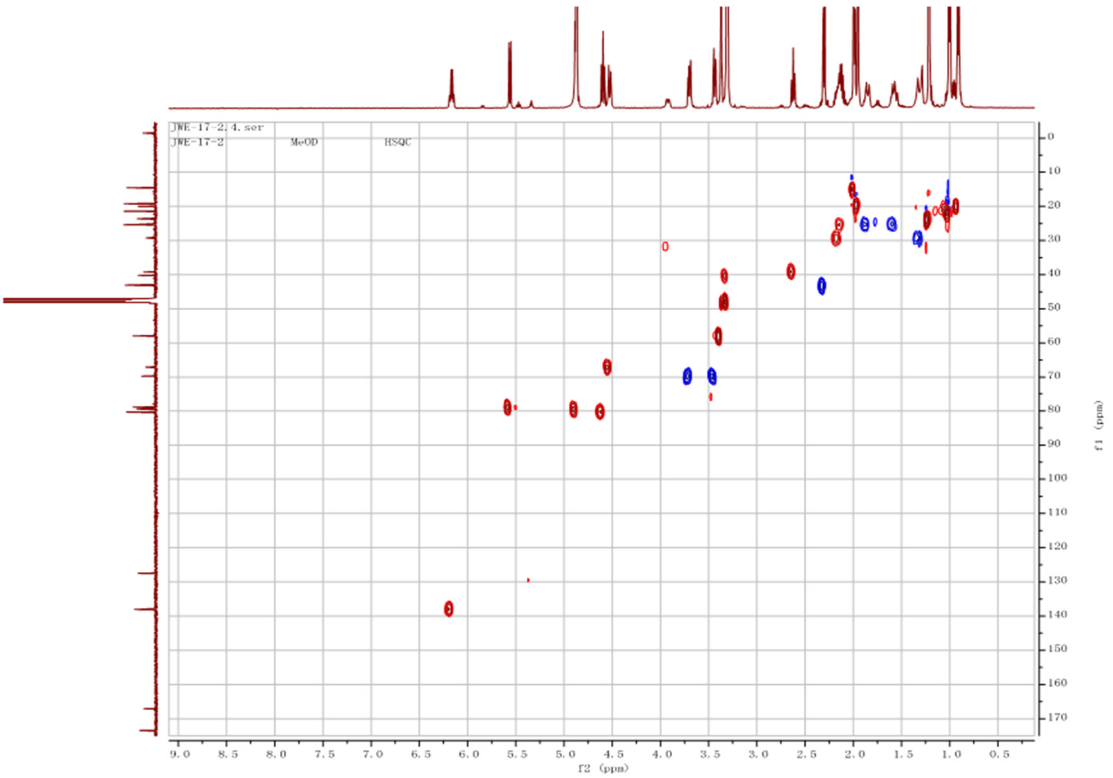

Figure S7.4 HSQC spectrum of compound **7** in CD_3OD.

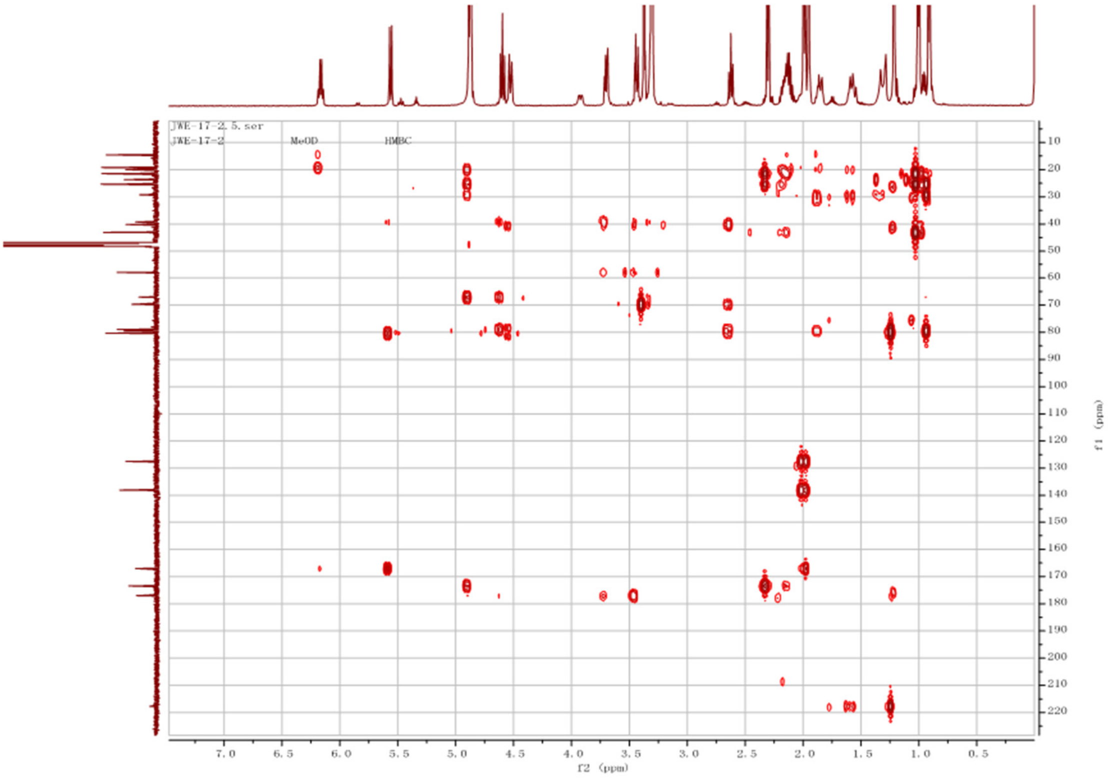

Figure S7.5 HMBC spectrum of compound **7** in CD_3OD.

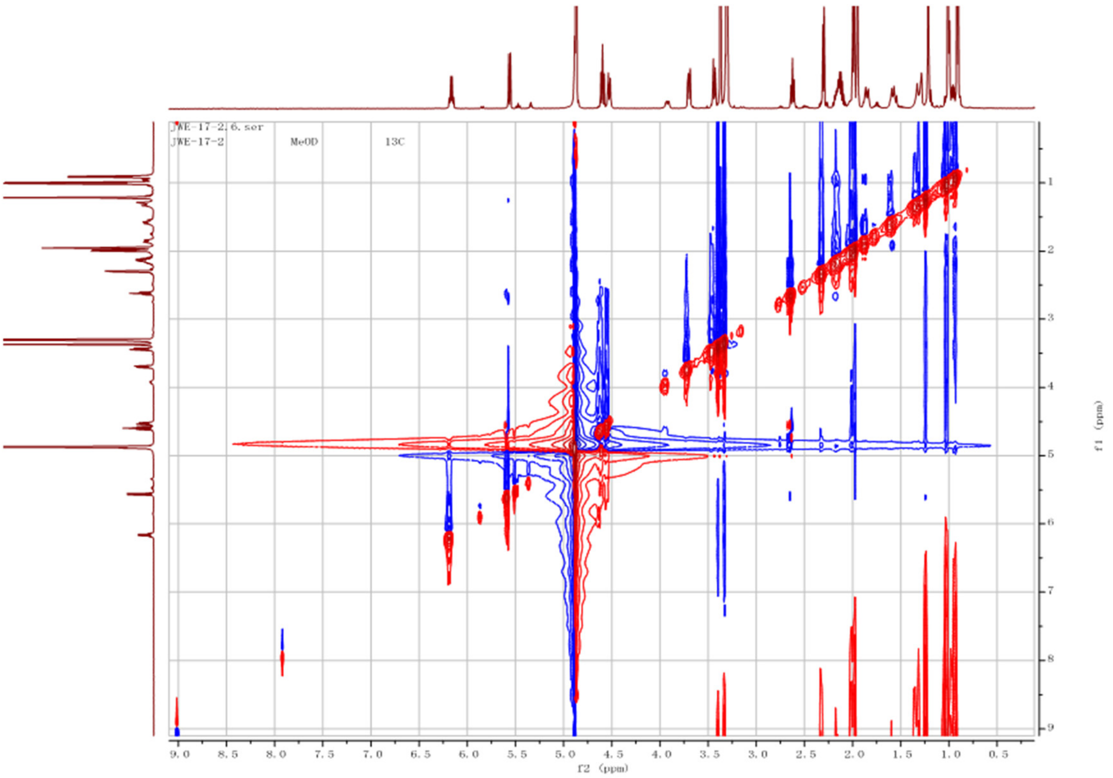

Figure S7.6 ROESY spectrum of compound **7** in CD_3OD.

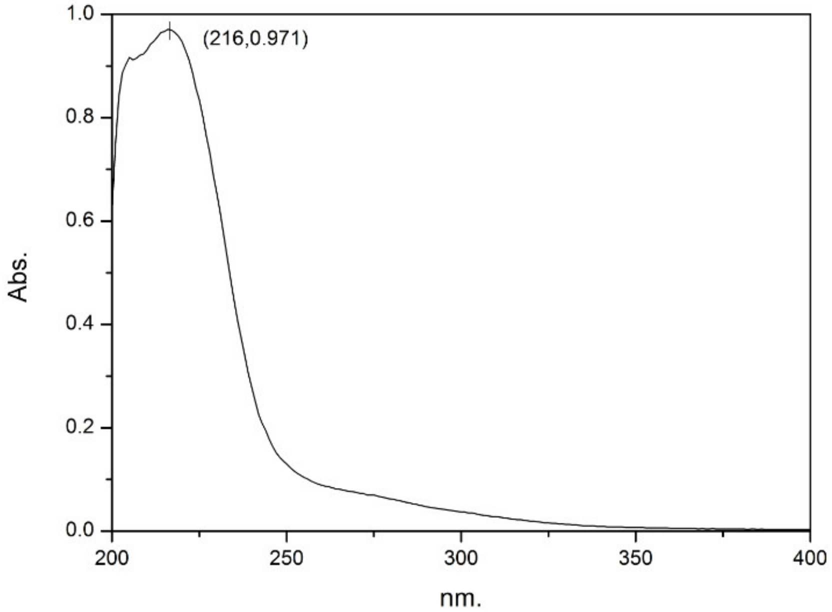

Figure S7.7 UV spectrum of compound **7**.

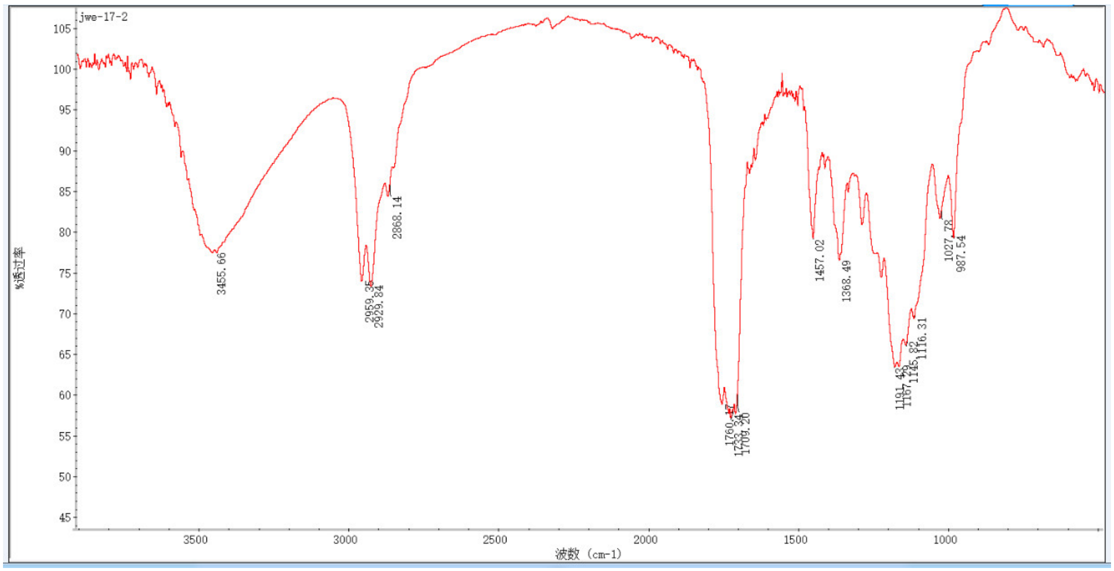

Figure S7.8 IR spectrum of compound **7**.

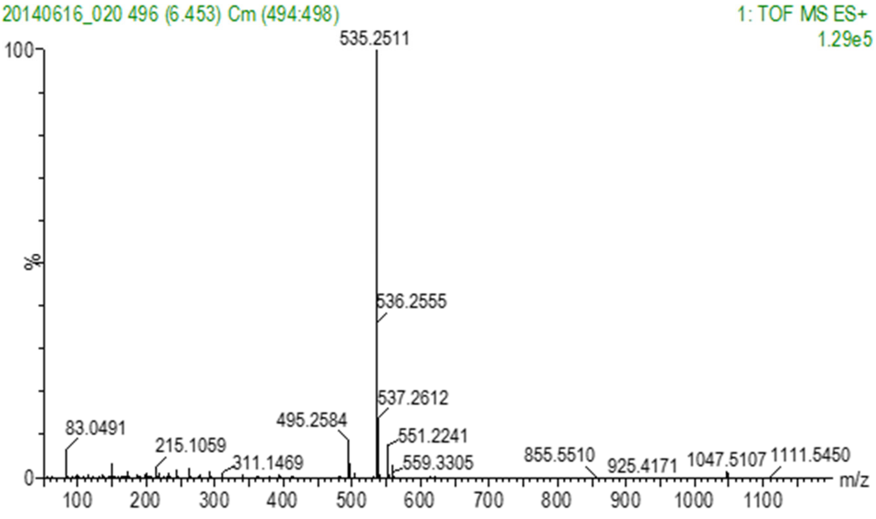

Figure S7.9 HRESIMS spectrum of compound **7**.

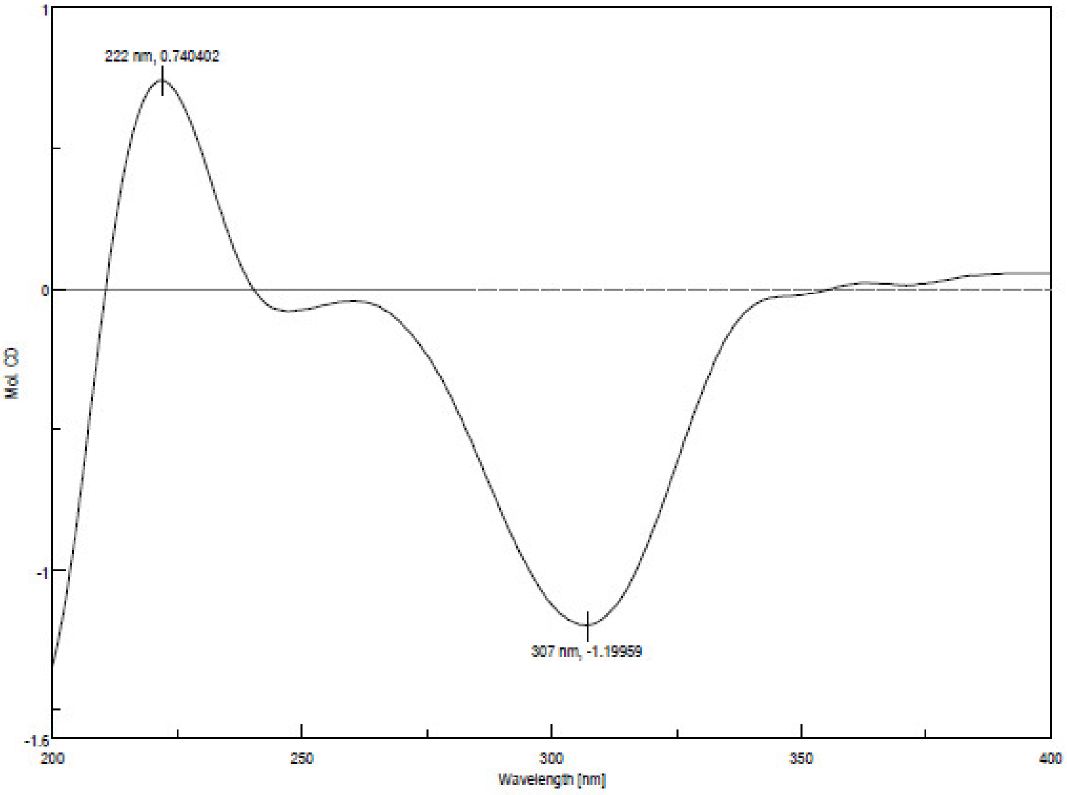

Figure S7.10 ECD spectrum of compound **7**.

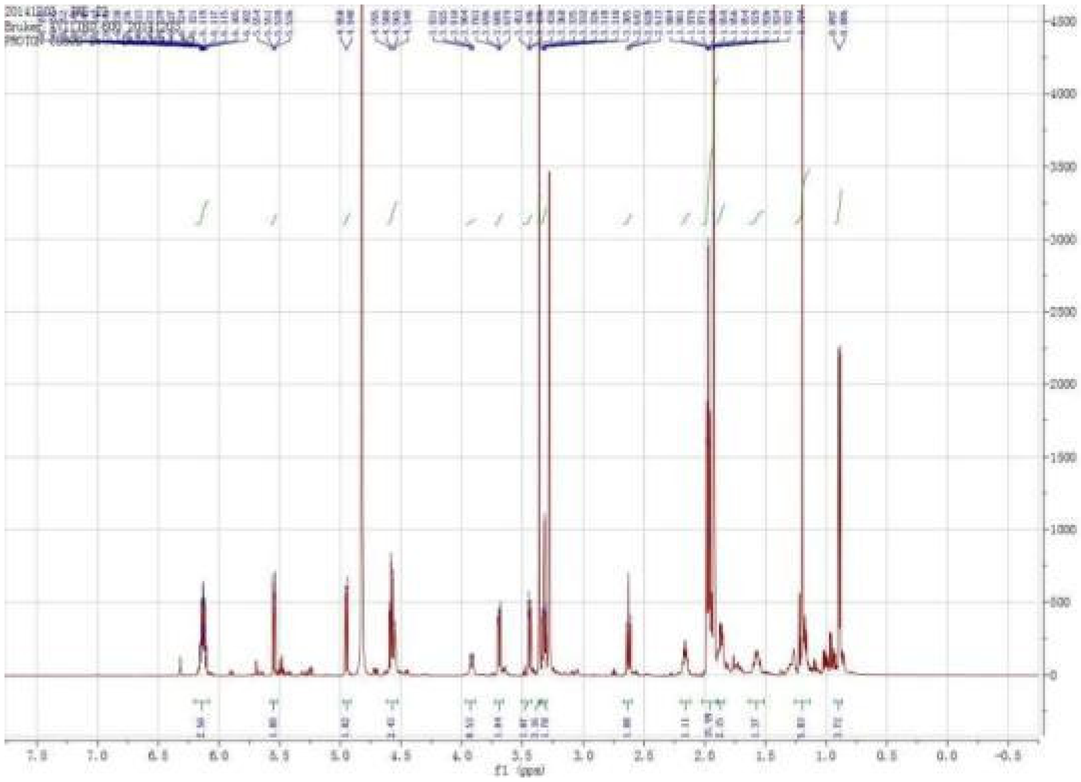

Figure S8.1 ^{1}H NMR spectrum of compound **8** in CD_3OD.

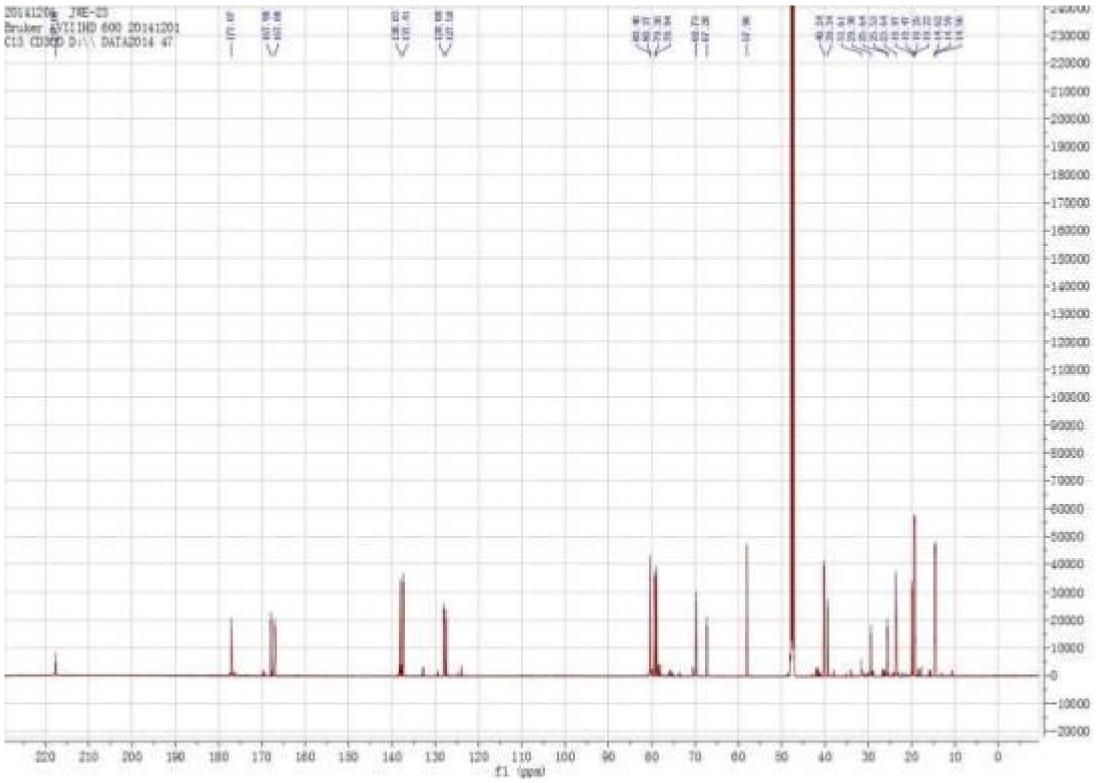

Figure S8.2 ^{13}C NMR spectrum of compound **8** in CD_3OD.

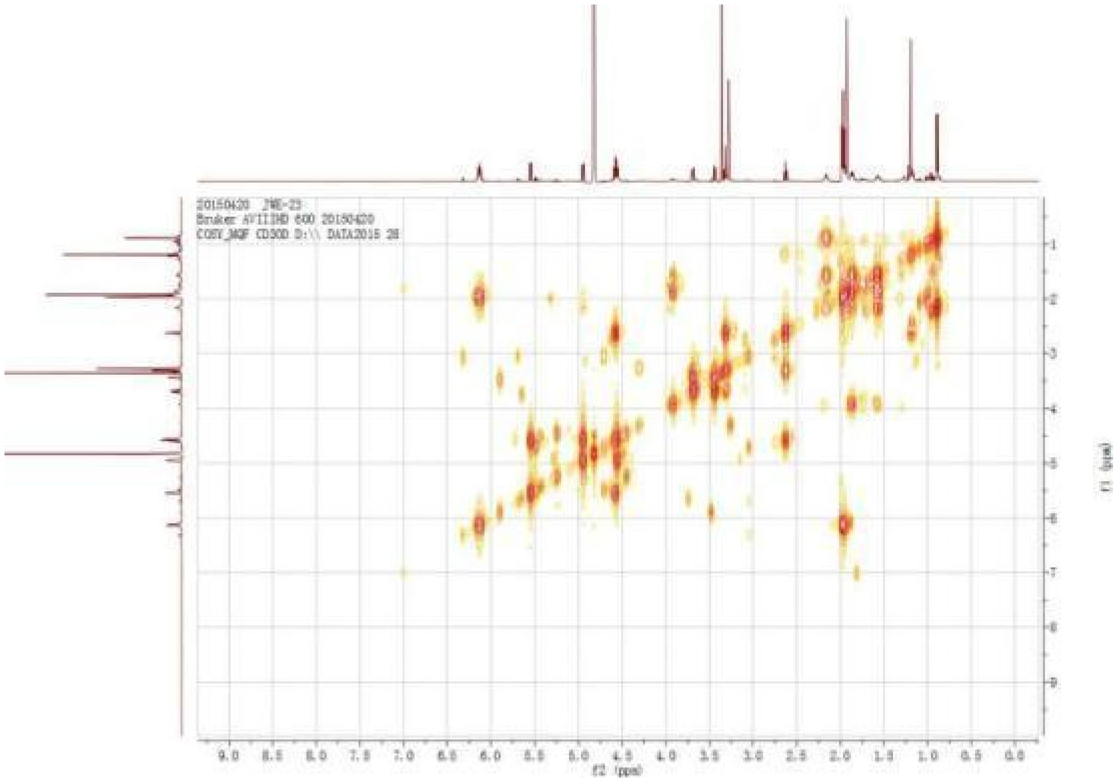

Figure S8.3 ^{1}H-^{1}H COSY spectrum of compound **8** in CD_3OD.

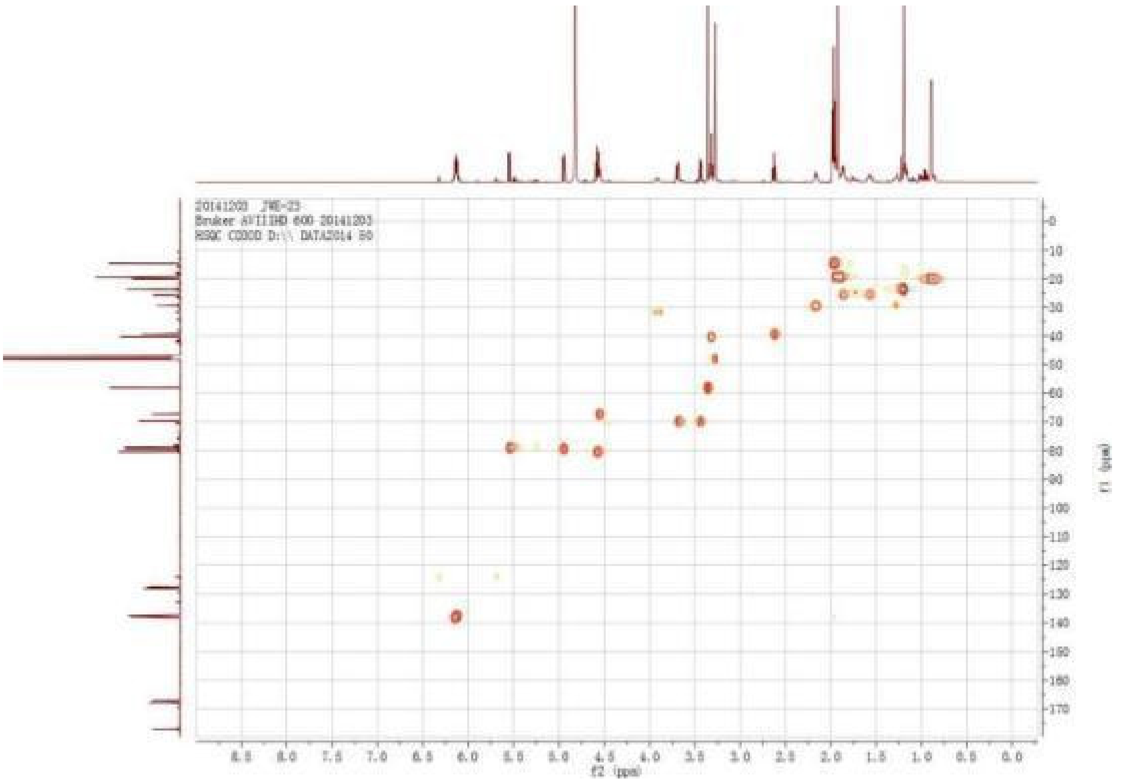

Figure S8.4 HSQC spectrum of compound **8** in CD_3OD.

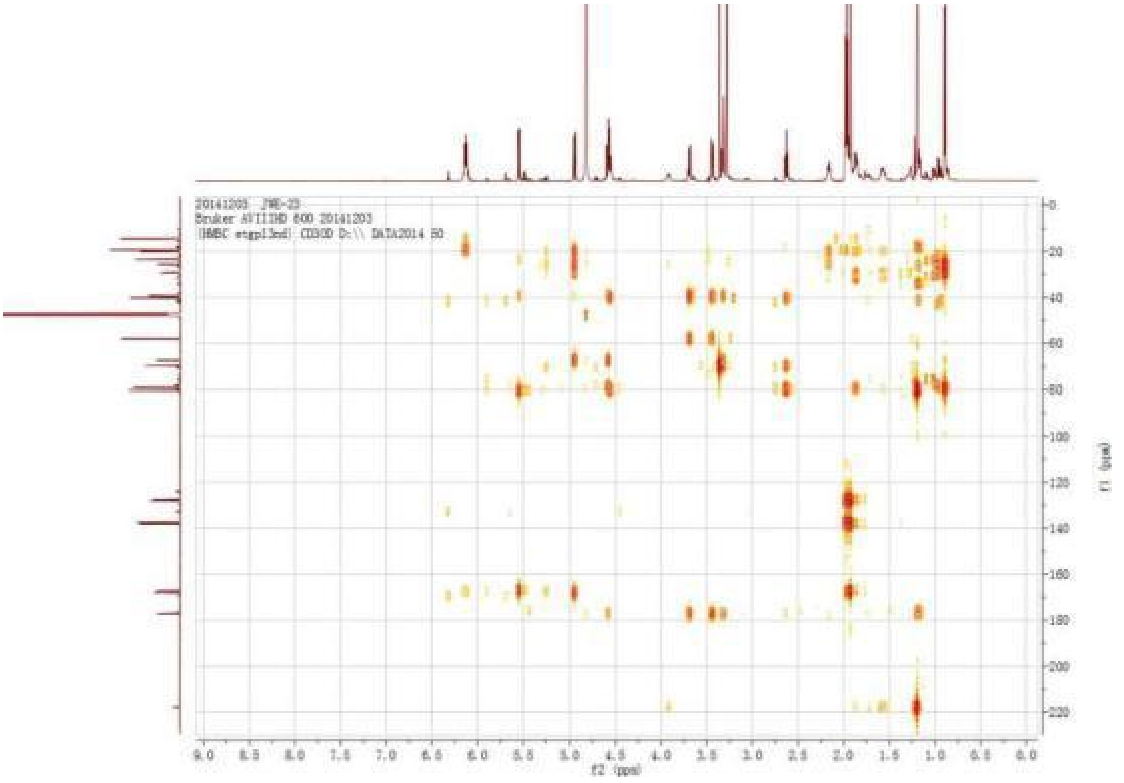

Figure S8.5 HMBC spectrum of compound **8** in CD_3OD.

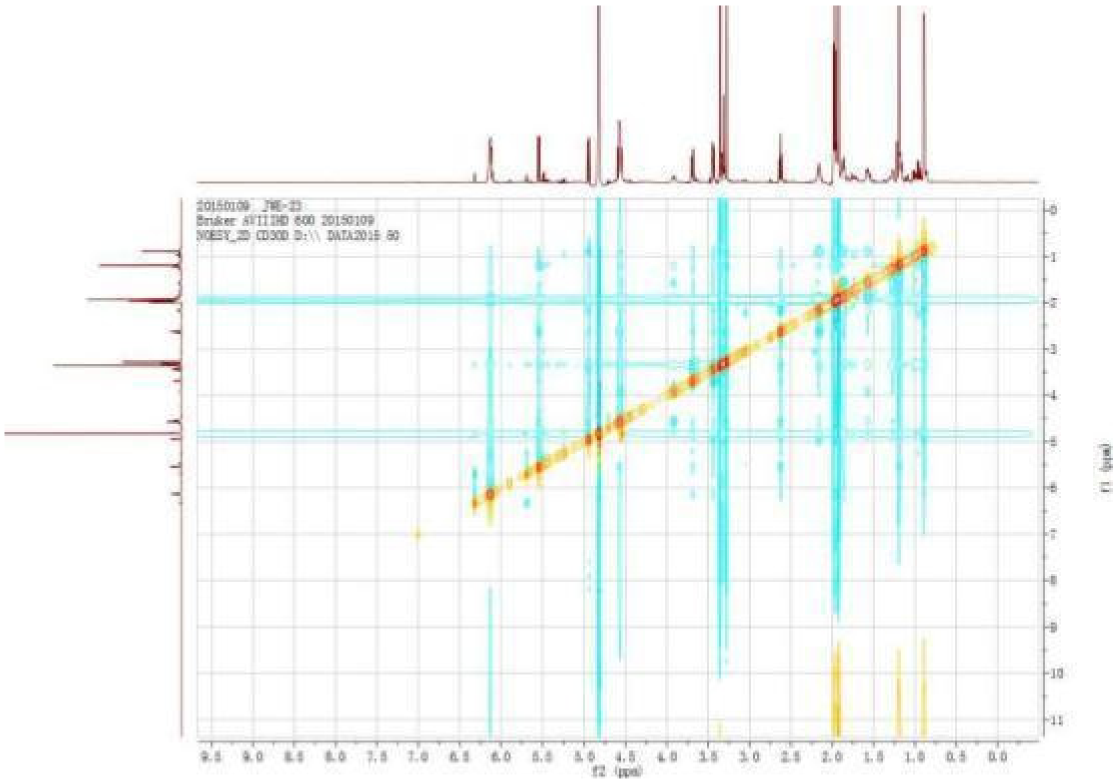

Figure S8.6 NOESY spectrum of compound **8** in CD_3OD.

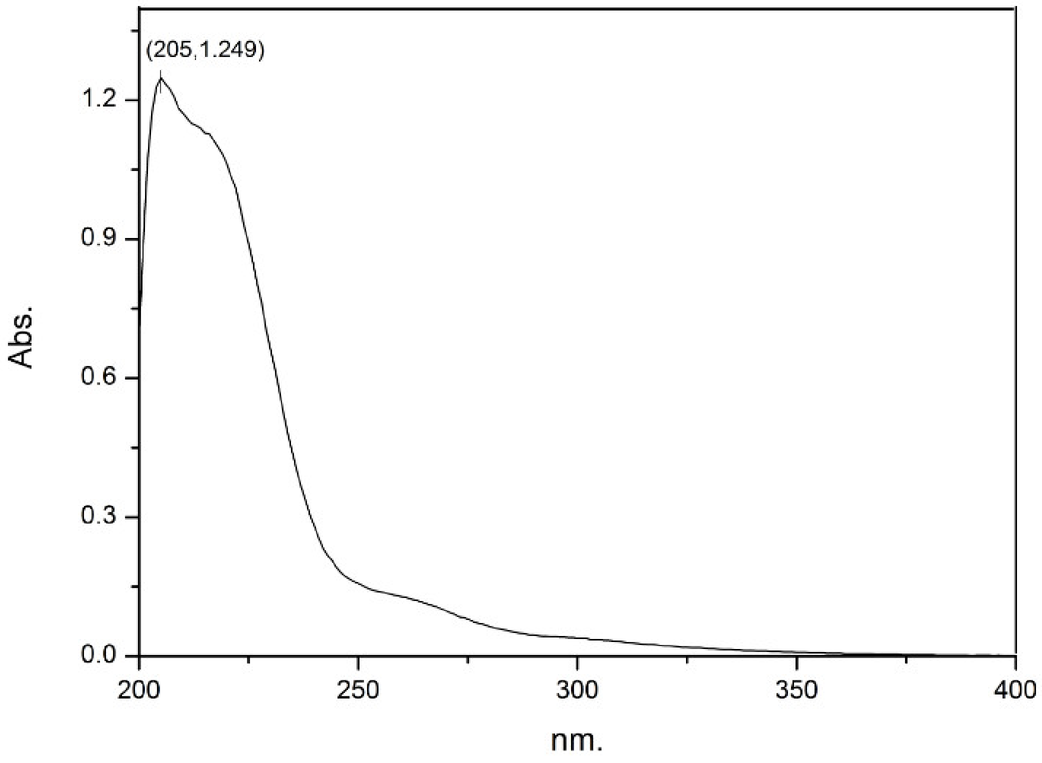

Figure S8.7 UV spectrum of compound **8**.

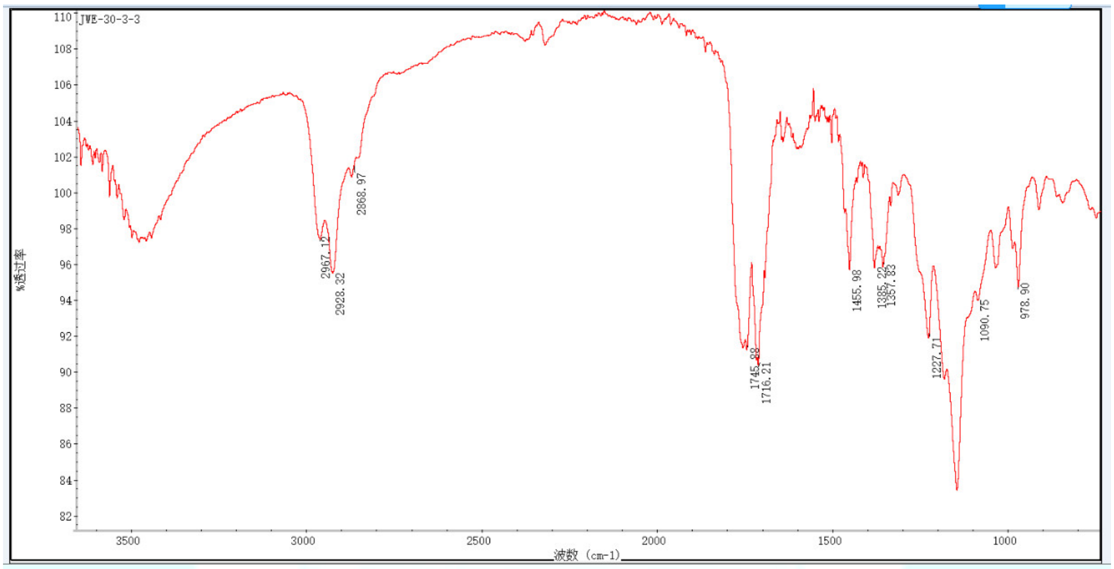

Figure S8.8 IR spectrum of compound **8**.

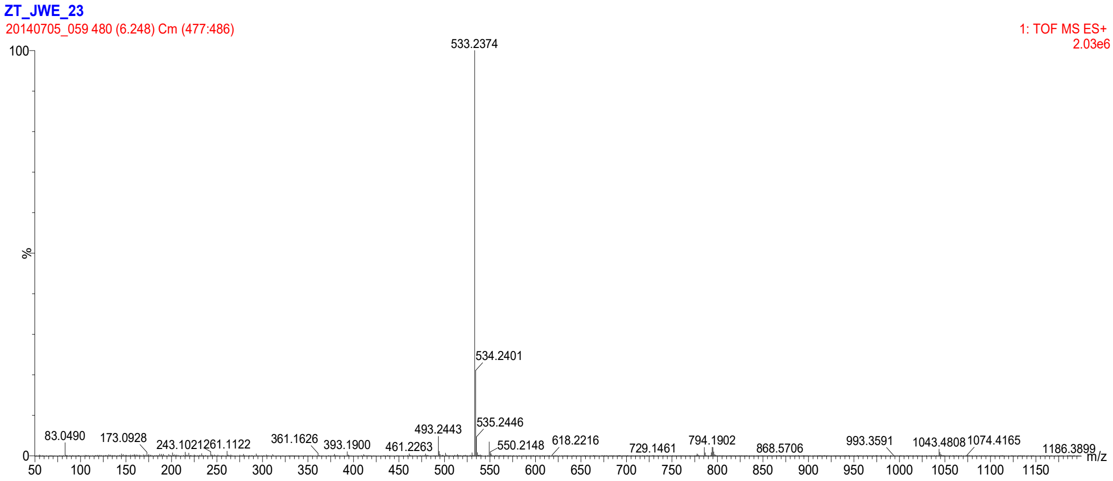

Figure S8.9 HRESIMS spectrum of compound **8**.

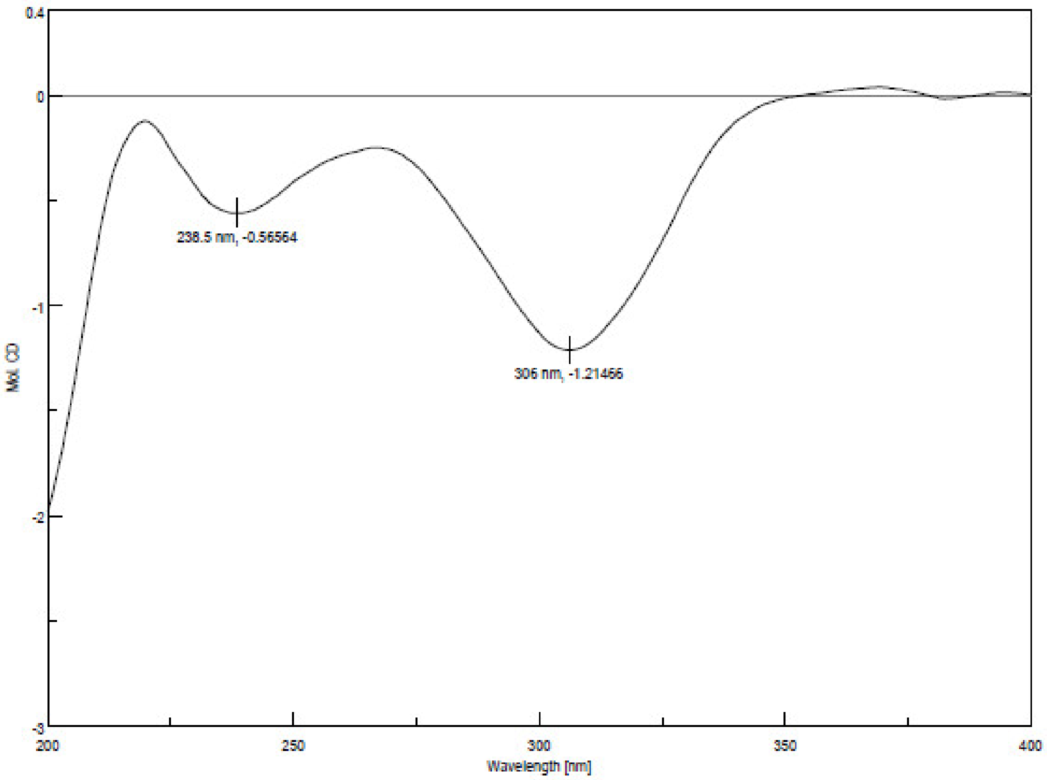

Figure S8.10 ECD spectrum of compound **8**.
